# Theonellamides J and K and 5-*cis*-Apoa-theopalauamide, Bicyclic Glycopeptides of the Red Sea Sponge *Theonella swinhoei*

**DOI:** 10.3390/md20010031

**Published:** 2021-12-27

**Authors:** Ohad Hasin, Shani Shoham, Yoel Kashman, Micha Ilan, Shmuel Carmeli

**Affiliations:** 1 Raymond and Beverly Sackler School of Chemistry, Faculty of Exact Sciences, Tel Aviv University, Ramat Aviv 6997801, Israel; ohadhasin@gmail.com (O.H.); Kashman@post.tau.ac.il (Y.K.); 2 School of Zoology, George S. Wise Faculty of Life Sciences, Tel Aviv University, Ramat Aviv 6997801, Israel; shanisho@yahoo.com (S.S.); milan@tauex.tau.ac.il (M.I.)

**Keywords:** sponge metabolites, *Theonella swinhoei*, glycopeptide, cytotoxicity, structure elucidation, NMR, MS

## Abstract

*Theonella swinhoei* is a fairly common inhabitant of reefs throughout the Indian and Pacific Oceans. Metabolomic analyses of samples of *T. swinhoei* collected in different depths in the Gulf of Aqaba revealed two chemotypes differing in the profiles of the theonellamides they produce, some of which seem to be unknown. Driven by this finding, we examined a sample of *T. swinhoei* collected more than 40 years ago in the southern part of the Gulf of Aqaba. Large-scale extract of this sample yielded four theonellamides, the known theopalauamide (**4**), as the major component, and three new metabolites, theonellamide J (**1**), 5-*cis*-Apoa-theopalauamide (**2**), and theonellamide K (**3**), as the minor components. The planar structure of these complex cyclic glycopeptides was elucidated by combination of 1D and 2D NMR techniques and HRESIMS. The absolute configuration of the amino acids was established by Marfey’s and advanced Marfey’s methods, and the absolute configuration of its galactose unit using “Tanaka’s method” for monosaccharides. The biological activity of the pure compounds was tested for antibacterial activity and for cytotoxicity to HTC-116 cell line. The compounds presented significant cytotoxicity against the HTC-116 cell line, illuminating the importance of the Apoa subunit for the activity.

## 1. Introduction

Marine sponges of the order Tetractinellida are rich in symbiotic bacteria and in biologically active natural products [1,2]. Sponges of the genus *Theonella* (family: Theonellidae), which belong to the order Tetractinellida, have been proven over the last five decades to be prolific producers of unique classes of biologically active natural products [3]. Sponge bioactive metabolites are believed to play a critical role in the survival of the sponge in its ecological niche by providing a chemical protection against massive predation, microbial pathogen, and fouling by sessile organisms [4]. *Theonella swinhoei*, is a fairly common inhabitant of reefs throughout the Indian and Pacific Oceans, from the north of the Red Sea along the African east coast down to South Africa and all the way east to Japan and New Zealand [5]. In the Gulf of Aqaba (Northern Red Sea), we have collected, in the last fifty years, samples of three *Theonella* spp.; white form of *T. swinhoei* (white interior, relatively common), yellow form of *T.* cf. *swinhoei* (yellow interior, extremely rare in deeper water), and *T. conica* (blue interior, fairly common). The unique 4-methylene sterols, conicasterol and theonellasterol, of the Red Sea *T. conica* and *T. swinhoei*, respectively, were studied in the early 1980s [6], while the white form *T. swinhoei* yielded the actin-disrupting cytotoxic bismacrolide, swinholide A [7,8]. Swinholide A was later shown to be produced in the sponge by its filamentous symbiotic bacterium, *Entotheonella* sp. [9], and more recently was also isolated from free-leaving *Symploca* cf. sp. marine cyanobacterium [10]. Interestingly, specimens of the white form of *T. swinhoei* collected in the Gulf of Aqaba have been shown to concentrate barium and arsenic (as arsenate) up to 10^6^ times their concentrations in the water body [11]. Most of the barium and arsenate salts are deposited as granules inside the symbiotic *Entotheonella* sp. Samples of the white form of *T. swinhoei* from Japan and the Philippines have not shown this characteristic behavior. Theopalauamide and theonegramide [12,13] were isolated as antifungal agents, while theonellamides A–G [14,15,16] were shown to be potent antifungal agents and equally potent cytotoxic against deferent cancer cell lines. Theonellamide I was shown to possess cytotoxicity against HeLa cell line [17]. The mode of action of theonellamides A and F was studied in a serious of studies. These studies demonstrated that theonellamides recognize and interact with 3β-hydroxysterols in lipid membranes and induce major morphological changes in cultured mammalian cells [18] and yeast [19] by activating Rho1-madiated 1,3-β-D-glucan synthesis [20]. Encouraged by the recent findings that the *Entotheonella* sp. recovered from a sample of the white form *T. swinhoei*, collected in the Gulf of Aqaba [21], contains genes responsible for the biosynthesis of both the swinholides and theonellamides, we decided to study the content of new samples of *T. swinhoei* recently collected by ROV in different depths, 10–64 m, at the northern part of the Gulf of Aqaba and samples collected more than 40 years ago in the southern part of the Gulf of Aqaba. From the later samples we isolated four theonellamides. The structure elucidation and biological activity of three new theonellamides is described below.

## 2. Results

LCMS analysis of the extracts of the *T. swinhoei* samples, collected at depth of 10–64 m, revealed that they contain, along with the known swinholide A, some unknown swinholides, and theonellamides. Furthermore, we found that there are two chemotypes, one from shallow water that contains one major theonellamide, theopalauamide, much smaller amounts of additional two theonellamides and traces of potentially other theonellamides (Figure S1 and Table S1), and the second one, which appear in deeper waters that contains, in addition to theopalauamide, a large variety of other known and unknown theonellamides (Figure S1). Triggered by these findings, we performed LCMS analyses to extracts of *T. swinhoei* samples we had in our inventory (Figure S2). The LCMS analyses of extracts of the original samples of *T. swinhoei*, collected by us more than 40 years ago in the southern part of the Gulf of Aqaba, revealed that they also contain uncharacterized theonellamides.

**Figure 1:**
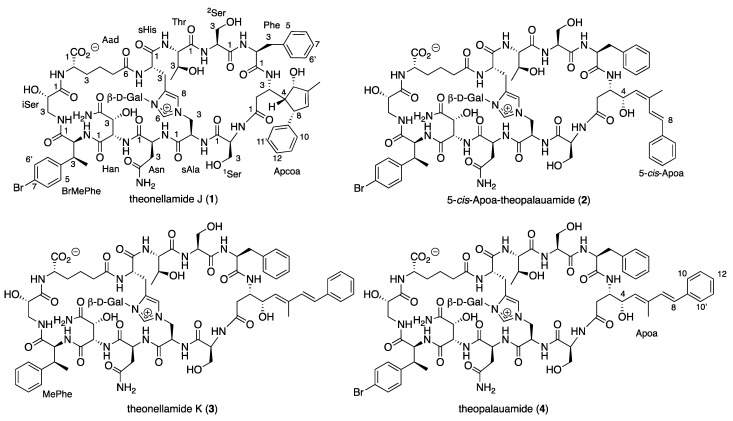
Structure of compounds **1**–**4**.

Large-scale 9:9:2 EtOAc/MeOH/H_2_O extract of the sponge *T. swinhoei* (order Tetractinellida, family Theonellidae), sampling #1156 which was collected in February 1982 by scuba diving at a depth of 20 m in the southern part of the Gulf of Aqaba (freeze-dried material that was stored in deep freezing since then), followed by several steps of purification afforded four pure compounds, three of which presented identical negative ESI MS ion and molecular formula: the known theopalauamide [12] (**4**, *m*/*z* 1745.5978, [M − H]^−^, C_76_H_98_^79^BrN_16_O_27_, the major theonellamide), theonellamide J (**1**, *m*/*z* 1747.6157, [M + H]^+^, C_76_H_100_^79^BrN_16_O_27_), and 5-*cis*-Apoa-theopalauamide (**2**, *m*/*z* 1769.5944, [M + Na]^+^, C_76_H_99_^79^BrN_16_O_27_Na), and the fourth theonellamide K (**3**, *m*/*z* 1669.7034, [M + H]^+^, C_76_H_101_N_16_O_27_) (Figure 1).

Due to the complex structure of the theonellamides, it was hard to conclude just from comparison of the NMR data whether **4** is identical with theopalauamide or different [12]. To prove it, we fully characterize it by running its 1D and 2D NMR spectra in 4:1 DMSO-*d*_6_:H_2_O at 50 °C (the NMR solvent suggested by Matsunaga et al. [17], see Table S2 and Figures S3–S12 in Supporting Material) and established the absolute configuration of its amino acids (excluding Apoa) by Marfey’s [22] and advanced Marfey’s [23] methods and the absolute configuration of its galactose unit using “Tanaka’s method” for monosaccharides [24]. Based on these analyses, compound **4** was identified as theopalauamide [12].

**Figure 2:**
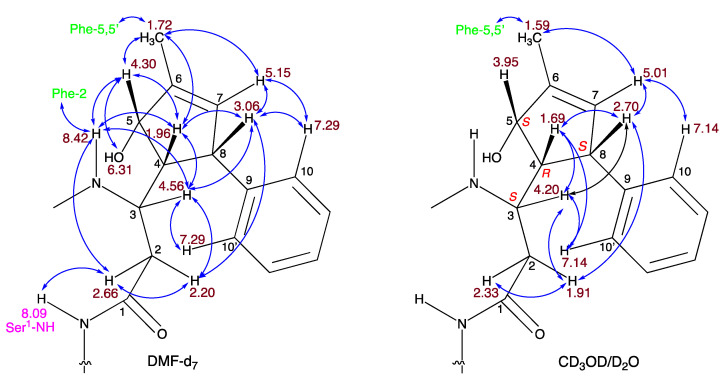
NOE correlations of **1** in DMF-*d*_7_ and 4:1 DMSO-d_6_/D_2_O.

**Figure 3:**
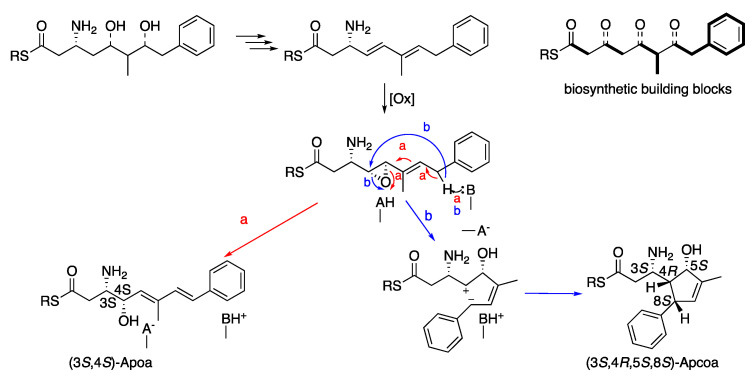
Suggested biosynthetic scheme of the (3*S*,4*S*)-Apoa and (3*S*,4*R*,5*S*,8*S*)-Apcoa units.

Theonellamide J (**1**) presented an identical molecular formula of C_76_H_99_^79^BrN_16_O_27_ and thirty-five degrees of unsaturation, as **4**. Its UV spectrum presented one significant absorption at 212 nm, in contrast to the other theonellamides that presented a significant UV absorption at ~288 nm [14], suggesting the absence of the conjugated diene in **1**. The structure of **1** was solved by analyses of its 1D and 2D NMR spectra in three solvent systems: 4:1 DMSO-*d*_6_:H_2_O at 50 °C (Figures S13–S21 and Table S3), 4:1 DMSO-*d*_6_:D2O at 50 °C (Figures S22–S29 and Table S4), and DMF-*d*_7_ (Figures S30–S37 and Table S5). The presence of a tri-substituted double-bond, in **1**, instead of the conjugated system of the 3-amino-4-hydroxy-6-methyl-8-phenyl-5*E*,7*E*-octadienoic acid (Apoa) in the theonellamides including **2**–**4**, was evident from the NMR data of **1**–**4** (4:1 DMSO-*d*_6_:H_2_O at 50 °C, Table 1 and Tables S3, S6, S7, S2). The structure elucidation of **1** was achieved by analyses of the ^1^H, ^13^C and ^15^N, 1D and 2D (COSY, TOCSY, ROESY, H-C and H-N HSQC, and H-C and H-N HMBC, Figures S13–S21) spectra of **1** in 4:1 DMSO-*d*_6_:H_2_O at 50 °C. Based on these analyses, the following amino acid and sugar derivatives were established and found identical with those of **4**: 2-aminoadipic acid (Aad), asparagine (Asn), 3-methyl-*p*-bromophenylalanine (BrMePhe), histidinoalanyl (sHis-sAla), hydroxyasparagine (Han), *iso*-serine (iSer), phenylalanine (Phe), two serine (Ser) units, threonine (Thr), and galactose (Gal). The structure elucidation of the last amino acid derivative we named 3-amino-5-hydroxy-6-methyl-8-phenyl-4,8-cyclo-6-octenoic acid (Apcoa) was much more challenging and is described in detail below. COSY correlations (Table S3 and Figure S19) of **1** in 4:1 DMSO-*d*_6_:H_2_O at 50 °C afforded three continuous spin systems: (a) H-2a, H-2b, H-3 and 3-NH; (b) H-4, H-5, 6-Me, H-7 and H-8 that closes a ring due to correlation of H-8 with H-4; and (c) H-10,10’, H-11,11’, and H-12. TOCSY correlations in the same solvent system allowed the connection of H-3 with H-4. Correlations from COSY experiments of **1** in 4:1 DMSO-*d*_6_:D_2_O at 50 °C (Table S4 and Figure S27) and in DMF-*d*_7_ (Table S5 and Figure S34) confirmed the connectivity of H-2a and H-2b through H-8 and the closure of a five-membered ring through correlations between H-4 and H-8. Data from the HSQC spectra in the three solvent systems (listed above, Tables S3–S5) established the connectivity between adjacent proton and carbons. HMBC correlations (in 4:1 DMSO-*d*_6_:H_2_O at 50 °C, Table S3 and Figure S16) of H-2b (δ_H_ 1.93) with the carboxyamide carbon at δ_C_ 171.8 (C-1), of H-2a (δ_H_ 2.33) with the nitrogen-bearing carbon at δ_C_ 48.3 (C-3), of H-4 (δ_H_ 1.71) with the oxygen-bearing and quaternary sp^2^ carbons at δ_C_ 78.9 (C-5) and 145.6 (C-9), of H-5 (δ_H_ 3.98) with C-3 and the two sp^2^ carbons at δ_C_ 142.3 (C-6) and 129.1 (C-7), of the proton signal of 6-Me (δ_H_ 1.61) with C-5, C-6, and C-7, of the vinyl proton H-7 (δ_H_ 5.02) with C-4, C-6 and the methine carbon at δ_C_ 50.6 (C-8), of H-8 (δ_H_ 2.76) with the symmetric aromatic carbons at δ_C_ 128.3 (C-10,10’), of the symmetric aromatic protons H-10,10’ (δ_H_ 7.14) with C-8 and C-10’,10, of H-11,11’ (δ_H_ 7.20) with C-9, C-11’,11 and the aromatic carbon at δ_C_ 127.0 (C-12), established the structure of the later moiety as 3-amino-5-hydroxy-6-methyl-8-phenyl-4,8-cyclo-6-octenoyl (Apcoa). The latter assignment was supported by the HMBC correlations (in DMF-*d*_7_, Table S5) of H-2a and 2b (δ_H_ 2.66, 2.20, respectively) with the carboxyamide at δ_C_ 172.5 (C-1) and methine carbon at δ_C_ 48.8 (C-3), of H-4 (δ_H_ 1.96) with the carbons at δ_C_ 79.2, 50.9, and 146.0 (C-5, C-8, and C-9, respectively), of H-5 (δ_H_ 4.30) with the carbons at δ_C_ 152.8 and 128.8 (C-6 and C-7, respectively), of 6-Me (δ_H_ 1.72) with the carbons at δ_C_ 79.2, 142.8, and 128.8 (C-5, C-6, and C-7, respectively), of H-7 (δ_H_ 5.15) with the carbons at δ_C_ 62.4, 79.2, 142.8, 13.4, and 50.9 (C-4, C-5, C-6, 6-Me, and C-8, respectively), of H-8 (δ_H_ 3.06) with carbons at δ_C_ 62.4, 142.8, and 146.0 (C-4, C-6, and C-9, respectively), of H-10,10 (δ_H_ 7.21) with the carbons at δ_C_ 128.6 and 128.2 (C-10’,10 and C-11,11’, respectively) and of H-11,11’ (δ_H_ 7.29) with C-9, C-11’,11, and C-12 (δ_C_ 126.4). The relative configuration of the chiral centers of the cyclopentene ring were established based on the NOE correlations in DMF-*d*_7_ and in 4:1 DMSO-*d*_6_:D_2_O at 50 °C, between H-4 and H-5 and H-8, as 4R*,5S*,8S* (Tables S4 and S5 and Figure 2). In 4:1 DMSO-*d*_6_:D_2_O and 4:1 DMSO-*d*_6_:H_2_O at 50 °C, the signal of H-5 resonates close to the HDO and H_2_O signal and no NOE correlations with it were observed. Taking into consideration the biosynthetic route of Apoa (Figure 3) [21], where the absolute configuration of C-3 in **1** is identical with C-3 of **4** and that the hydroxy group at C-5 of **1** is derived from the same epoxide which leads to the hydroxy group at C-4 of **4**, we suggest that the absolute configuration of the chiral centers of the Apcoa subunit of is 3*S*,4*R*,5*S*,8*S*.

The 5-*cis*-Apoa-theopalauamide (**2**) was isolated as an amorphous solid presenting an HR ESI MS molecular adduct ion at *m*/*z* 1769.5944, [M + Na]^+^, calculated for the molecular formula C_76_H_99_^79^BrN_16_O_27_Na and thirty-five degrees of unsaturation, similar to the data of **1** and **4**. Comparison of the ^1^H and ^13^C NMR spectra of **2** and **4** (in 4:1 DMSO-*d*_6_:H_2_O at 50 °C, Table 1) revealed significant differences only for the trisubstituted diene system of Apoa. The most significance differences were noticed for 6-Me (δ_H_ 1.82 in **2** instead of 1.63 in **4**, δ_C_ 21.1 in **2** instead of 13.7 in **4**) and CH-7 (δ_H_ 7.09 in **2** instead of 6.55 in **4**, δ_C_ 126.5 in **2** instead of 134.0 in **4**), suggesting an isomerization of the 5,6-*E*-double bond in **4** to a *Z*-double bond in **2** [25]. The structure elucidation of **2** was achieved by full analyses of the ^1^H, ^13^C, and ^15^N, 1D and 2D (COSY, TOCSY, ROESY, H-C and H-N HSQC, and H-C and H-N HMBC, Table S6 and Figures S39–S47) spectra of **2** in 4:1 DMSO-*d*_6_:H_2_O at 50 °C. Based on these analyses, the planar structure of **2** was established as 5-*cis*-Apoa-theopalauamide.

Theonellamide K (**3**), isolated as a white solid material, presented an HR ESI MS molecular adduct ion at *m*/*z* 1669.7034, [M + H]^+^, corresponding to the molecular formula C_76_H_101_N_16_O_27_ and thirty-five degrees of unsaturation. The pattern of the molecular ion clearly suggested that **3**, in contrast to the other three compounds (**1**, **2,** and **4**), does not contain a bromine atom in its skeleton. The 1H and ^13^C NMR spectra of **3** was very similar to that of **4** (in 4:1 DMSO-*d*_6_:H_2_O at 50 °C, Table 1, Figures S49, S50 and S3 and S4, respectively). Comparison of the ^1^H and ^13^C NMR spectra of **3** and **4** (Table 1) revealed that the *para*-bromophenyl unit in **4** is substituted by a phenyl unit in **3**. Analyses of the ^1^H, ^13^C and ^15^N, 1D and 2D (COSY, TOCSY, ROESY, H-C and H-N HSQC, and H-C and H-N HMBC, Table S7 and Figures S49–S57) spectra of **3** established the latter unit as 3-MePhe and the planar structure of theonellamide K as **3**.

The absolute configuration of the amino acids comprising **1**–**4** was established using Marfey’s [22] and advanced Marfey’s [23] methods. We prepared the acid hydrolysate of compounds **1**–**4** and of an authentic mixture of theonellamides A, D, and E (kindly given to us by Prof. Shigeki Matsunaga). The acid hydrolysate of the mixture of theonellamides A, D, E, and **1**–**4** was reacted with L-dinitrofluorophenylalanylamide (L-DFPAA) and that of **3** and **4** also with D-DFPAA. Standards of L- and D-Ser, iSer, Thr, *allo*Thr, Phe, Asp, aminoadipice acid (Aad), and *threo*-Has with L-DFPAA, were prepared. We first injected the samples to an analytical RP-18 HPLC column (Marfey’s method) [22] but could not detect all of the amino acids. The absolute configuration of only a few amino acids could be determined by this procedure: L-Phe, L-Ser, L-*allo*Thr, and L-Asn (as L-Asp). We then turned to analyzing the samples by LCMS (advanced Marfey’s method) [23], and the results are summarized in Table 2. Comparison of the retention times and *m*/*z* of the negative and positive molecular ions of the L-DPAA-AA derivatives from the hydrolysate of theonellamides A,D,E mixture with those derived from **1**–**4** (Table 2) allowed the establishment of the absolute configuration of the amino acids for whom we did not have standards: L-sHis-D-sAla and (2*S*,3*S*)-BrMePhe. Furthermore, the comparison allowed the confirmation of the absolute configuration of those for whom we had only few of the possible isomers: (2*S*,3*R*)-Han (as (2*S*,3*R*)-Has), L-iSer (L,D-diastereomer elute earlier than the L,L-diastereomer, as reported by Fukuhara et. al) [17], and L-Aad, and those that were identified by Marfey’s method: L-Phe, L-Ser, L-*allo*Thr, and L-Asn. The advanced Marfey’s method [23] allowed us to determine the absolute configuration of α-carbon of MePhe in **3** as 2*S* based on the earlier elution of L-DPAA-MePhe (56.1 min) over the D-DPAA-MePhe (62.0 min). Assuming that MePhe is the biosynthetic precursor for BrMePhe, we propose that the absolute configuration of MePhe is (2*S*,3*S*), similar to that of BrMePhe. The absolute configuration of the galactose moiety in **1**–**4** was determined by modified (LCMS instead of HPLC detection) “Tanaka’s method” for monosaccharides [24]. The monosaccharides obtained by hydrolysis of the glycopeptide were converted to the thiocarbamoyl–thiazolidine derivatives, and their retention times were as those of D- and L-galactose (Table 3). Using this procedure, the galactose units of **1**–**4** were established to be D-galactose.

The crude extract of *T. swinhoei* presented significant toxicity (at 10 μg/mL) against HTC-116 colon carcinoma, OVCAR-4 ovarian adenocarcinoma, and EKVX lung adenocarcinoma cell lines, but not against OVCAR-7 ovarian carcinoma cell line, and some marginal enhancement of the antibacterial activity of oxacillin *Bacillus subtilis*. The cytotoxicity of pure **1**–**4** was tested against HTC-116 colon carcinoma cell line and IC_50s_ obtained are summarized in Table 4. Comparison of the cytotoxicity of **1**–**4** to the HTC-116 cell line reveal that when comparing the cytotoxicity of **4** with **3**, which vary in BrMePhe versus MePhe, respectively, there is only small decrease in the cytotoxicity of **3** relative to **4**. However, changes to the structure of the Apoa, from 5*E* (in **4**) to 5*Z* (in **2**), show an order of magnitude reduction in the cytotoxicity of **2** compared to **4**, and from a diene (in **4**) to a cyclic structure (in **1**), resulting in practically nonactive **1**.

## 3. Discussion

Most of the theonellamides described to date, theonellamides A–F, H, and I [14,15,17,22], were isolated from *T. swinhoei* samples collected in Japan, while theopalauamide originated from samples collected in Palau and Mozambique [12], and theonegramide [13] derived from a sample collected in the Philippines. Theonellamide G is the only theonellamide described to date from a *T. swinhoei* sample collected in the Red Sea [16]. In the current study, six samples of *T. swinhoei* collected by ROV at different depths, 10, 15, 25, 30, 59, and 65 m, at the northern part of the Gulf of Aqaba near Eilat, during 2017, were studied. LCMS analyses of the extracts of these samples revealed that they contain, along with the known swinholide A, some unknown swinholides and theonellamides. Furthermore, the analyses revealed that they can be divided into two distinct chemotypes, one from shallow water (10, 15, and 30 m) that contains one major component, theopalauamide, much smaller amounts of additional two theonellamides, and traces of potentially other theonellamides, while the second one, that appears in deeper waters (25, 59, and 65 m), contains, in addition to the major theopalauamide, smaller amounts of a large variety of other known and unknown theonellamides (Figure S1 and Table S1). Examination of the LCMS chromatogram of *T. swinhoei* sample #1156, collected in 1982 in the southern part of the Gulf of Aqaba, revealed a much more diverse mixture of theonellamides and lower dominancy of theopalauamide (Figure S2), but as it was derived from an extract of many individual animals, we cannot classify it as a third chemotype. Investigation of the latter sample led to the isolation of the major known cyclic glycopeptide, theopalauamide (**4**), along with three new theonellamides, theonellamide J (**1**), 5-*cis*-Apoa-theopalauamide (**2**), and theonellamide K (**3**). The bioactivity studies of the four cyclic peptides (**1**–**4**) revealed that the nonproteinogenic amino acid, 3-amino-4-hydroxy-6-methyl-8-phenyl-5*E*,7*E*-octadienoic acid (Apoa), is essential for their cytotoxic activity. This study verifies the recent findings, of Mori et. al. [21], that the *Entotheonella* sp. recovered from a sample of the white form *T. swinhoei* collected in the Gulf of Aqaba contains genes responsible for the biosynthesis of both the swinholides and theonellamides. We continue to study the minor theonellamides present in the crude extract of *T. swinhoei* #1156. 

## 4. Materials and Methods

### 4.1. General Experimental Procedures 

Optical rotation values were obtained on a Jasco P-1010 polarimeter at the sodium D line (589 nm). UV spectra were recorded on an Agilent 8453 spectrophotometer (Agilent, Santa Clara, CA, USA). IR spectra were recorded on a Bruker Tensor 27 FT-IR instrument (Bruker, Billerica, MA, USA). NMR spectra were recorded on a Bruker Avance III spectrometer (Bruker, Karlsruhe, Germany) at 500.13 MHz for ^1^H and 125.76 MHz for ^13^C; chemical shifts were referenced to TMS δ_H_ and δ_C_ = 0 ppm. DEPT, COSY-45, gTOCSY, gROESY, gHSQC, gHMQC, and gHMBC spectra were recorded using standard Bruker pulse sequences. Low-resolution mass spectra were recorded on a Waters MaldiSynapt instrument (Waters, Milford, MA, USA) (ESI and APPI) and a Waters Xevo TQD instrument (Waters, Milford, MA, USA) (ESI). High-resolution mass spectra were recorded on a Waters MaldiSynapt instrument (ESI and APPI). HPLC separations were performed on a Merck Hitachi HPLC system (L-6200 Intelligent pump and L-4200 UV-Vis detector, Hitachi, Tokyo, Japan) and an Agilent 1100 Series HPLC system (Agilent, Santa Clara, CA, USA).

### 4.2. Biological Material 

The sponge sample #1156 was collected on February 1982 by scuba diving in the southern end of the Gulf of Aqaba, the Northern Red Sea, at 20 m depth (27°59.02′ N 34°25.32′ E). The sponge was identified as *Theonella swinhoei* (Gray, 1868) by M. Ilan, based on external morphology, types of spicules, and internal skeletal arrangement, fitting the species recorded descriptions [26]. Six samples of *T. swinhoei* were collected from the Gulf of Aqaba by scuba diving and ROV at 10–64 m depth (permits no.: 2017/41753, 2018/42032; Israel Nature and Parks Authority): Sample A, 10 m, sponge volume 70 mL; Sample B, 15 m, sponge volume 95 mL; Sample C, 25 m, sponge volume 50 mL; Sample D, 30 m, sponge volume 250 mL; Sample E, 59 m, 40 mL; Sample F, 64 m; 1.1 L. Each sample was desiccated into 2 cm pieces and frozen immediately at −80 °C. The samples were shipped frozen to Tel Aviv University and lyophilized (Virtis 2k, SP Scientific inc., Warminster, PA, USA) to yield the dry samples: A, 14.9 g; B, 20.4 g; C, 10.8 g; D, 44.9 g; E, 8.0 g; F, 190.0 g. Vouchers were deposited at the Steinhardt Museum of Natural History and National Research Center, Tel Aviv University (M1232, M1233, from 22°36.56′ N 73°38.38′ W; M1236, M1237, from 22°05.28′ N 74°32.15′ W; M1239 from 21°40.40′ N 73°50.31′ W). The cell mass was frozen immediately after collection and then lyophilized.

### 4.3. LCMS Analysis of the Sponge Samples Extracts 

The freeze-dried samples were extracted three times overnight with 9:5:2 ethyl acetate:MeOH:water. The combined extracts were evaporated to dryness and suspended again in water. Each water sample was partitioned with dichloromethane and n-butanol. The samples were dried in vacuo, weighed, and redissolved in water (n-butanol and water samples) or MeCN/DMSO 1:1 (dichloromethane samples) to a concentration of 15 mg/mL (A–D) or 3 mg/mL (E and F). These samples were filtered (0.2 μm) and injected into the LC-MS. The analysis was performed on a Waters Acquity UPLC (Waters, Milford, MA, USA) coupled with a UV detector (Waters Acquity TUV detector) and mass spectrometer (Waters Xevo TQD) on a C18 (1.7 μm, 2.1 Å, ~100 mm) column (Waters). The mobile phase compositions were (A) 95% water/5% MeCN containing 0.1% formic acid and (B) MeCN containing 0.1% formic acid. The elution gradient was as follows: 0.1 min of 100% A, linear gradient to 100% B over 30 min, and then 2 min at 100% B. Samples of 10 μL were injected, and the flow rate was 0.5 mL/min. The UV detector was set to 250 nm, and the mass spectrometer was operated in both negative and positive ion modes, scanning between 100 and 2000 mass units. The interpretation of the data was conducted after the run on both positive and negative ion modes using Waters MassLynx software (v4.1).

### 4.4. Extraction and Isolation 

Lyophilized material (220 gr) of the marine sponge *T. swinhoei* (#1156) was extracted by using four different solvents or solvent mixtures (1:1 hexane: dichloromethane, followed by ethyl acetate, 1:1 ethyl acetate: acetone, and finally 1:1 acetonitrile: water). The extracts were analyzed by proton and carbon NMR together with mass spectroscopy, and the most polar extract, 1:1 MeCN: water (61.83 g), was further separated on reverse-phase flash C-18 column (YMC-GEL,120A) eluted with solvent of decreasing polarity from 100% water to 100% methanol in 10% gradual steps and finally with ethyl acetate, to afford 12 fractions of which fractions 5 and 6 were further separated on Sephadex LH-20 (Merck, Darmstadt, Germany). Fraction 5 (1.82 g) was separated by size exclusion chromatography on Sephadex LH-20 column eluted with 100% water to afford 12 fractions. The fractions were merged together on the basis of ^1^H NMR and mass spectroscopy analyses. Fractions 1–4 (1.07 g), from the latter chromatography, were combined and further separated using a CombiFlash EZ Prep equipped with a reverse-phase C-18 column (Teledyne Isco, 150 gr HP C18Aq, 150 g loaded with 3 gr sample) eluted with linear gradient from 100% water to 100% MeCN within 60 minutes, affording 45 fractions (collected by sample auto-collector) that were combined by proton NMR and MS data. Fractions 27–45 (96 mg) were dissolved in water and separated on a preparative reverse-phase HPLC RP-18 column (YMC-Pack ODS-A, 10 µm, 250 × 20 mm, YMC Co. Kyoto, Japan) eluted with isocratic solvent system, 30:70 acetonitrile: acetic acid 0.5% in water, to yield the known theopalauamide (**4**, 43.0 mg, t_R_ 33.1 min, 0.02% yield), the novel 5-*cis*-Apoa-theopalauamide (**2**, 10.0 mg, t_R_ 33.4 min, 0.004% yield), and semi pure **1**. Semi pure **1** (23.5 mg) was dissolved in water and further separated on preparative reverse-phase HPLC RP-18 column (Phenomenex Synergi, Hydro-RP, 10 µm, 250 × 21.20 mm, Phenomenex Co., Golden, CO, USA) in 67:33 0.1% TFA in water: acetonitrile to afford the novel theonellamide J (**1**, 6.2 mg, t_R_ 72.9 min, 0.003% yield). Fraction 6 (3.74 g) was separated by size-exclusion chromatography on Sephadex LH-20 column eluted with 100% water to afford four fractions. Fractions 3 and 4 were combined (0.36 gr) and further separated on a preparative reverse-phase HPLC RP-18 column (YMC-Pack ODS-A, 10 µm, 250 × 20 mm) eluted with a gradient solvent system from 3:7 to 1:1 MeCN: acetic acid 0.5% in water within 60 min, to yield theonellamide K (**3**, 14.5 mg, t_R_ 32.7 min, 0.007% yield) and theopalauamide (**4**, 82.0 mg, t_R_ 39.6 min, 0.04% yield).

### 4.5. Physical Data of the Compounds

*Theonellamide J (**1**):* Amorphous brownish solid; [α]^25^_D_ 9.8 (*c* 4.0, MeOH); UV (MeOH) λ_max_ (log ε) 212 nm (4.76); IR (ATR Diamond) *ν*_max_ 3294, 3041, 1655, 1548, 1402, 1203, 1024 cm^−1^; ^1^H and ^13^C NMR: see Table 1 and Table S1 in Supporting Information; HRESIMS [M + H]^+^, *m*/*z* 1747.6157 (calcd for C_76_H_100_^79^BrN_16_NO_27_, 1747.6127).

*5-cis-Apoa-theopalauamide (**2**):* White amorphous solid; [α]^25^_D_ 21.5 (*c* 6.6, H_2_O); UV (H_2_O) λ_max_ (log ε) 287 nm (3.88); IR (ATR Diamond) *ν*_max_ 3294, 3000, 1655, 1548, 1402, 1203, 1024, 1001 cm^−1^; ^1^H and ^13^C NMR: see Table 1 and Table S1 in Supporting Information; HRESIMS [M + Na]^+^, *m*/*z* 1769.5944 (calcd for C_76_H_99_^79^BrN_16_NNaO_27_, 1769.5947).

*Theonellamide K (**3**):* Amorphous white solid; [α]^25^_D_ 11.3 (*c* 9.6, H_2_O); UV (H_2_O) λ_max_ (log ε) 288 nm (4.32); IR (ATR Diamond) *ν*_max_ 3303, 3000, 1656, 1549, 1402, 1024 cm^−1^; ^1^H and ^13^C NMR: see Table 1 and Table S1 in Supporting Information; HRESIMS [M + H]^+^, *m*/*z* 1669.7034 (calcd for C_76_H_101_N_16_O_27_, 1669.7022). 

*Theopalauamide (**4**):* Amorphous white solid; [α]^25^_D_ 9.8 (*c* 28, H_2_O), lit. [α]_D_ 19 (*c* 0.4, MeOH) [12]; ^1^H and ^13^C NMR: see Table 1 and Table S2 in Supporting Information; HRESIMS [M − H]^−^, *m*/*z* 1745.5978 (calcd for C_76_H_98_^79^BrN_16_O_27_, 1745.5971).

### 4.6. Determination of the Absolute Configuration of the Amino Acids by Marfey’s Method 

Compounds **1**–**4** and authentic mixture of theonellamides A, D, and E (0.5 mg each) were hydrolyzed in 6 N HCl (1 mL). The reaction mixture was maintained in a sealed glass bomb at 104 °C for 18 h. The acid was removed in vacuo, and the residue was suspended in 250 μL of H_2_O. A solution of 1-fluoro-2,4-dinitrophenyl-5-L-alanine amide (FDAA) in acetone (0.03 M, 20 μL per each amino acid in the peptide) and NaHCO3 (1 M, 20 μL per each amino acid) was added to the reaction vessel. The reaction mixture was stirred at 40 °C for 2.5 h in the dark. HCl (2 M, 10 μL per each amino acid) was added to the reaction vessel, and the solution was evaporated in vacuo. The FDAA–amino acid derivatives from the hydrolysate were dissolved in 1 mL MeCN and compared with standard FDAA–amino acids (prepared by the same procedure) by an HPLC analysis: LiChroCART RP-18 column (5 μm, 250 × 4.6 mm), flow rate 1 mL/min, UV detection at 340 nm, linear gradient elution from 0.1% aq. TFA buffer (pH 3) to 6:4 MeCN: 0.1% aq. TFA buffer (pH 3) within 60 min. The absolute configuration of each amino acid was determined by spiking the derivatized hydrolysates with a D,L-mixture of the standard derivatized amino acids [22]. 

### 4.7. Determination of the Absolute Configuration of the Amino Acids by Advanced Marfey’s Method 

A 0.5 mg portion of compounds **3** and **4** was hydrolyzed as described above, divided into two portions and derivatized, one with L-FDAA and the other with D-FDAA, as described above. The two samples of L- and D-FDAA derivatives were analyzed by ESI LC MS. The analysis was performed on a Waters Acquity UPLC coupled with a UV detector (Waters Acquity TUV detector) and mass spectrometer (Waters Xevo TQD) on a C18 (1.7 μm, 2.1 Å, ~100 mm) column (Waters). The mobile phase compositions were (A) water containing 0.1% formic acid and (B) MeCN containing 0.1% formic acid. The elution gradient was as follows: 1 min of 100% A, linear gradient to 30% B over 60 min, and then recycling by linear gradient to 50% B over 10 min and to 100% A over additional 5 min. Samples of 10 μL were injected, and the flow rate was 0.5 mL/min. The UV detector was set to 340 nm, and the mass spectrometer was operated in both negative and positive ion modes, scanning between 100 and 1000 mass units. The interpretation of the data was conducted after the run using Waters MassLynx software (v4.1) [23].

### 4.8. Tanaka’s Method for Determination of the Absolute Configuration of Monosaccharide

The glycopeptides **1**–**4** (500 µg) in 6 M HCl (200 µL) were hydrolyzed for 3 h at 110 °C in a 5 mL conical reactor. The reaction mixture was neutralized with 9 M NH_4_OH (200 µL) and evaporated by a stream of N_2_ gas. After evaporation of the solvent, a solution of L-cysteine methyl ester hydrochloride in pyridine (2 mg/mL; 100 μL) was added and the solution was heated at 60 °C for 1 h. A solution of phenylisothiocyanate in pyridine (0.5 mg/mL, 100 μL) was added to the reaction mixture and the solution was heated for an additional 1 h at 60 °C. The solvent was evaporated, and the dry product was dissolved in 150 μL methanol. The final solution was filtered and analyzed by LCMS on an RP-18 analytical column (1.7 µm, 2.1 × 100 mm) with gradient elution from MeCN−H_2_O (10:90) to MeCN−H_2_O (14:86) containing 0.1% formic acid for 40 min. L- and D-galactose were treated in the same manner, and their retention times were compared with those of the hydrolyzed peptides [24].

### 4.9. Cytotoxicity Assay 

Evaluation of the cytotoxicity of compounds **1**–**4** was performed in vitro on colon carcinoma cells (cell line 7661 HCT-116, ATCC^®^ CCL-247^™^) using XTT cell viability assay. Colon carcinoma cells were seeded in 384-well black plates (Greiner cat. 781091) at 4000 cells/70 μL/well using EVO200 (TECAN) liquid handler. Culture media was as follows: RPMI 1640 (Gibco, cat. 21875-034; lot. 1894815) with 10% FBS (Gibco cat. 12657-029), 1% MEM NEAA (Gibco, REF 11140-035) 1% sodium pyruvate (Gibco, REF 11360-039) and 1% penicillin–streptomycin–nystatin (Biological Industries, REF 03-032-1B). Following 24 h, the medium was replaced with fresh medium containing compounds at 10 μg/mL (0.1% DMSO), 1 μg/mL (0.01% DMSO) and in a dose–response manner for IC_50_ determination from stock of 10 mg/mL or medium only. Serial dilutions (fourteen) of the compounds were prepared to obtain concentrations ranging from 100 μM to 0.06 μM. Following 24 h of incubation with the inhibitors, XTT viability assay (Biological Industries 20-300-1000) was carried out. Wells containing medium only (without cells) were used as blank. A reaction solution was prepared according to manufacturer′s instruction (0.1 mL activation to 5 mL XTT reagent). Medium was removed and replaced with a mix of medium (40 μL) and XTT solution (20 μL). Following 3 h of incubation at 37 °C, absorbance was measured using an M1000PRO (TECAN) plate reader (reading parameters: temperature: −37 °C; shaking: 15 s; amplitude: 2 mm; mode: orbital; absorbance: measurement: 475 nm; reference: 660 nm; number of flashes: 10). The inhibition concentration 50 (IC_50_) were calculated by AAT Bioquest-calculator: https://www.aatbio.com/tools/ic50-calculator (accessed on 12 December 2021). SEM were calculated with IC_50_ Tool Kit: http://www.ic50.tk/index.html (accessed on 12 December 2021).

## Figures and Tables

**Table 1:** ^1^H (500 MHz), ^13^C (125 MHz), and ^15^N 50 MHz) NMR data of compounds **1**–**4** in DMSO-d_6_:H_2_O (4:1) at 50 °C.

Unit/ Position	Theonellamide J (1)		5-*cis*-Apoa-Theo-palauamide (2)		Theonellamide K (3)		Theopalauamide (4)	
	δ_C/*N*_	δ_H_	δ_C/*N*_	δ_H_	δ_C/*N*_	δ_H_	δ_C/*N*_	δ_H_
Apcoa/Apoa-1	171.8, C		172.6, C		172.2, C		172.6, C	
2a	40.4, CH_2_	2.33, t	37.6, CH_2_	2.36, t	37.4, CH_2_	2.37, t	37.6, CH_2_	2.37, m
2b		1.93, dd		2.15, dd		2.13, dd		2.10, brd
3	48.3, CH	4.22, m	53.2, CH	4.10, m	52.8, CH	4.11, m	53.0, CH	4.11, m
3-*NH*	*122.3*, *NH*	7.82, d	*118.0*, *NH*	7.69, d	*118.4*, *NH*	7.70, d	*117.76*, *NH*	7.72, d
4	62.1, CH	1.71, ddd	67.7, CH	4.51, m	68.5, CH	4.25, m	68.7, CH	4.25, m
5	78.9, CH	3.98, m	130.9, CH	5.05, brd	132.9, CH	5.18, d	132.7, CH	5.14, d
6	142.3, C		135.3, C		135.9, C		136.3, C	
6-Me	14.1, CH_3_	1.61, brs	21.1, CH_3_	1.82, brs	13.4 CH_3_	1.65, s	13.5, CH_3_	1.64, s
7	129.1, CH	5.02, brs	126.5, CH	7.09, d	133.8, CH	6.62, d	134.0, CH	6.58, d
8	50.6, CH	2.76, m	130.8, CH	6.60, d	128.2, CH	6.50, d	128.6, CH	6.49, d
9	145.6, C		138.1, C		137.8, C		138.0, C	
10,10’	128.3, CH	7.14, d	127.6, CH	7.43, d	126.9, CH	7.41, d	127.1, CH	7.38, d
11,11’	129.0 CH	7.20, t	129.7, CH	7.27, dd	129.5, CH	7.31, t	129.7, CH	7.29, t
12	127.0, CH	7.13, m	129.2, CH	7.17, t	128.3, CH	7.19, t	128.9, CH	7.17, m
Ser1-1	172.3, C		172.8, C		172.3, C		172.8, C	
2	56.6, CH	3.71, m	56.8, CH	3.74, m	56.6, CH	3.78, m	56.8, CH	3.76, m
*2-NH*	*119.2*, *NH*	7.69, brs	*114.7*, *NH*	7.77, m	*119.4*, *NH*	7.77, s	*114.9*, *NH*	7.80, brs
3	61.9, CH_2_	3.62, m	61.2, CH_2_	3.62, m	61.0, CH_2_	3.64, m	61.1, CH_2_	3.64, m
sAla-1	169.6, C		169.8, C		169.6, C		169.9, C	
2	51.1, CH	5.04, m	51.4, CH	5.06, m	51.1, CH	5.06, m	51.4, CH	5.07, m
*2-NH*	*107.7*, *NH*	8.23, d	*108.5*, *NH*	8.26, d	*108.1*, *NH*	8.27, d	*108.3*, *NH*	8.28, d
3a	50.5, CH_2_	4.88, d	50.6, CH_2_	4.89, brd	50.5, CH_2_	4.89, d	50.6, CH_2_	4.90, brd
3b		4.22, m		4.20, m		4.22, dd		4.21, m
Asn-1	171.5, C		171.4, C		171.2, C		171.6, C	
2	51.9, CH	4.48, brm	52.2, CH	4.49, m	51.9, CH	4.52, m	52.2, CH	4.50, m
2-NH	*114.6*, *NH*	7.73, d	*119.2*, *NH*	7.78, s	*114.7*, *NH*	7.78, brs	*119.2*, *NH*	7.77, d
3a	37.1, CH_2_	2.45, m	37.4, CH_2_	2.55, dd	37.1, CH_2_	2.56, dd	37.2, CH_2_	2.58, dd
3b		2.24, m		2.30, dd		2.32, dd		2.35, dd
4	172.2, C		172.5, C		172.1, C		172.5, C	
4-NH_2_	*102.7*, *NH*_2_	7.31, brs	*103.9*, *NH*_2_	7.31, brs	*103.5*, *NH*_2_	7.32, brs	*103.8*, *NH*_2_	7.37, brs
		6.62, s		6.73, brs		6.76, brs		6.76, brs
Han-1	171.5, C		171.3, C		170.9, C		171.4, C	
2	54.4, CH	5.39, m	54.7, CH	5.37, t	54.5, CH	5.36, dd	54.7, CH	5.39, t
2-*NH*	*108.7*, *NH*	8.33, d	*108.8*, *NH*	8.30, d	*108.9*, *NH*	8.32, d	*108.7*, *NH*	8.35, d
3	71.9, CH	3.95, m	72.4, CH	3.96, m	72.2, CH	3.97, brd	72.4, CH	3.98 m
3-OH		4.87, m		5.18, d		5.12, brs		5.19, brd
4	174.2, C		174.5, C		174.1, C		174.5, C	
4-*NH_2_*	*102.2*, *NH*_2_	7.14, brs	*102.0*, *NH*_2_	7.04, m	*102.3*, *NH*_2_	7.25, s	*102.1*, *NH*_2_	7.28, m
		7.19, brs		7.25, m		7.07, s		7.05, m
BrMePhe/MePhe-1	171.1, C		172.0, C		171.7, C		172.0, C	
2	59.1, CH	4.58, m	59.2, CH	4.52, m	59.3, CH	4.51, dd	59.3, CH	4.54, m
2-*NH*	*111.5*, *NH*	7.86, d	*113.1*, *NH*	8.17, d	*114.0*, *NH*	8.23, d	*113.1*, *NH*	8.19, d
3	38.9, CH	3.49, m	39.6, CH	3.35, m	40.1, CH	3.31, dq	39.5, CH	3.35, qd
3-Me	17.9, CH_3_	1.10, d	18.0, CH_3_	1.04, d	18.1, CH_3_	1.08, d	18.0, CH_3_	1.05, d
4	141.2, C		141.8, C		142.4, C		141.8, C	
5,5’	131.1, CH	6.99, d	131.1, CH	6.98, d	128.6, CH	7.07, d	131.1, CH	6.99, d
6,6’	131.5, CH	7.28, d	131.7, CH	7.25, d	128.7, CH	7.12, m	131.7, CH	7.26 d
7	120.5, C		120.6, C		127.1, CH	7.11, t	120.6, C	
iSer-1	172.2, C		171.6, C		171.2, C		171.4, C	
2	69.7, CH	4.13, m	69.9, CH	4.08, m	69.7, CH	4.05, brs	69.8, CH	4.09, m
2-OH		5.42, brs		5.03 m				5.03, m
3a	43.9, CH_2_	3.81, m	44.1, CH_2_	3.73, m	44.0, CH_2_	3.69, m	44.0, CH_2_	3.74, m
3b		2.98, brd		3.00, brd		3.00, dd		3.01, brd
3-*NH*	*100.3*, *NH*	7.24, m	*101.0*, *NH*	7.20, m	*100.2*, *NH*	7.19, m	*101.2*, *NH*	7.22, m
Ada-1	173.9, C		175.9, C		175.4, C		176.0, C	
2	52.2, CH	4.08, m	54.7, CH	3.88, m	54.6, CH	3.87, m	54.7, CH	3.91, m
2-*NH*	*110.6*, *NH*	7.49, d	*116.7*, *NH*	7.58, d	*116.5*, *NH*	7.58, d	*116.5*, *NH*	7.59, d
3a	31.2, CH_2_	1.66, m	32.4, CH_2_	1.65, m	32.4, CH_2_	1.66, m	32.4, CH_2_	1.65, m
3b		1.51, m		1.43, m		1.43, m		1.44, m
4a	21.7, CH_2_	1.22, m	22.2, CH_2_	1.11, m	22.2, CH_2_	1.19, m	22.2, CH_2_	1.12, m
4b		0.88, m						
5a	35.0, CH_2_	2.08, m	35.9, CH_2_	2.05, dt	35.8, CH_2_	2.05, dt	35.9, CH_2_	2.06, m
5b		1.80, m		1.70, m		1.72, m		1.70, m
6	173.4, C		174.3, C		174.1, C		174.3, C	
sHis-1	170.6, C		171.0, C		170.7, C		171.0, C	
2	54.4, CH	4.73, m	54.5, CH	4.74, ddd	54.3, CH	4.77, m	54.6, CH	4.75, dt
2-*NH*	*119.6*, *NH*	8.36, d	*119.6*, *NH*	8.32, d	*119.9*, *NH*	8.37, d	*119.7*, *NH*	8.33, m
3a	26.4, CH_2_	3.28, brt	26.4, CH_2_	3.26, t	26.2, CH_2_	3.28, t	26.4, CH_2_	3.27, t
3b		2.94, brd		2.96, m		2.96, dd		2.98, m
4	131.9, C		132.0, C		131.8, C		132.0, C	
5-*N*	*181.0*, *N*		*181.2*, *N*		*181.4*, *N*		*180.9*, *N*	
6	137.3, CH	8.89, s	137.4, CH	8.85, s	137.2, CH	8.87, s	137.4, CH	8.86, s
7-*N*	*167.4*, *N*		*167.4*, *N*		*167.8*, *N*		*167.9*, *N*	
8	124.0, CH	7.19, s	124.3, CH	7.21, brs	124.1, CH	7.25, s	124.3, CH	7.22, brs
Thr-1	172.5, C		172.7, C		172.5, C		172.7, C	
2	58.7, CH	4.29, m	58.9, CH	4.23, m	58.7, CH	4.22, m	58.9, CH	4.23, m
2-*NH*	*113.5*, *NH*	7.60, d	*114.1*, *NH*	7.67, d	*114.1*, *NH*	7.71, d	*114.3*, *NH*	7.69, d
3	68.8, CH	3.63, m	68.9, CH	3.59, m	68.7, CH	3.61, m	69.0, CH	3.60, m
3-OH		5.40, brs		5.35, d		5.20, d		4.90, m
4	21.6, CH_3_	1.04, d	21.5, CH_3_	0.97, d	21.5, CH_3_	0.97, d	21.6, CH_3_	0.98, d
Ser2-1	169.8, C		169.9, C		169.7, C		169.9, C	
2	56.5, CH	4.46, m	56.8, CH	4.41, m	56.4, CH	4.44, dt	56.6, CH	4.45, m
2*-NH*	*116.7*, *NH*	8.52, d	*116.6*, *NH*	8.47, d	*116.7*, *NH*	8.48, d	*117.0*, *NH*	8.48, d
3	62.0, CH_2_	3.68, m	62.1, CH_2_	3.57, m	61.9, CH_2_	3.64, m	62.1, CH_2_	3.64, m
				3.64, m				
Phe-1	171.5, C		171.8, C		171.4, C		171.6, C	
2	55.2, CH	4.56, m	55.1, CH	4.47, m	54.9, CH	4.55, ddd	55.0, CH	4.56, m
2-*NH*	*115.8*, *NH*	7.97, d	*115.3*, *NH*	7.91, d	*115.7*, *NH*	7.94, d	*115.9*, *NH*	7.96, d
3a	39.2, CH_2_	2.77, m	39.2, CH_2_	2.70, dd	39.2, CH_2_	2.81, dd	39.4, CH_2_	2.81, dd
3b		2.72, m		2.61, dd		2.65, dd		2.65, dd
4	136.9, C		137.3, C		137.3, C		137.3, C	
5,5’	129.6, CH	7.10, m	129.9, CH	7.01, d	129.8, CH	7.14, m	130.0, CH	7.13, d
6,6’	128.9, CH	7.14, m	129.1, CH	7.16, t	129.0, CH	7.22, d	129.2, CH	7.21, m
7	127.3, CH	7.13, m	127.5, CH	7.10, t	127.3, CH	7.18, m	127.7, CH	7.14, m
Gal-1	88.9, CH	5.03, d	89.0, CH	5.02, d	88.9, CH	5.02, d	89.0, CH	5.03, d
2	69.5, CH	3.61, m	69.8, CH	3.62, m	69.7, CH	3.63, m	69.9, CH	3.63, m
3	73.7, CH	3.41, brd	73.8, CH	3.41, m	73.7, CH	3.40, brd	73.9, CH	3.43, brd
4	69.7, CH	3.83, m	69.6, CH	3.81, m	69.5, CH	3.81, m	69.6, CH	3.83, m
5	78.9, CH	3.66, m	79.0, CH	3.64, m	78.8, CH	3.64, m	79.0, CH	3.64, m
6a	61.9, CH_2_	3.65, m	62.1, CH_2_	3.75, m	61.9, CH_2_	3.75, m	62.0, CH_2_	3.75, m
6b		3.58, m		3.54, m		3.64, m		3.64, m

**Table 2:** Retention times of amino acids–DPAA derivative from LCMS.

AA Derivative	Neg/pos ESI Molecular Ion *m*/*z*’s	R_t_ of L-DPAA-AA of TA ^a^	R_t_ of L-DPAA-AA of 1	R_t_ of L-DPAA-AA of 2	R_t_ of L-DPAA-AA of 3	R_t_ of D-DPAA-AA of 3	R_t_ of L-DPAA-AA of 4	R_t_ of D-DPAA-AA of 4
L-sHis-D-sAla ^b^	493/495	12.2	12.4	12.4	12.4	7.9	12.3	7.9
L-sHis-D-sAla ^b^	493/495	13.1	13.3	13.1	13.4	11.6	13.3	nd
L-Ser	356/358	24.8	25.1	24.9	24.9	25.8	24.7	25.7
L-iSer	356/358	27.0	27.3	27.2	27.2	26.4	27.1	26.3
L-*allo*Thr	370/372	27.1	27.3	27.2	27.2	30.0	27.1	30.1
L-Asp	384/386	28.7	29.1	28.7	28.9	31.8	28.8	31.9
L-(*2S*,3*R*)-Has	400/402	30.2	30.7	30.0	30.1	28.5	nd	28.8
L-Aad	412/414	nd ^d^	33.8	33.8	33.7	37.3	33.7	37.4
Apoa/Apcoa	512/514	nd	nd	nd	nd	nd	nd	nd
L-sHis-D-sAla ^c^	745/747	42.5	42.4	42.3	42.5	40.0	42.3	39.7
L-Phe	416/418	51.7	51.8	51.6	51.7	57.8	51.6	57.9
(2*S*,3*S*)-MePhe	430/432	-	--	--	56.1	62.0	-	-
(2*S*,3*S*)-BrMePhe	508,510/510,512	64.6	64.7	64.6	-	-	64.5	66.9

^a^ Theonellamides A,D,E mixture. ^b^ The two isomeric mono-DPAA derivatives. ^c^ Di-DPAA derivative. ^d^ Not detected.

**Table 3:** LCMS-retention times of the monosaccharides thiocarbamoyl–thiazolidine derivatives.

Compound	Mass Weight ESI (Positive)	Retention Time (min)	Absolute Configuration
D-galactose	433	20.09	D-galactose
L-galactose	433	21.79	L-galactose
**1**	433	19.94	D-galactose
**2**	433	19.99	D-galactose
**3**	433	20.08	D-galactose
**4**	433	20.04	D-galactose

**Table 4:** Cytotoxicity data of **1**–**4** against HCT-116 colon carcinoma cell line.

Compound	1	2	3	4	Cytochalasin D
IC_50_ (SEM) in μM	>100	21.8 (±0.7)	3.5 (±0.2)	2.8 (±0.9)	0.8 (±0.03)

## Supplementary Materials

The following are available online at https://www.mdpi.com/article/10.3390/md20010031/s1, Figures S1–S62 of the LCMS chromatograms of *T. swinhoei* extracts. 1D and 2D NMR spectra and HRESIMS spectra of compounds **1**–**4**. Tables S1–S8 with the LCMS peaks annotation and NMR data of compounds **1**–**4**. Cytotoxicity data and inhibition curves of compounds **1**–**4**.
