# Supplementary material for: Theonellamides J and K and 5-cis-Apoa-theopalauamide, Bicyclic Glycopeptides of the Red Sea Sponge Theonella swinhoei
Source: Mar Drugs. 2021 Dec 27;20(1):31. doi: 10.3390/md20010031 (PMC8779245; doi:10.3390/md20010031)

Theonellamides J and K and 5-*cis*-Apoa-theopalauamide, Bicyclic Glycopeptides of the Red Sea Sponge *Theonella swinhoei*

Ohad Hasin [1], Shani Shoahm [2], Yoel Kashman [1], Micha Ilan [2], Shmuel Carmeli [1]*

[1] Raymond and Beverly Sackler School of Chemistry and Faculty of Exact Sciences, Tel Aviv University, Ramat-Aviv 6997801, Israel; ohadhasin@gmail.com, Kashman@post.tau.ac.il.

[2] School of Zoology, George S. Wise Faculty of Life Sciences, Tel Aviv University, Ramat Aviv 6997801, Israel; shanisho@yahoo.com, milan@tauex.tau.ac.il.

* Correspondence: carmeli@tauex.tau.ac.il; Tel.: +972-54-3117290, +972-3-6408550.

Supplementary Material

Table of Content:

S1 Figure S1 LCMS chromatogram of *T. swinhoei* samples collected near Eilat at depts of 10 to 64 meters.
S2 Table S1. Molecular peaks (negative-ESI) and partial identifications of the theonellamides in the chromatograms.
S3 Figure S2 LCMS chromatogram of *T. swinhoei* samples collected in the southern part of the Gulf of Aqaba during the 1980's.
S4 Figure S3. ^{1}H NMR spectrum (500 MHz) of theopalauamide (**4**) in 4:1 DMSO-d_6:H_2O at 50 ^{o}C
S5 Figure S4. ^{13}C NMR spectrum (125 MHz) of theopalauamide (**4**) in 4:1 DMSO-d_6:H_2O at 50 ^{o}C
S6 Figure S5. HSQC spectrum of theopalauamide (**4**) in 4:1 DMSO-d_6:H_2O at 50 ^{o}C
S7 Figure S6. HMBC spectrum of theopalauamide (**4**) in 4:1 DMSO-d_6:H_2O at 50 ^{o}C
S8 Figure S7. COSY spectrum of theopalauamide (**4**) in 4:1 DMSO-d_6:H_2O at 50 ^{o}C
S9 Figure S8. TOCSY spectrum of theopalauamide (**4**) in 4:1 DMSO-d_6:H_2O at 50 ^{o}C
S10 Figure S9. ROESY spectrum of theopalauamide (**4**) in 4:1 DMSO-d_6:H_2O at 50 ^{o}C
S11 Figure S10. N-H HSQC spectrum of theopalauamide (**4**) in 4:1 DMSO-d_6:H_2O at 50 ^{o}C
S12 Figure S11. N-H HMBC spectrum of theopalauamide (**4**) in 4:1 DMSO-d_6:H_2O at 50 ^{o}C
S13 Table S2. NMR data of theopalauamide (**4**) in 4:1 DMSO-d_6:H_2O at 50 ^{o}C
S14 Figure S12. (+)-HRESIMS data of theopalauamide (**4**)
S15 Figure S13. ^{1}H NMR spectrum (500 MHz) of theonellamide J (**1**) in 4:1 DMSO-d_6:H_2O at 50 ^{o}C
S16 Figure S14. ^{13}C NMR spectrum (125 MHz) of theonellamide J (**1**) in 4:1 DMSO-d_6:H_2O at 50 ^{o}C
S17 Figure S15. HSQC spectrum of theonellamide J (**1**) in 4:1 DMSO-d_6:H_2O at 50 ^{o}C
S18 Figure S16. HMBC spectrum of theonellamide J (**1**) in 4:1 DMSO-d_6:H_2O at 50 ^{o}C
S19 Figure S17. COSY spectrum of theonellamide J (**1**) in 4:1 DMSO-d_6:H_2O at 50 ^{o}C
S20 Figure S18. TOCSY spectrum of theonellamide J (**1**) in 4:1 DMSO-d_6:H_2O at 50 ^{o}C
S21 Figure S19. ROESY spectrum of theonellamide J (**1**) in 4:1 DMSO-d_6:H_2O at 50 ^{o}C
S22 Figure S20. N-H HSQC spectrum theonellamide J (**1**) in 4:1 DMSO-d_6:H_2O at 50 ^{o}C
S23 Figure S21. N-H HMBC spectrum of theonellamide J (**1**) in 4:1 DMSO-d_6:H_2O at 50 ^{o}C
S24 Table S3. NMR data of theonellamide J (**1**) in 4:1 DMSO-d_6:H_2O at 50 ^{o}C
S25 Figure S22. ^{1}H NMR spectrum (500 MHz) of theonellamide J (**1**) after dissolving in 4:1 DMSO-d_6:D_2O at 50 ^{o}C

S26 Figure S23. ^{1}H NMR spectrum (500 MHz) of theonellamide J (**1**) 24 h after dissolving in 4:1 DMSO-d_6:D_2O at 50 ^oC
S27 Figure S24. ^{13}C NMR spectrum (125 MHz) of theonellamide J (**1**) in 4:1 DMSO-d_6:D_2O at 50 ^oC
S28 Figure S25. HSQC spectrum of theonellamide J (**1**) in 4:1 DMSO-d_6:D_2O at 50 ^oC
S29 Figure S26. HMBC spectrum of theonellamide J (**1**) in 4:1 DMSO-d_6:D_2O at 50 ^oC
S30 Figure S27. COSY spectrum of theonellamide J (**1**) in 4:1 DMSO-d_6:D_2O at 50 ^oC
S31 Figure S28. TOCSY spectrum of theonellamide J (**1**) in 4:1 DMSO-d_6:D_2O at 50 ^oC
S32 Figure S29. ROESY spectrum of theonellamide J (**1**) in 4:1 DMSO-d_6:D_2O at 50 ^oC
S33 Table S4. NMR data of theonellamide J (**1**) in 4:1 DMSO-d_6:D_2O at 50 ^oC
S34 Figure S30. ^{1}H NMR spectrum (500 MHz) of theonellamide J (**1**) in DMF-d_7
S35 Figure S31. ^{13}C NMR spectrum (125 MHz) of theonellamide J (**1**) in DMF-d_7
S36 Figure S32. HSQC spectrum of theonellamide J (**1**) in DMF-d_7
S37 Figure S33. HMBC spectrum of theonellamide J (**1**) in DMF-d_7
S38 Figure S34. COSY spectrum of theonellamide J (**1**) in DMF-d_7
S39 Figure S35. TOCSY spectrum of theonellamide J (**1**) in DMF-d_7
S40 Figure S36. ROESY spectrum of theonellamide J (**1**) in DMF-d_7
S41 Figure S37. N-H HSQC spectrum theonellamide J (**1**) in DMF-d_7
S42 Table S5. NMR data of theonellamide J (**1**) in DMF-d_7[a]
S43 Figure S38. (+)-HRESIMS data of theonellamide J (**1**)
S44 Figure S39. ^{1}H NMR spectrum (500 MHz) of 5-*cis*-Apoa-theopalauamide (**2**) in 4:1 DMSO-d_6:H_2O at 50 ^oC
S45 Figure S40. ^{13}C NMR spectrum (125 MHz) of 5-*cis*-Apoa-theopalauamide (**2**) in 4:1 DMSO-d_6:H_2O at 50 ^oC
S46 Figure S41. HSQC spectrum of 5-*cis*-Apoa-theopalauamide (**2**) in 4:1 DMSO-d_6:H_2O at 50 ^oC
S47 Figure S42. HMBC spectrum of 5-*cis*-Apoa-theopalauamide (**2**) in 4:1 DMSO-d_6:H_2O at 50 ^oC
S48 Figure S43. COSY spectrum of 5-*cis*-Apoa-theopalauamide (**2**) in 4:1 DMSO-d_6:H_2O at 50 ^oC
S49 Figure S44. TOCSY spectrum of 5-*cis*-Apoa-theopalauamide (**2**) in 4:1 DMSO-d_6:H_2O at 50 ^oC
S50 Figure S45. ROESY spectrum of 5-*cis*-Apoa-theopalauamide (**2**) in 4:1 DMSO-d_6:H_2O at 50 ^oC
S51 Figure S46. N-H HSQC spectrum of 5-*cis*-Apoa-theopalauamide (**2**) in 4:1 DMSO-d_6:H_2O at 50 ^oC

S52 Figure S47. N-H HMBC spectrum of 5-*cis*-Apoa-theopalauamide (**2**) in 4:1 DMSO-d_6:H_2O at 50 ^oC
S53 Table S6. NMR data of theopalauamide (**2**) in 4:1 DMSO-d_6:H_2O at 50 ^oC
S54 Figure S48. (+)-HRESIMS data of 5-*cis*-Apoa-theopalauamide (**2**)
S55 Figure S49. ^1H NMR spectrum (500 MHz) of theonellamide K (**3**) in 4:1 DMSO-d_6:H_2O at 50 ^oC
S56 Figure S50. ^{13}C NMR spectrum (125 MHz) of theonellamide K (**3**) in 4:1 DMSO-d_6:H_2O at 50 ^oC
S57 Figure S51. HSQC spectrum of theonellamide K (**3**) in 4:1 DMSO-d_6:H_2O at 50 ^oC
S58 Figure S52. HMBC spectrum of theonellamide K (**3**) in 4:1 DMSO-d_6:H_2O at 50 ^oC
S59 Figure S53. COSY spectrum of theonellamide K (**3**) in 4:1 DMSO-d_6:H_2O at 50 ^oC
S60 Figure S54. TOCSY spectrum of theonellamide K (**3**) in 4:1 DMSO-d_6:H_2O at 50 ^oC
S61 Figure S55. ROESY spectrum of theonellamide K (**3**) in 4:1 DMSO-d_6:H_2O at 50 ^oC
S62 Figure S56. N-H HSQC spectrum theonellamide K (**3**) in 4:1 DMSO-d_6:H_2O at 50 ^oC
S63 Figure S57. N-H HMBC spectrum of theonellamide K (**3**) in 4:1 DMSO-d_6:H_2O at 50 ^oC
S64 Table S7. NMR data of theonellamide K (**3**) in 4:1 DMSO-d_6:H_2O at 50 ^oC
S65 Figure S58. (+)-HRESIMS data of theonellamide K (**3**)
S66 Table S8. Calculated survival percentage compare to DMSO and IC_{50} of HCT-116 Colon Carcinoma cell line by compounds **1**-**4**
S67 Figure S59. Inhibition curve of HCT-116 Colon Carcinoma cell line by 5-*cis*-Apoa-theopalauamide (**2**)
S68 Figure S60. Inhibition curve of HCT-116 Colon Carcinoma cell line by theonellamide K (**3**)
S69 Figure S61. Inhibition curve of HCT-116 Colon Carcinoma cell line by theopalauamide (**4**)
S70 Figure S62. Under water photograph of *Theonella swinhoei*

S1 Figure S1 LCMS chromatogram of *T. swinhoei* samples collected near Eilat at depts of 10 to 64 meters.

10 m sample

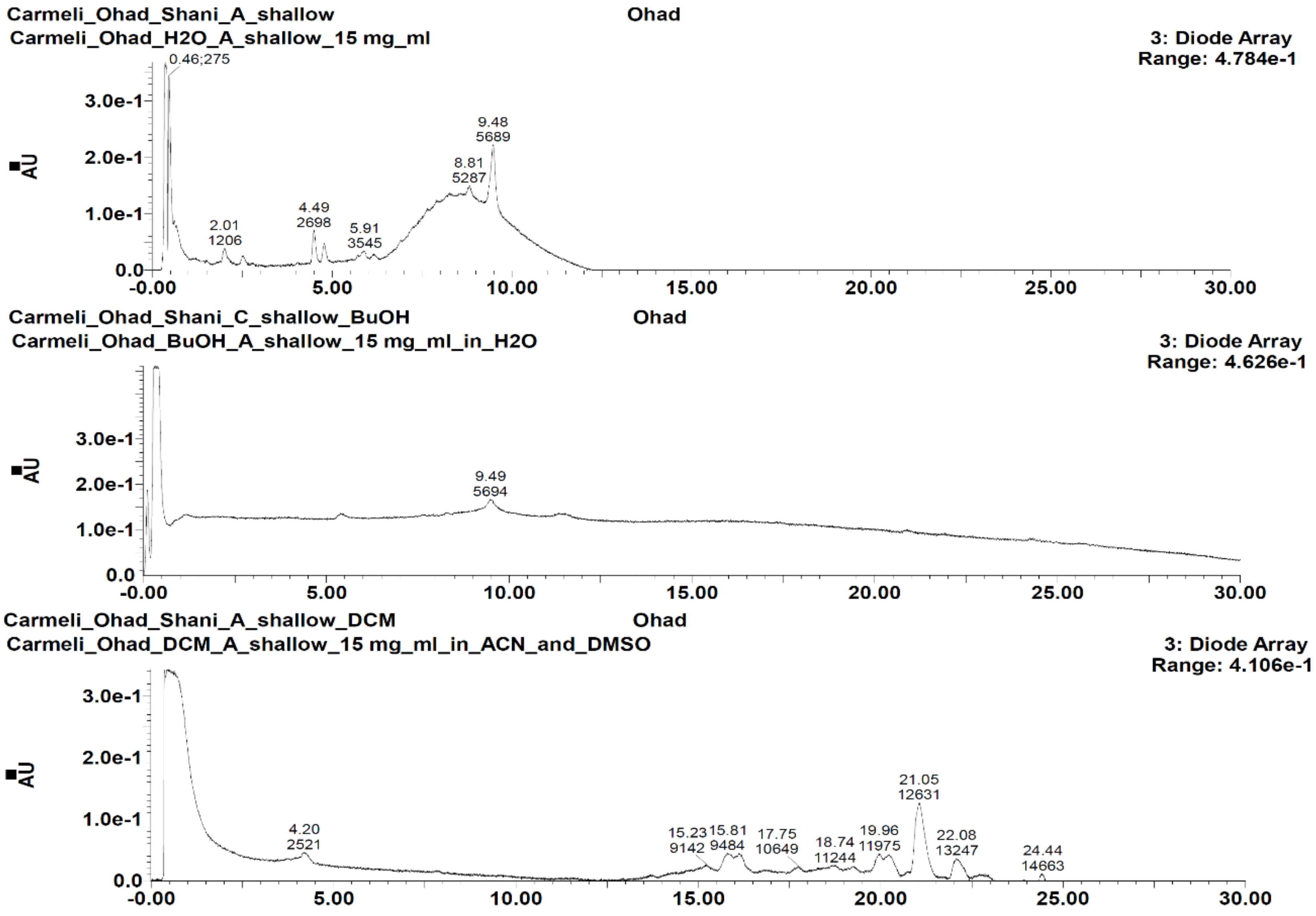

15 m sample

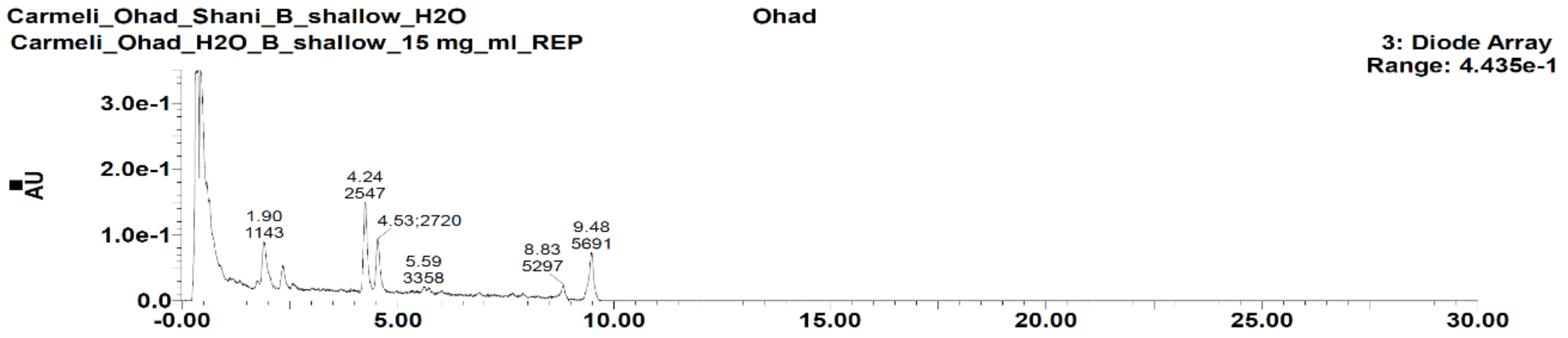
Carmeli_Ohad_Shani_B_shallow_H2O
Ohad
Carmeli_Ohad_H2O_B_shallow_15 mg_ml_REP
3: Diode Array
Range: 4.435e-1
AU
3.0e-1
2.0e-1
1.0e-1
0.0
1.90
1143
4.24
2547
4.53;2720
5.59
3358
8.83
5297
9.48
5691
-0.00
5.00
10.00
15.00
20.00
25.00
30.00

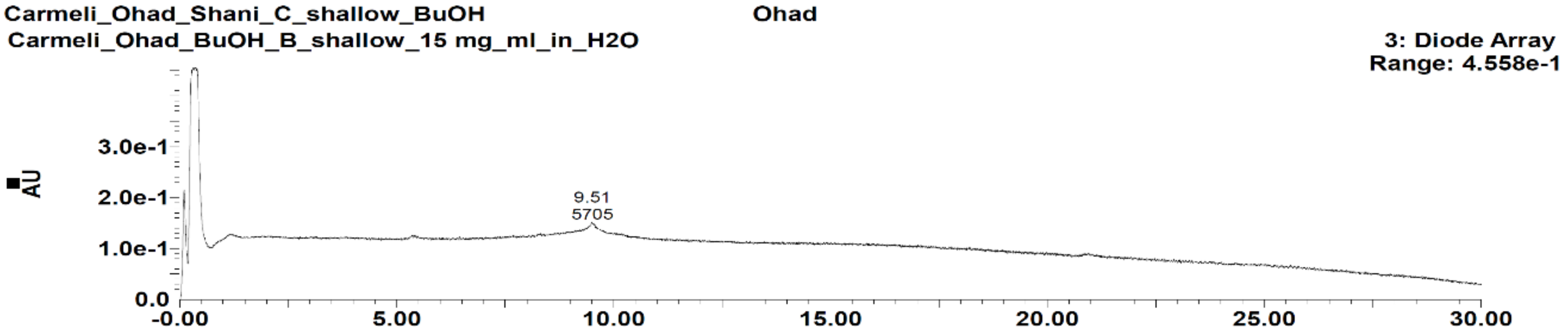
Carmeli_Ohad_Shani_C_shallow_BuOH
Ohad
Carmeli_Ohad_BuOH_B_shallow_15 mg_ml_in_H2O
3: Diode Array
Range: 4.558e-1
AU
3.0e-1
2.0e-1
1.0e-1
0.0
9.51
5705
-0.00
5.00
10.00
15.00
20.00
25.00
30.00

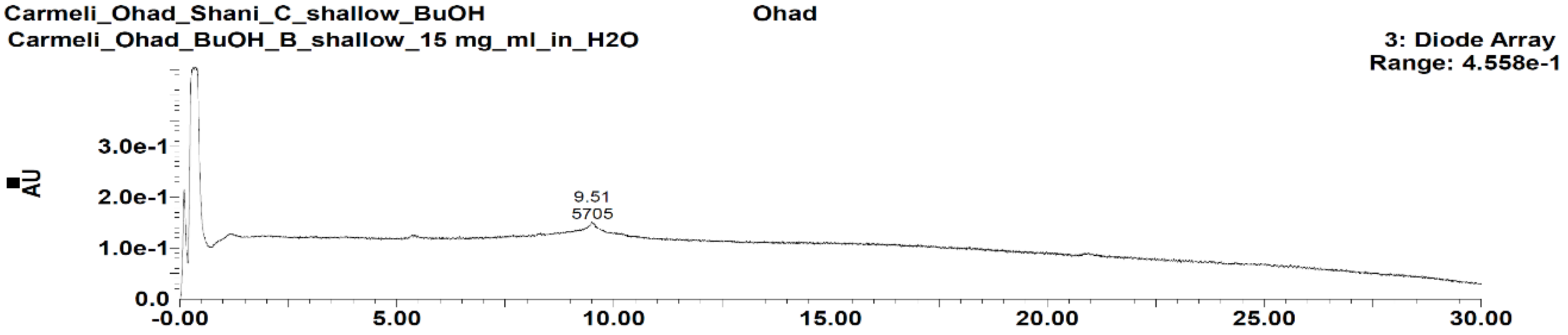
Carmeli_Ohad_Shani_C_shallow_BuOH
Ohad
Carmeli_Ohad_BuOH_B_shallow_15 mg_ml_in_H2O
3: Diode Array
Range: 4.558e-1
AU
3.0e-1
2.0e-1
1.0e-1
0.0
9.51
5705
-0.00
5.00
10.00
15.00
20.00
25.00
30.00

25 m sample

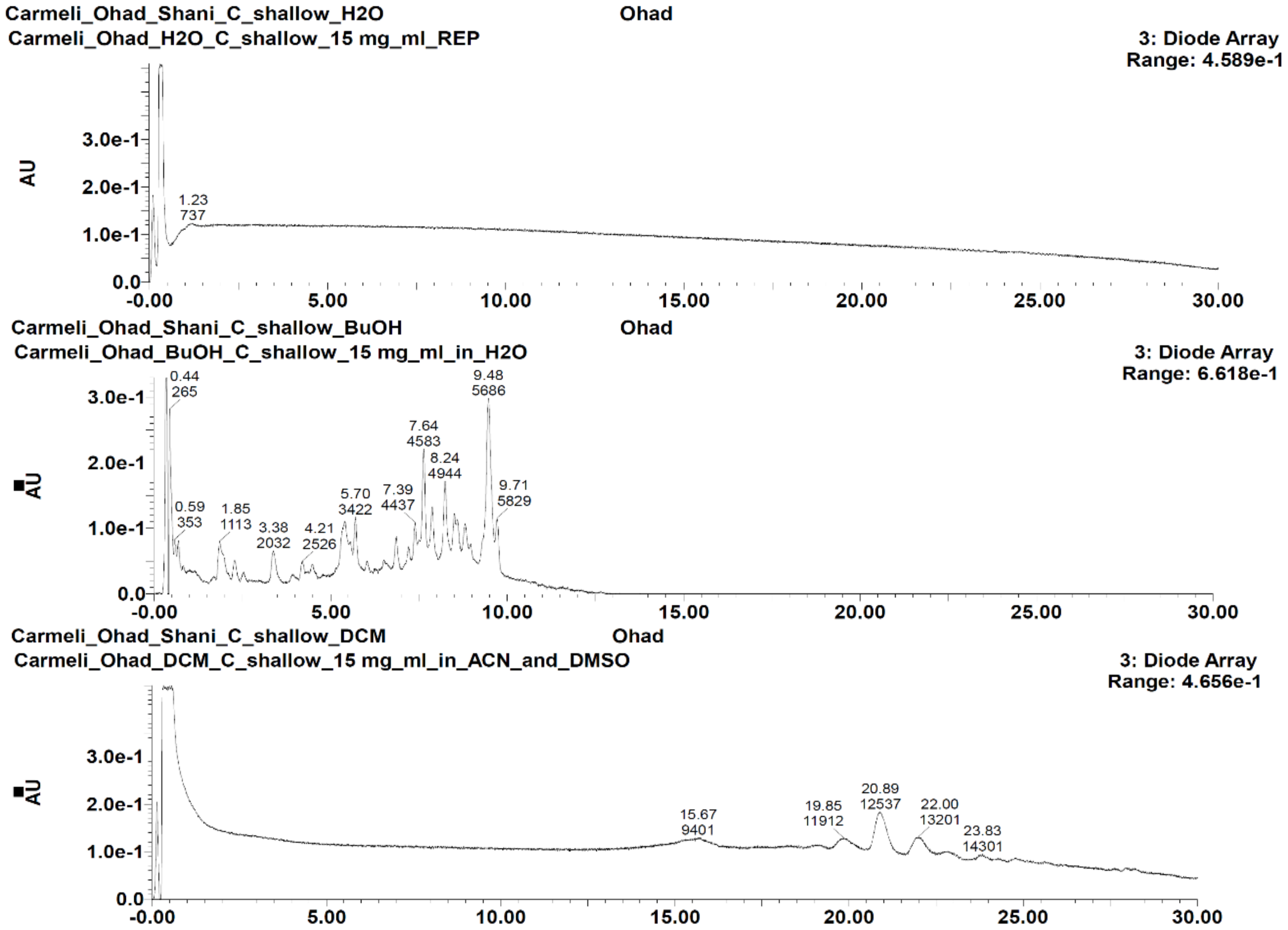
Carmeli_Ohad_Shani_C_shallow_H2O
Ohad
Carmeli_Ohad_H2O_C_shallow_15 mg_ml_REP
3: Diode Array
Range: 4.589e-1
AU
1.23
737
Carmeli_Ohad_Shani_C_shallow_BuOH
Ohad
Carmeli_Ohad_BuOH_C_shallow_15 mg_ml_in_H2O
3: Diode Array
Range: 6.618e-1
AU
0.44
265
0.59
353
1.85
1113
3.38
2032
4.21
2526
5.70
3422
7.39
4437
7.64
4583
8.24
4944
9.48
5686
9.71
5829
Carmeli_Ohad_Shani_C_shallow_DCM
Ohad
Carmeli_Ohad_DCM_C_shallow_15 mg_ml_in_ACN_and_DMSO
3: Diode Array
Range: 4.656e-1
AU
15.67
9401
19.85
11912
20.89
12537
22.00
13201
23.83
14301

30m sample

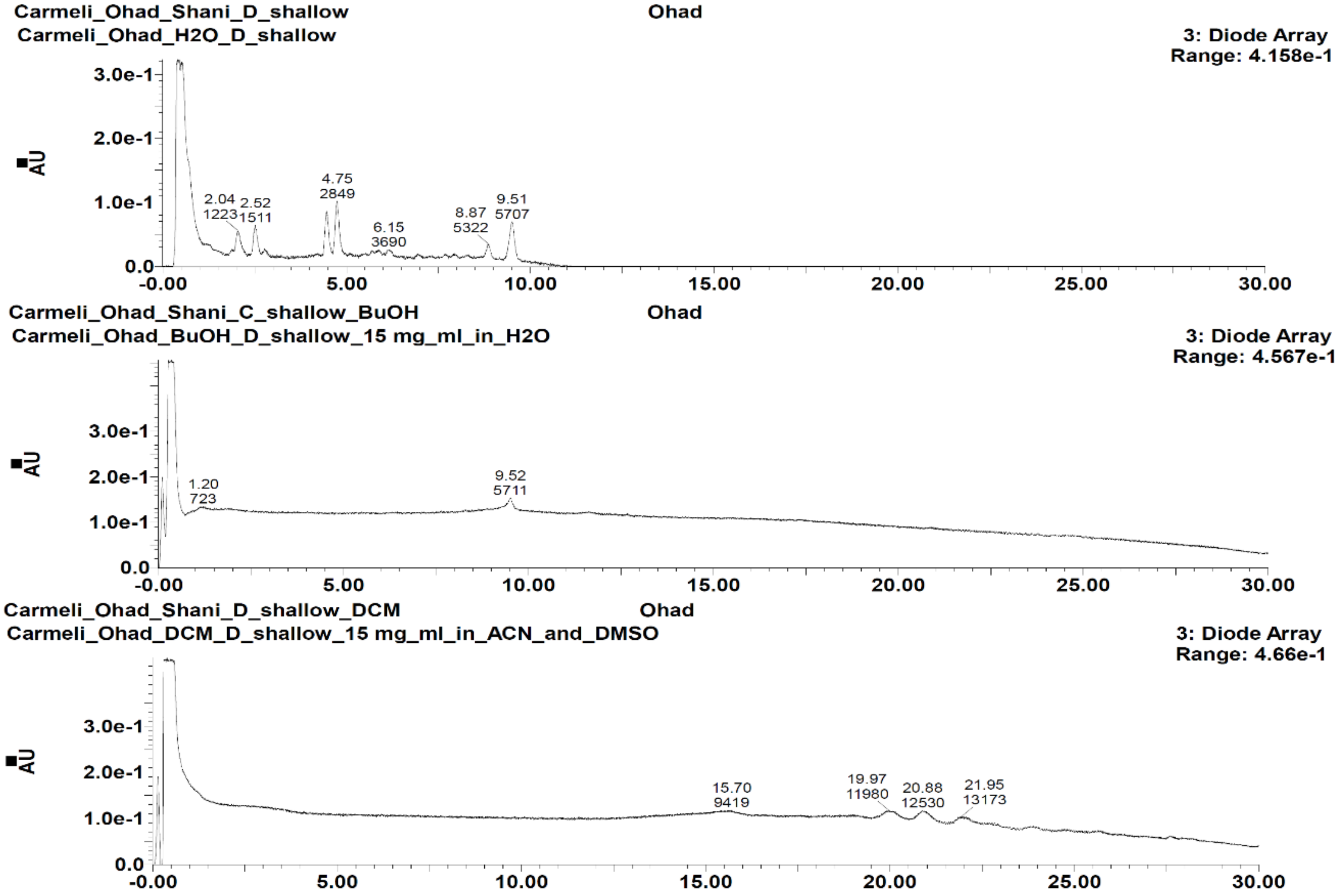
Carmeli_Ohad_Shani_D_shallow
Ohad
Carmeli_Ohad_H2O_D_shallow
3: Diode Array
Range: 4.158e-1
AU
3.0e-1
2.0e-1
1.0e-1
0.0
2.04
1223
2.52
1511
4.75
2849
6.15
3690
8.87
5322
9.51
5707
-0.00
5.00
10.00
15.00
20.00
25.00
30.00
Carmeli_Ohad_Shani_C_shallow_BuOH
Ohad
Carmeli_Ohad_BuOH_D_shallow_15 mg_ml_in_H2O
3: Diode Array
Range: 4.567e-1
AU
3.0e-1
2.0e-1
1.0e-1
0.0
1.20
723
9.52
5711
-0.00
5.00
10.00
15.00
20.00
25.00
30.00
Carmeli_Ohad_Shani_D_shallow_DCM
Ohad
Carmeli_Ohad_DCM_D_shallow_15 mg_ml_in_ACN_and_DMSO
3: Diode Array
Range: 4.66e-1
AU
3.0e-1
2.0e-1
1.0e-1
0.0
15.70
9419
19.97
11980
20.88
12530
21.95
13173
-0.00
5.00
10.00
15.00
20.00
25.00
30.00

59 m sample

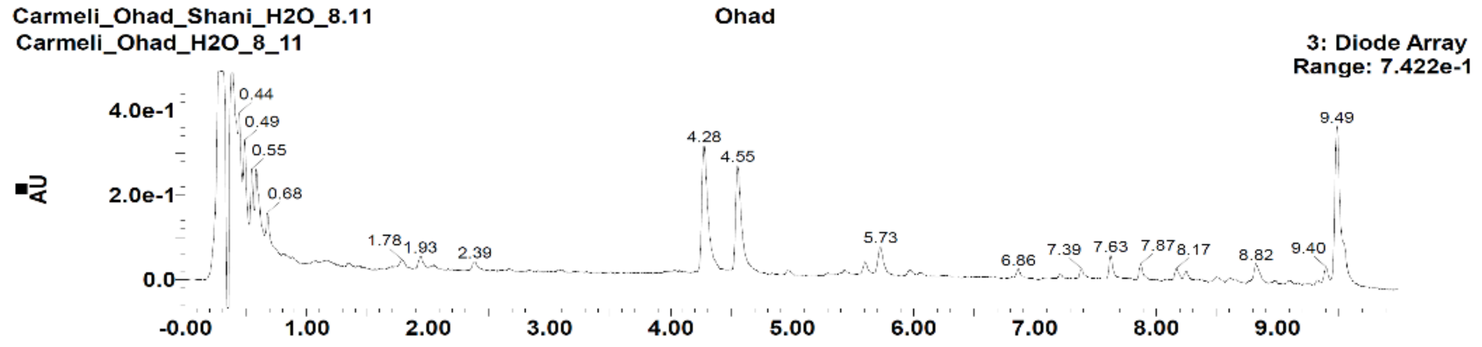
Carmeli_Ohad_Shani_H2O_8.11
Carmeli_Ohad_H2O_8_11
Ohad
3: Diode Array
Range: 7.422e-1
AU
4.0e-1
2.0e-1
0.0
0.44
0.49
0.55
0.68
1.78
1.93
2.39
4.28
4.55
5.73
6.86
7.39
7.63
7.87
8.17
8.82
9.40
9.49
-0.00
1.00
2.00
3.00
4.00
5.00
6.00
7.00
8.00
9.00

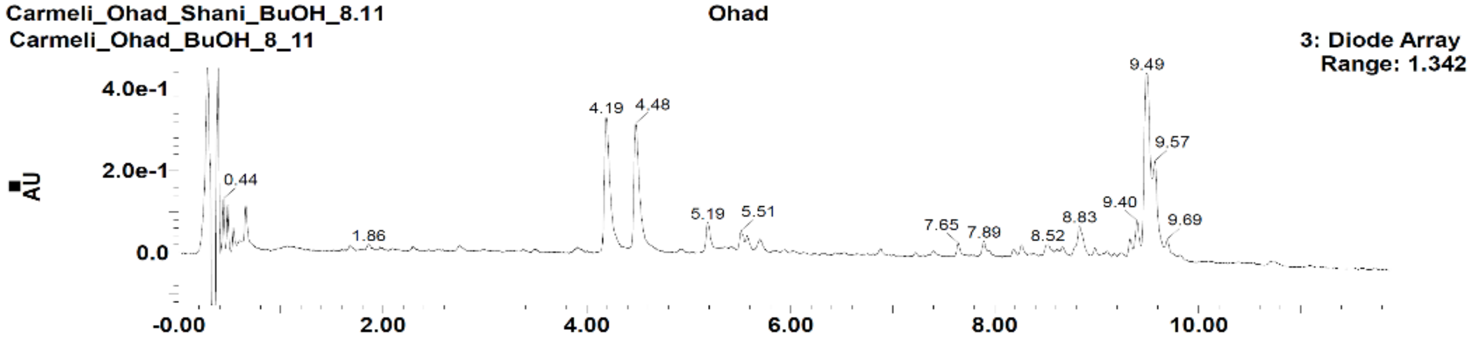
Carmeli_Ohad_Shani_BuOH_8.11
Carmeli_Ohad_BuOH_8_11
Ohad
3: Diode Array
Range: 1.342
AU
4.0e-1
2.0e-1
0.0
0.44
1.86
4.19
4.48
5.19
5.51
7.65
7.89
8.52
8.83
9.40
9.49
9.57
9.69
-0.00
2.00
4.00
6.00
8.00
10.00

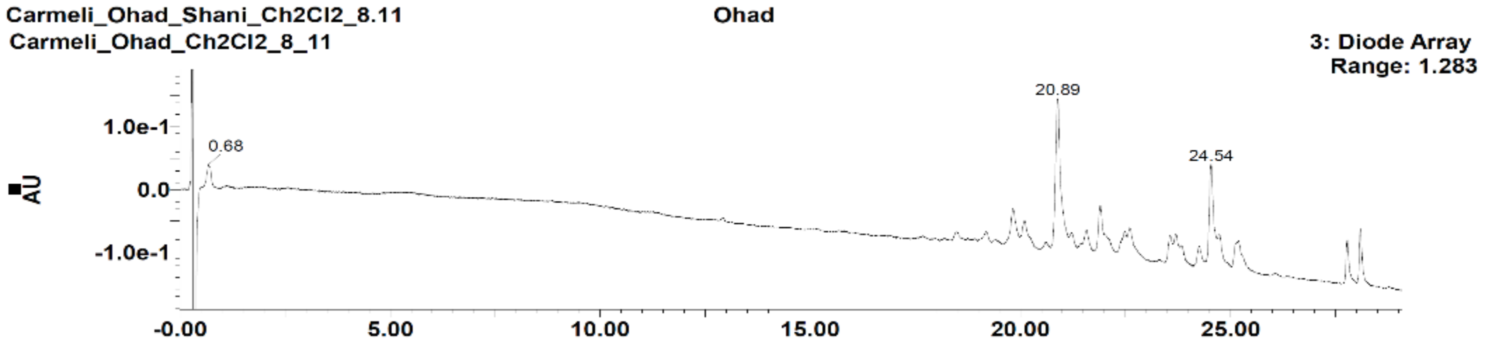
Carmeli_Ohad_Shani_Ch2Cl2_8.11
Carmeli_Ohad_Ch2Cl2_8_11
Ohad
3: Diode Array
Range: 1.283
AU
1.0e-1
0.0
-1.0e-1
0.68
20.89
24.54
-0.00
5.00
10.00
15.00
20.00
25.00

64 m sample

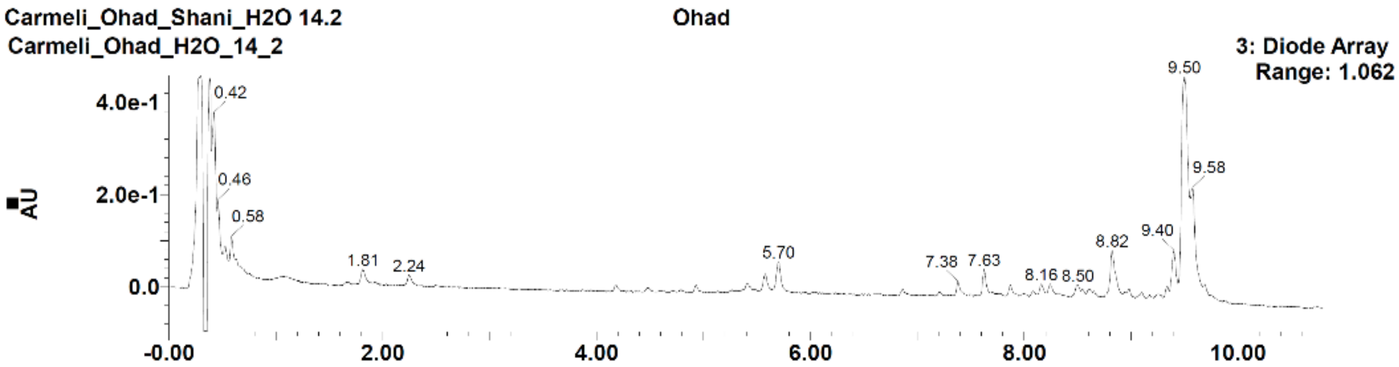
Carmeli_Ohad_Shani_H2O 14.2
Carmeli_Ohad_H2O_14_2
Ohad
3: Diode Array
Range: 1.062
AU
4.0e-1
2.0e-1
0.0
-0.00
2.00
4.00
6.00
8.00
10.00
0.42
0.46
0.58
1.81
2.24
5.70
7.38
7.63
8.16
8.50
8.82
9.40
9.50
9.58

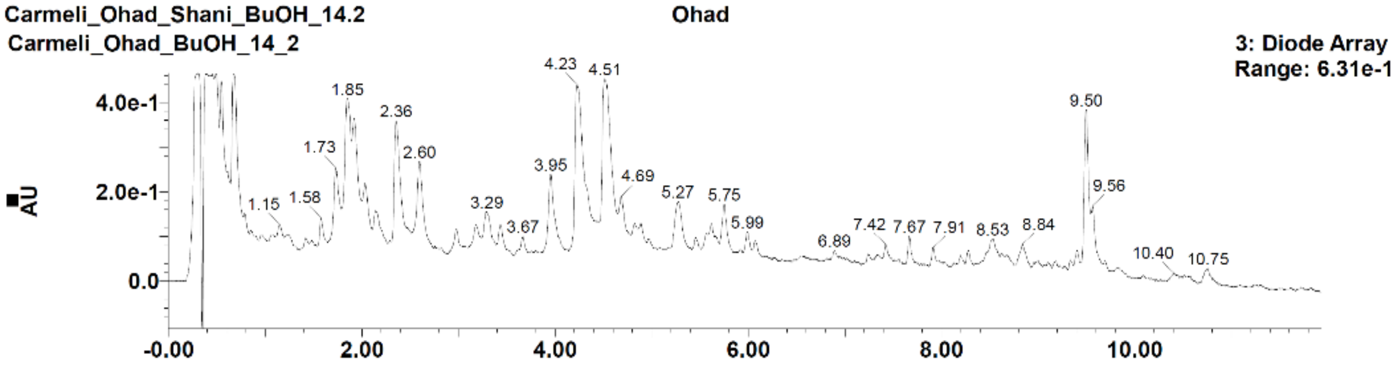
Carmeli_Ohad_Shani_BuOH_14.2
Carmeli_Ohad_BuOH_14_2
Ohad
3: Diode Array
Range: 6.31e-1
AU
4.0e-1
2.0e-1
0.0
-0.00
2.00
4.00
6.00
8.00
10.00
1.15
1.58
1.73
1.85
2.36
2.60
3.29
3.67
3.95
4.23
4.51
4.69
5.27
5.75
5.99
6.89
7.42
7.67
7.91
8.53
8.84
9.50
9.56
10.40
10.75

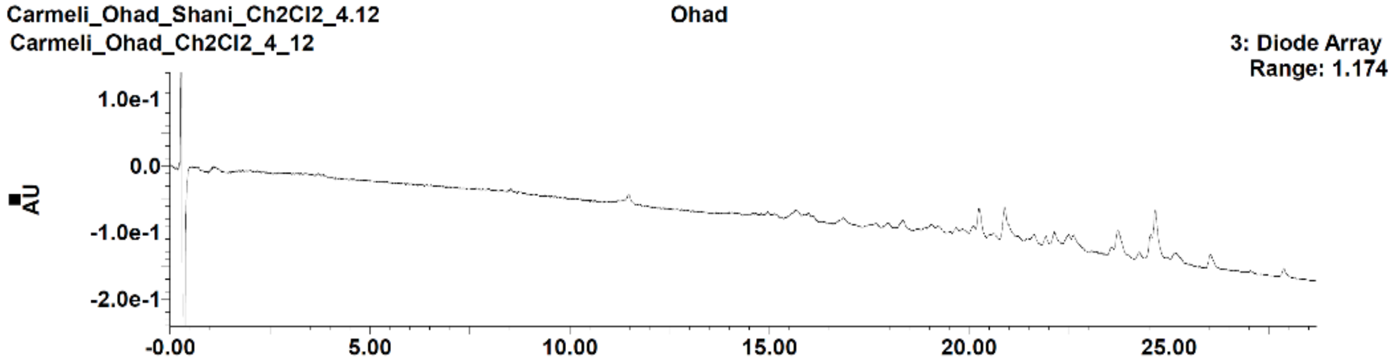
Carmeli_Ohad_Shani_Ch2Cl2_4.12
Carmeli_Ohad_Ch2Cl2_4_12
Ohad
3: Diode Array
Range: 1.174
AU
1.0e-1
0.0
-1.0e-1
-2.0e-1
-0.00
5.00
10.00
15.00
20.00
25.00

S2 Table S1. Molecular peaks (negative-ESI) and partial identifications of the theonellamides in the chromatograms.

t_R min	10 m sample *m/z* (id)	15 m sample *m/z* (id)	25 m sample *m/z* (id)	30 m sample *m/z* (id)	59 m sample *m/z* (id)	64 m sample *m/z* (id)
9.7			1778, 1602		1778, 1602	1584, 1602
9.5	1746 (Tpl)	1746 (Tpl)	1746 (Tpl)	1746 (Tpl)	1746 (Tpl)	1746 (Tpl)
9.4					1716, 1730	1716, 1730
9.0			1762 (TnlA)			
8.9	1668 (TnlK)	1668 (TnlK)	1668 (TnlK)	1668 (TnlK)	1668 (TnlK)	1668 (TnlK)
8.7			1780 (TnlD*)		1778	
8.6			1780 (TnlD*)			
8.3			1780 (TnlD*)		1780 (TnlD*)	1780 (TnlD*)
8.2					1794	1794
8.0			1780 (TnlD*)		1780 (TnlD*)	1780 (TnlD*)
7.7			1780 (TnlD*), 1808,		1778, 1808	1780 (TnlD*), 1808
7.5			1808		1808, 1602	1808, 1602
7.3			1780 (TnlD*)			1780 (TnlD*)
6.9			1702, 1730		1780 (TnlD*)	1780 (TnlD*)
			1780 (TnlD*)			
6.6			1670			
6.1	1602		1602, 1678	1602		
5.8		1602, 1618	1618		1602, 1618	1602, 1618
5.6			1620, 1602, 1590		1602	1602
5.5			1620, 1602			

Tpl – theopalauamide; TnlA – theonellamide A; TnlK – theonellamide K, this study; TnlD* - theonellamide D or isomer of it.

S3 Figure S2 LCMS chromatogram of *T. swinhoei* samples collected in the southern part of the Gulf of Aqaba during the 1980’s.

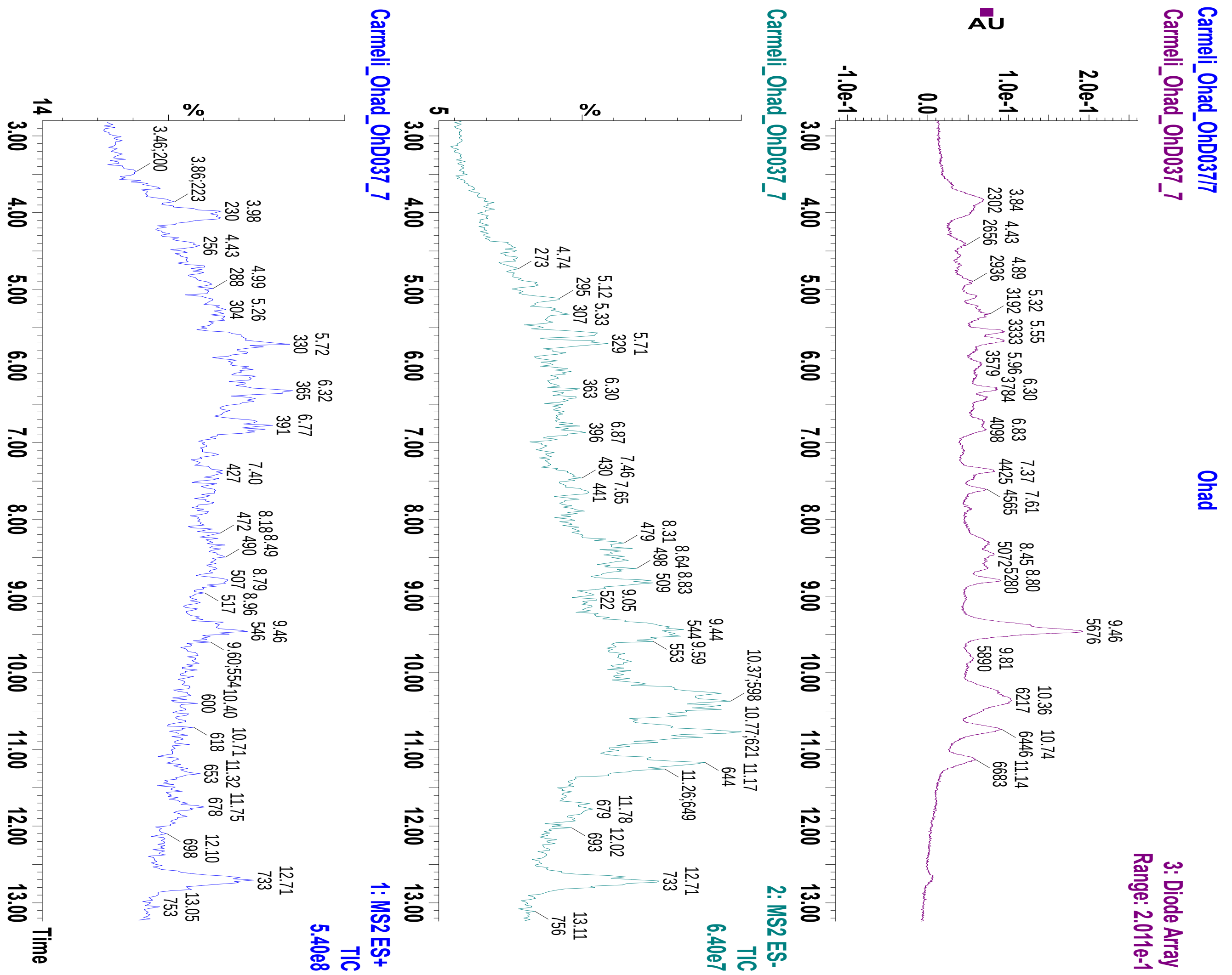

Figure S3. ^{1}H NMR spectrum (500 MHz) of theopalauamide (**4**) in 4:1 DMSO-d_6:H_2O at 50 ^{o}C

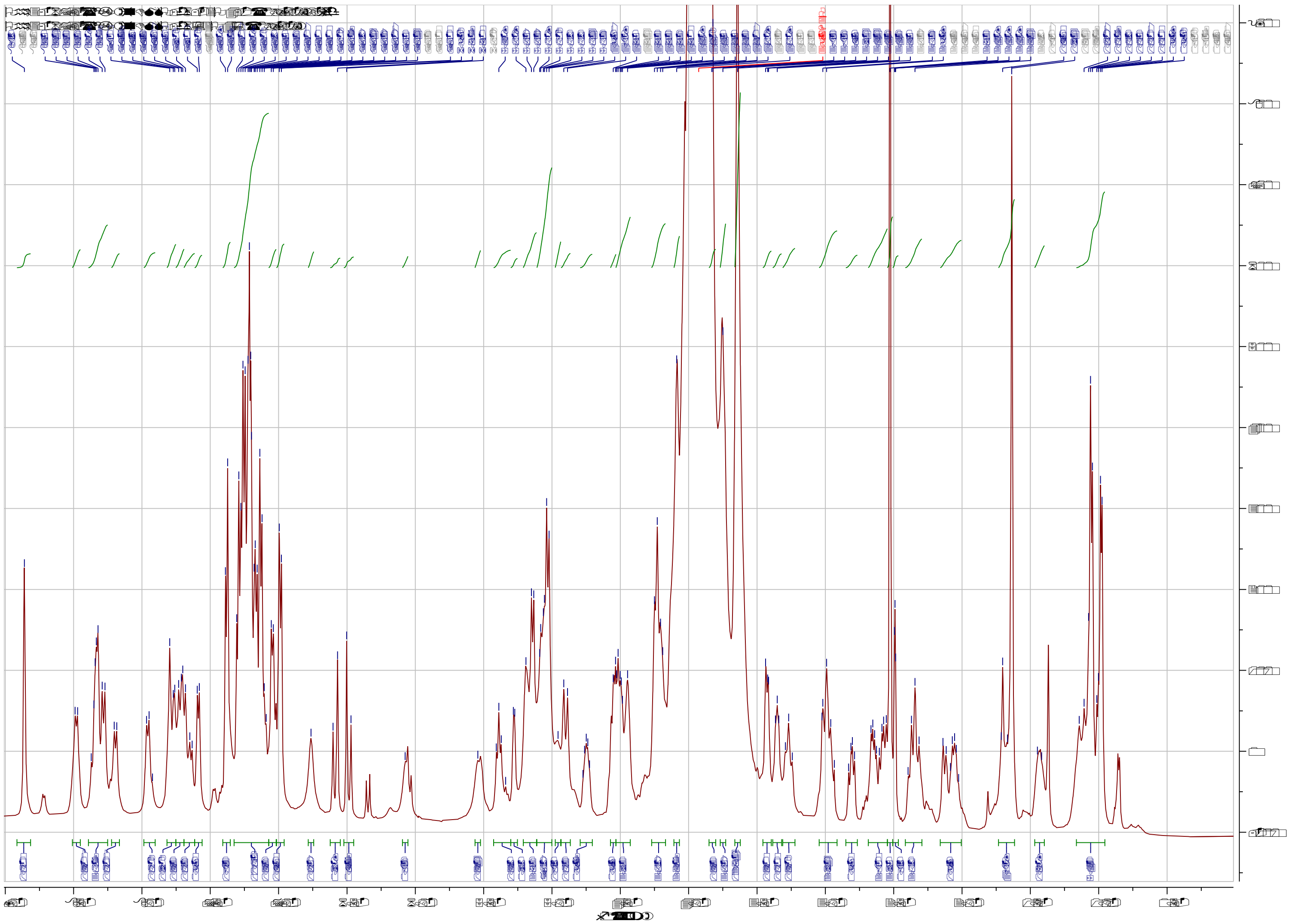

Figure S4. ^{13}C NMR spectrum (125 MHz) of theopalauamide (**4**) in 4:1 DMSO-d_6:H_2O at 50 ^{o}C

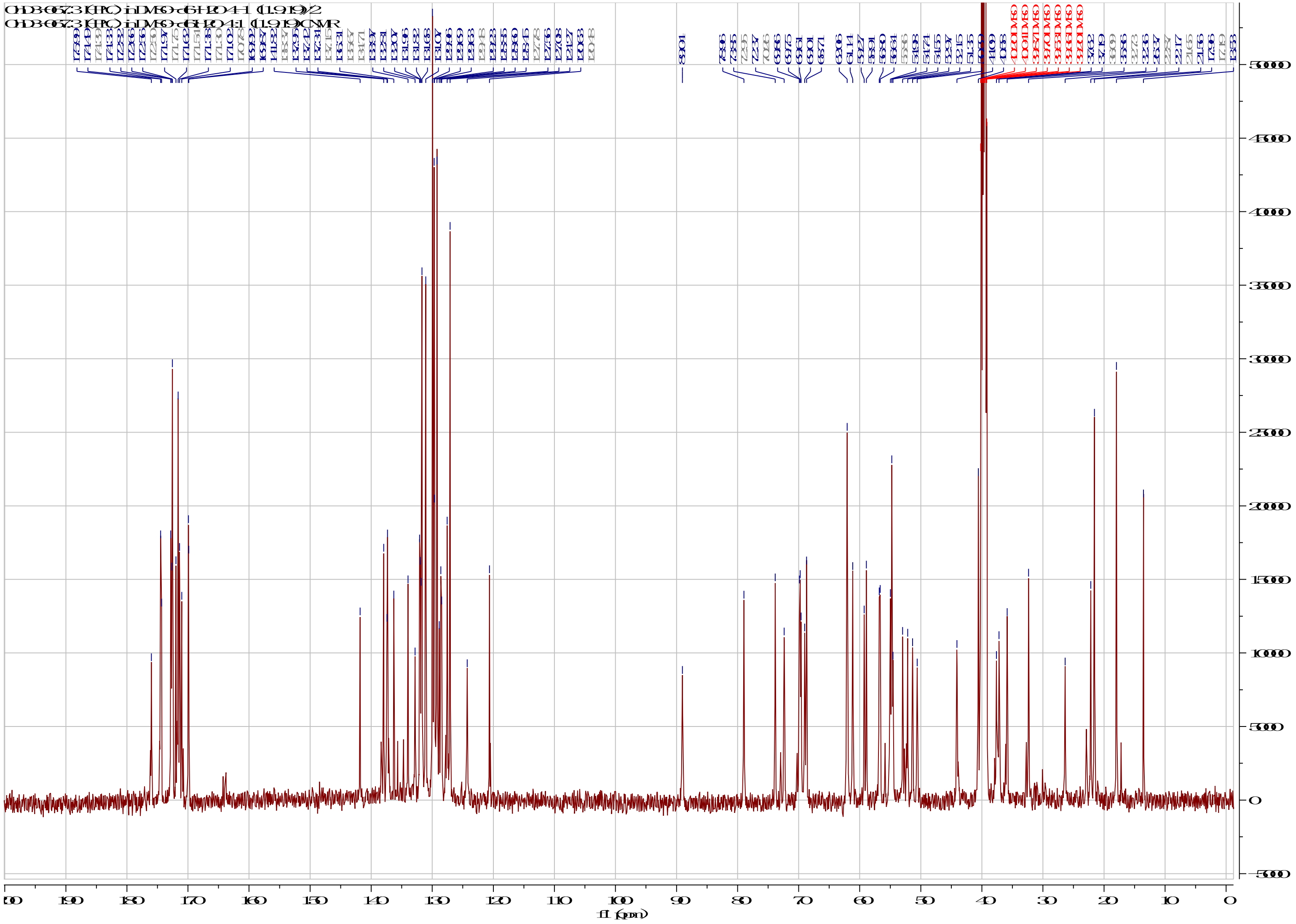

Figure S5. HSQC spectrum of theopalauamide (**4**) in 4:1 DMSO-d_6:H_2O at 50 ^oC

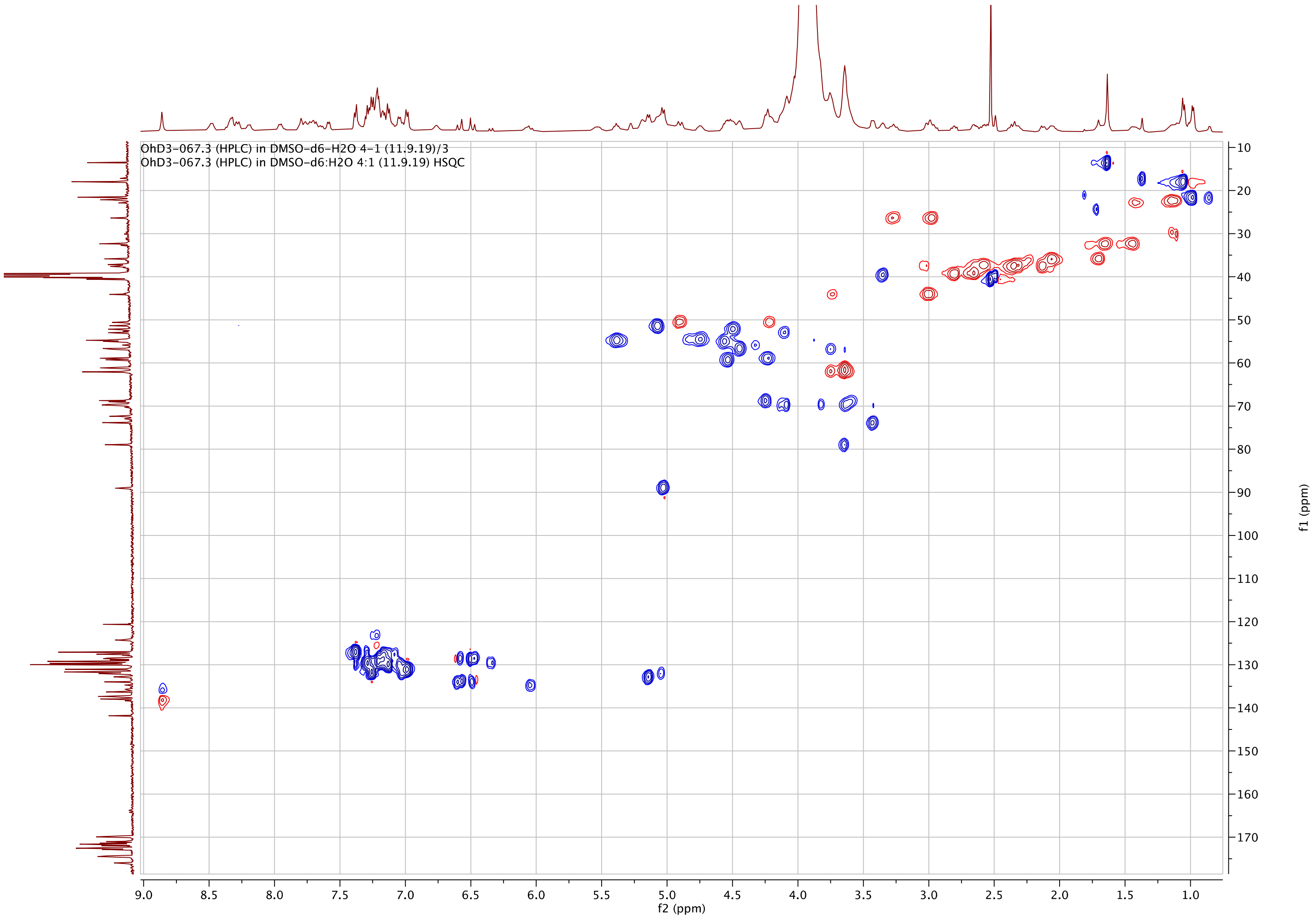

Figure S6. HMBC spectrum of theopalauamide (**4**) in 4:1 DMSO-d_6:H_2O at 50 °C

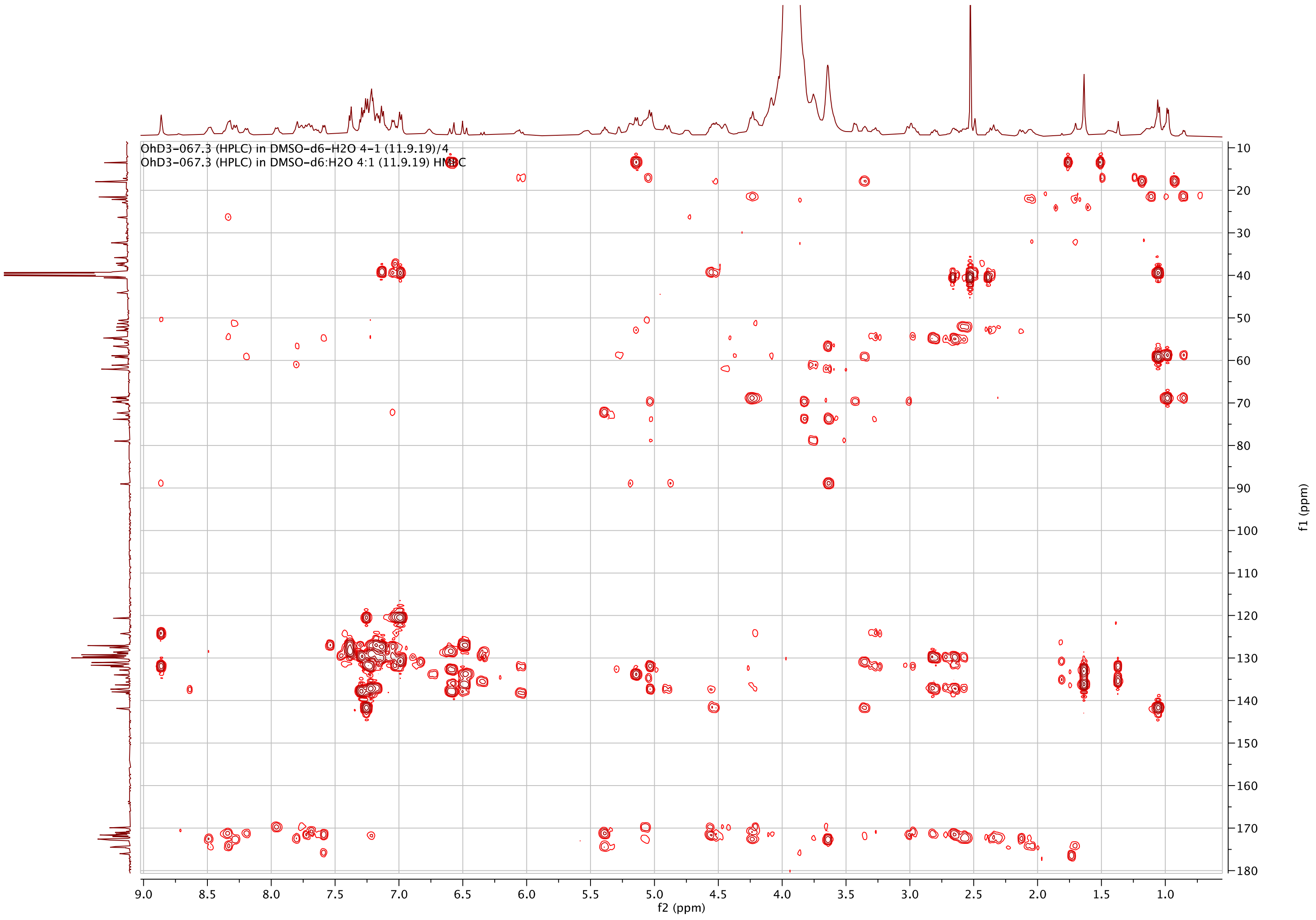

Figure S7. COSY spectrum of theopalauamide (**4**) in 4:1 DMSO-d_6:H_2O at 50 ^oC

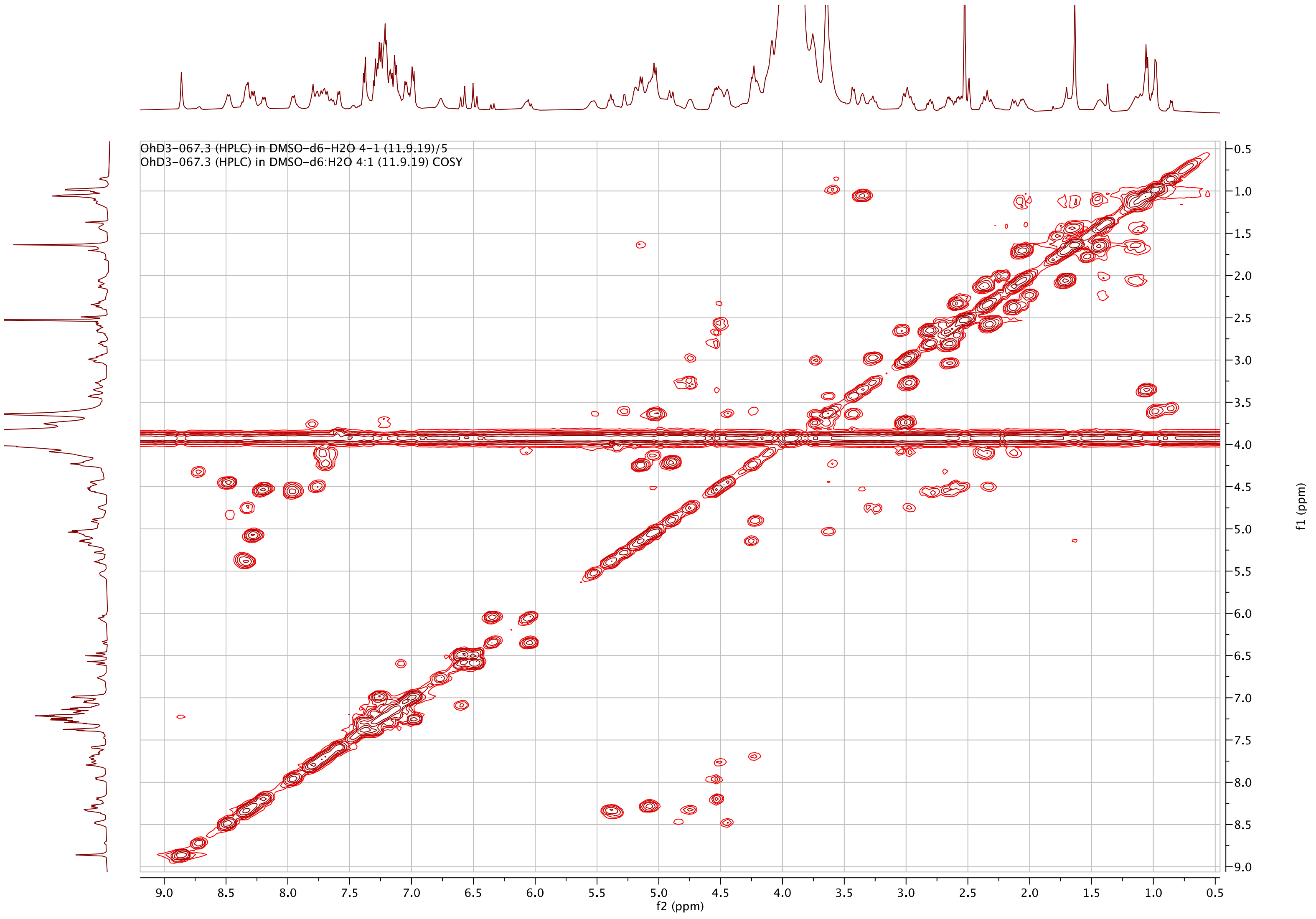

Figure S8. TOCSY spectrum of theopalauamide (**4**) in 4:1 DMSO-d_6:H_2O at 50 ºC

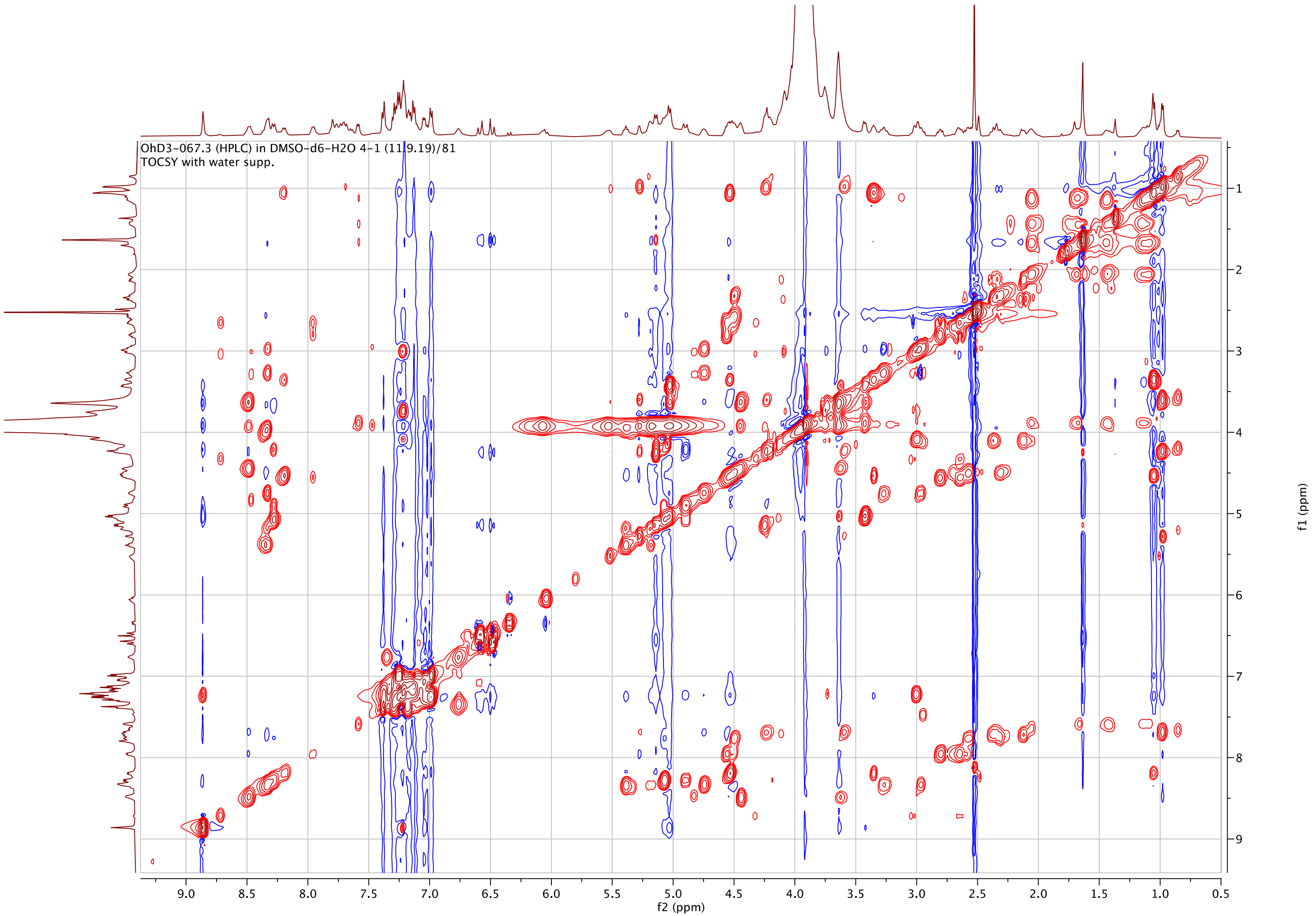

Figure S9. ROESY spectrum of theopalauamide (**4**) in 4:1 DMSO-d_6:H_2O at 50 ºC

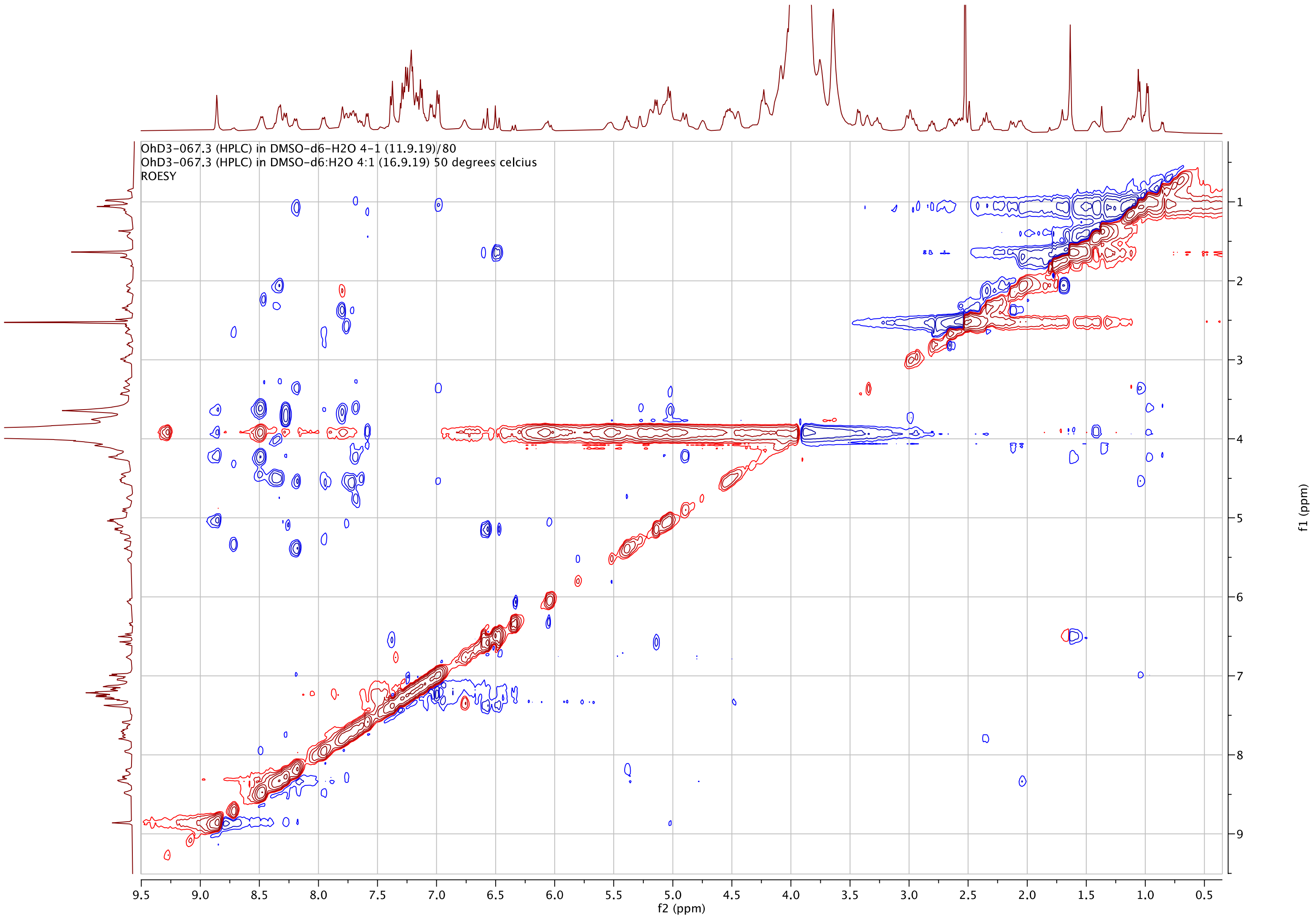

Figure S10. N-H HSQC spectrum of theopalauamide (**4**) in 4:1 DMSO-d_6:H_2O at 50 ^oC

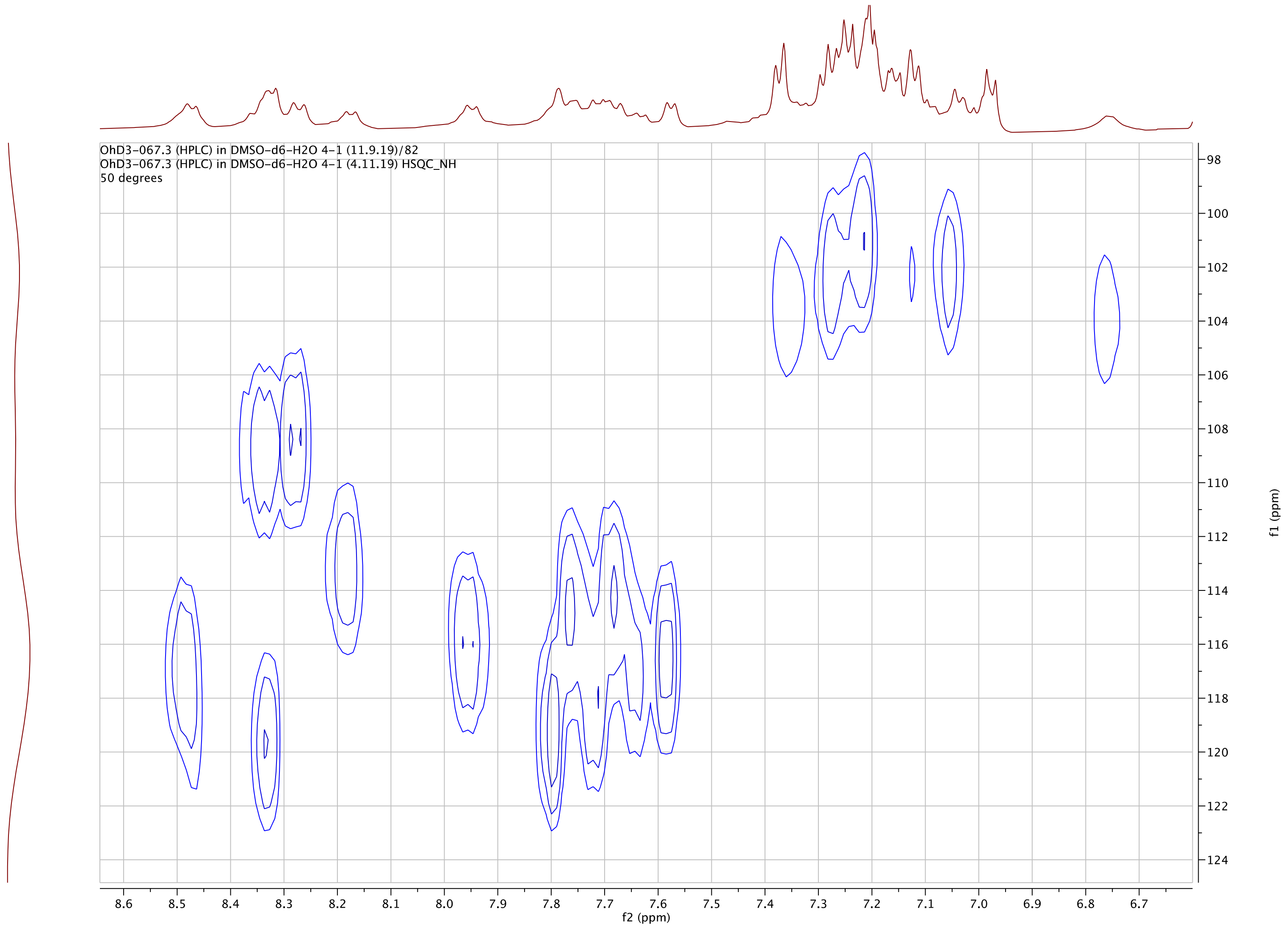

Figure S11. N-H HMBC spectrum of theopalauamide (**4**) in 4:1 DMSO-d_6:H_2O at 50 ºC

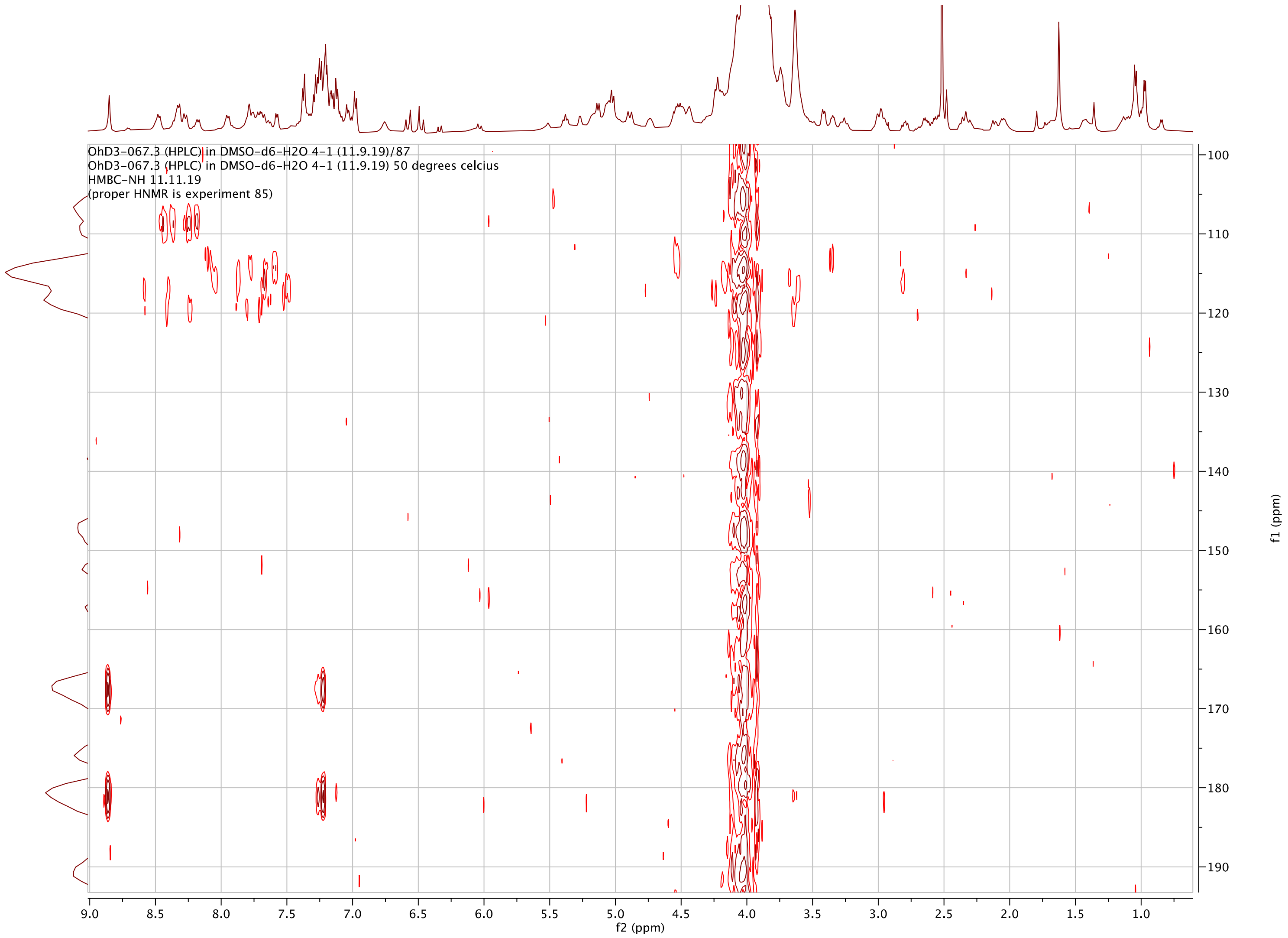

Table S2. NMR data of theopalauamide (**4**) in 4:1 DMSO-d_6:H_2O at 50 $^oC^a$

Position	$\delta_{C/N}$[b]	δ_H[c]	HMBC correlations[d]	COSY correlations	NOE correlations[e]
Apoa-1	172.6, C				
2a	37.6, CH_2	2.37, m	Apoa-1,3	Apoa-2b,3	Ser[1]-NH, Apoa-NH
b		2.10, brd (10.7)	Apoa-1,3	Apoa-2a,3	
3	53.0, CH	4.11, m		Apoa-2a,2b, NH	Apoa-NH
3-NH	*117.7,* NH	7.72, d (9.7)	Phe-1	Apoa-3	Apoa-2a,3,4, 5, Phe-2
4	68.7, CH	4.25, m	Apoa-5	Apoa-5	Phe-NH, Apoa-NH,4-OH
4-OH		5.28, d (4.4)	Apoa-5		Apoa-4,5
5	132.8, CH	5.14, brd (8.3)	Apoa-3,6-Me,7	Apoa-4,6-Me,	Apoa-NH, 7,8
6	136.3, C				
6-Me	13.5, CH_3	1.64, brs	Apoa-5,6,7	Apoa-5	Apoa-7,8
7	134.0, CH	6.58, d (17.1)	Apoa-5,6,6-Me,8,9	Apoa-8	Apoa-5,6-Me
8	128.6, CH	6.49, d (17.1)	Apoa-6,7,9, 10,10’	Apoa-7	Apoa-5,6-Me
9	138.0, C				
10,10’	127.1, CH x 2	7.38, d (7.6)	Apoa-8,10’, 10,12	Apoa-11,11’	
11,11’	129.7, CH x 2	7.29, t (7.6)	Apoa-9,10, 10’,11’,11	Apoa-10, 10’,12	
12	128.9, CH	7.17, m	Apoa-10,10’	Apoa-11,11’	
Ser[1]-1	172.8, C				
2	56.8, CH	3.76, m	Ser[1]-3	Ser[1]-3	sAla-NH
2-NH	*114.9*, NH	7.80, brs	Apoa-1, Ser[1]-2,3	Ser[1]-2	Apoa-2a, Ser[1]-3
3	61.1, CH_2	3.64, m	Ser[1]-1,2	Ser[1]-2	sAla-NH, Ser[1]-NH
3-OH					
sAla-1	169.87, C				
2	51.4, CH	5.07, m	sAla-1	sAla-2-NH, 3a,3b	Asn-NH, sAla-NH,3b
2-NH	*108.3*, NH	8.28, d (10.3)	Ser[1]-1	sAla-2	sAla-2,3b, Ser[1]-2,3
3a	50.6, CH	4.90, brd (13.7)	sHis-6,8	sAla-2,3b	sHis-8
		4.21, m			
b			sAla-1, sHis-6,8	sAla-2,3a	sHis-6, sAla-NH,2
Asn-1	171.6, C				

2	52.2, CH	4.50, m	Asn-1,3	Asn-NH,3a, 3b	Asn-NH,3b
2-NH	*119.2*, NH	7.77, d (5.3)	sAla-1, Asn-2,3	Asn-2	sAla-2, Asn-2,3a
3a	37.2, CH_2	2.58, dd (15.5, 9.5)	Asn-1,2,4	Asn-2,3b	Asn-N
b		2.35, dd (15.5, 12.9)	Asn-1,2,4	Asn-2,3a	Asn-2
4	172.5, C				
4-NH_2	*103.8*, NH_2	7.37, brs			
		6.76, brs			
Han-1	171.4, C				
2	54.7, CH	5.39, t (8.6)	Han-1,3,4	Han-NH,3	BrMePhe-NH, sHis-2
2-NH	*108.7*, NH	8.35, m	Han-1	Han-2	
3	72.4, CH	3.98, m		Han-2,3-OH	
3-OH		5.19, brd (4.1)		Han-3	
4	174.5, C				
4-NH_2	*102.1*, NH_2	7.05, m	Han-3		
		7.28, m			
BrMePhe-1	172.0, C				
2	59.3, CH	4.54, m	BrMePhe-1,3,3-Me,4	BrMePhe-NH,3	BrMePhe-NH,3,3-Me,5,5’
2-NH	*113.1*, NH	8.19, d (8.7)	Han-1, BrMePhe-2,	BrMePhe-2	Has-2, BrMePhe-2,3,3-Me
3	39.5, CH	3.35, qd (6.8,5.2)	BrMePhe-1,2,3-Me,4,5,5’	BrMePhe-2,3-Me	BrMePhe-NH,2
3-Me	18.0, CH_3	1.05, d (6.8)	BrMePhe-2,3,4	BrMePhe-3	BrMePhe-NH,2,5,5’
4	141.8, C				
5,5’	131.1, CH x 2	6.99, d (8.1)	BrMePhe-3,5’,5,7	BrMePhe-6,6’	BrMePhe-2,3-Me
6,6’	131.7, CH x 2	7.26, d (8.1)	BrMePhe-4,6’,6,7	BrMePhe-5,5’	
7	120.6, C				
iSer-1	171.4, C				
2	69.8, CH	4.09, m	iSer-1	iSer-2-OH, 3a,3b	Aad-NH
2-OH		5.03 m	iSer-2	iSer-2	
3a	44.0, CH_2	3.74, m	BrMePhe-1	iSer-2,3b, NH	
b		3.01, brd (14.1)	BrMePhe-1, iSer-1	iSer-2,3a, NH	
3-NH	*101.2*, NH	7.22, m	BrMePhe-1	iSer-3a,3b	
Aad-1	176.0, C				
2	54.7, CH	3.91, m	Aad-1,3,4		Aad-NH,3a, 3b, sHis-6,8

2-NH	*116.5*, NH	7.59, d (7.5)	iSer-1, Aad-1		Aad-2,3b,4, iSer-2
3a	32.4, CH_2	1.65, m		Aad-3b	Aad-2
b		1.44, m	Aad-4	Aad-3a,4	Aad-NH,2
4	22.2, CH_2	1.12, m		Aad-3b,5a,5b	Aad-NH
5a	35.9, CH_2	2.06, m	Aad-3,4,6	Aad-4,5b	sHis-NH
b		1.70, m	Aad-3,4,6	Aad-4,5a	
6	174.3, C				
sHis-1	171.0, C				
2	54.6, CH	4.75, dt (13.0,6.5)	sHis-1,3	sHis-NH,3a,3b	Thr-NH, Han-2
2-NH	*119.7*, NH	8.33, m	Aad-6, sHis-1,2,3	sHis-2	Ada-2a
3a	26.4, CH_2	3.27, t (13.0)	sHis-1,2,4,8	sHis-2,3b	Thr-NH
b		2.98, m	sHis-1,2,4	sHis-2,3a	Thr-NH
4	132.0, C				
5-N	*180.9*, N				
6	137.4, CH	8.86, s	sHis-5-N,7-N,4,8, Gal-1, sAla-3a,3b	sHis-8	Gal-1,2,5, sAla-3a, Ada-5
7-N	*167.9*, N				
8	124.3, CH	7.22, m	sHis-5-N,7-N,4,6 sAla-3	sHis-6	sAla-3a, Ada-5
Thr-1	172.7, C				
2	58.9, CH	4.23, m	Thr-1,3,4	Thr-NH,3	Ser^2-NH, Thr-NH,4
2-NH	*114.3*, NH	7.69, d (9.7)	sHis-1, Thr-2	Thr-2	sHis-2,3a,3b Thr-2,3,4,
3	69.0, CH	3.60, m	Thr-1	Thr-2,4	Thr-NH,4
3-OH		4.90, m		Thr-2,3	
4	21.6, CH_3	0.98, d (5.3)	Thr-2,3	Thr-3	Thr-NH,2,3
Ser^2-1	169.92, C				
2	56.6, CH	4.45, m			Ser^2-NH
2-NH	*117.0*, NH	8.48, d (7.8)	Thr-1		Ser^2-2,3, Thr-2
3	62.1, CH_2	3.64, m			Ser^2-NH
3-OH					
Phe-1	171.6, C				
2	55.0, CH	4.56, m	Ser^2-1		Phe-NH,5,5’ Apoa-NH
2-NH	*115.9*, NH	7.96, d (8.7)	Ser^2-1		Phe-2,3a,3b, Apoa-4-OH

3a	39.4, CH_2	2.81, dd (13.0,8.0)			Phe-NH
b		2.65, dd (13.0,5.6)			Phe-NH
4	137.3, C				
5,5’	130.0, CH x 2	7.13, d (7.5)			Phe-2
6,6’	129.2, CH x 2	7.21, m			
7	127.7, CH	7.14, m			
Gal-1	89.0, CH	5.03, d (9.3)	sAla-3, sHis-4, Gal-3,5	Gal-2	sHis-6, Gal-3,6b
2	69.9, CH	3.63, m		Gal-1,2-OH, 3	sHis-6
2-OH		5.53, brd (10.4)		Gal-2	
3	73.9, CH	3.43, brd (8.1)	Gal-4	Gal-2	Gal-1
4	69.6, CH	3.83, m	Gal-2,3		
5	79.0, CH	3.64, m	Gal-1,3,6		sHis-6
6a	62.0, CH_2	3.75, m	Gal-5	Gal-6b	
b		3.64, m		Gal-6a	Gal-1

[a] ^1H (500 MHz), ^{13}C (125 MHz) and ^{15}N (50 MHz). [b] Multiplicity and assignment from HSQC experiment. [c] Multiplicity (*J* in Hz). [d] HMBC correlations, optimized for 8 Hz. [e] Selected NOEs from ROESY experiment.

Figure S12. (+)-HRESIMS data of theopalauamide (**4**)

Elemental Composition Report

Single Mass Analysis

Tolerance = 5.0 mDa / DBE: min = -1.5, max = 50.0
Element prediction: Off
Number of isotope peaks used for i-FIT = 3

Monoisotopic Mass, Odd and Even Electron Ions
114 formula(e) evaluated with 9 results within limits (up to 50 best isotopic matches for each mass)
Elements Used:
C: 70-80 H: 90-100 N: 10-20 O: 23-33 79Br: 0-1

ohD3-067/3 Carmeli1317H 197 (7.467) Cm (195:207)
Ohad Hasin
1: TOF MS ES-

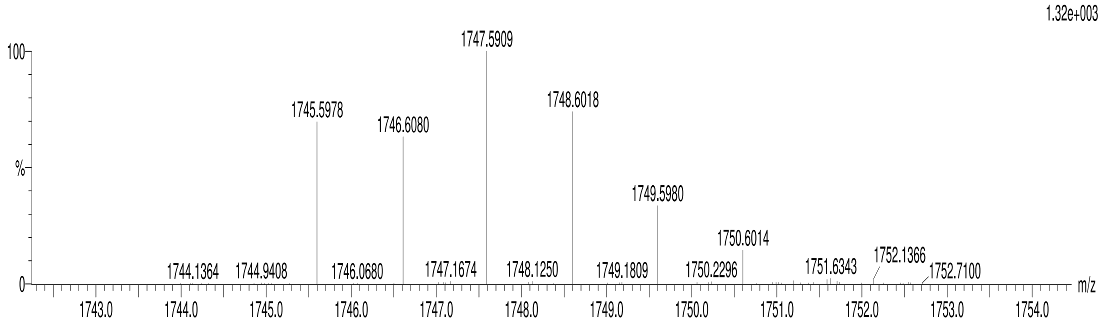

Mass	Calc. Mass	mDa	PPM	DBE	i-FIT	i-FIT (Norm)	Formula
Minimum:				-1.5			
Maximum:		5.0	10.0	50.0			
1745.5978	1745.5965	1.3	0.7	38.0	51.6	2.0	C74 H91 N17 O33
	1745.5957	2.1	1.2	36.0	51.6	2.1	C74 H96 N19 O26 79Br
	1745.5944	3.4	1.9	31.0	51.6	2.1	C73 H100 N15 O30 79Br
	1745.5971	0.7	0.4	35.5	51.7	2.1	C76 H98 N16 O27 79Br ✓
	1745.5930	4.8	2.7	31.5	51.7	2.2	C71 H98 N18 O29 79Br
	1745.5984	-0.6	-0.3	40.5	51.7	2.2	C77 H94 N20 O23 79Br
	1745.5984	-0.6	-0.3	35.0	51.8	2.2	C78 H100 N13 O28 79Br
	1745.5997	-1.9	-1.1	40.0	51.9	2.4	C79 H96 N17 O24 79Br
	1745.6005	-2.7	-1.5	42.0	52.0	2.4	C79 H91 N15 O31

Figure S13. ^{1}H NMR spectrum (500 MHz) of theonellamide J (**1**) in 4:1 DMSO-d_6:H_2O at 50 ^{o}C

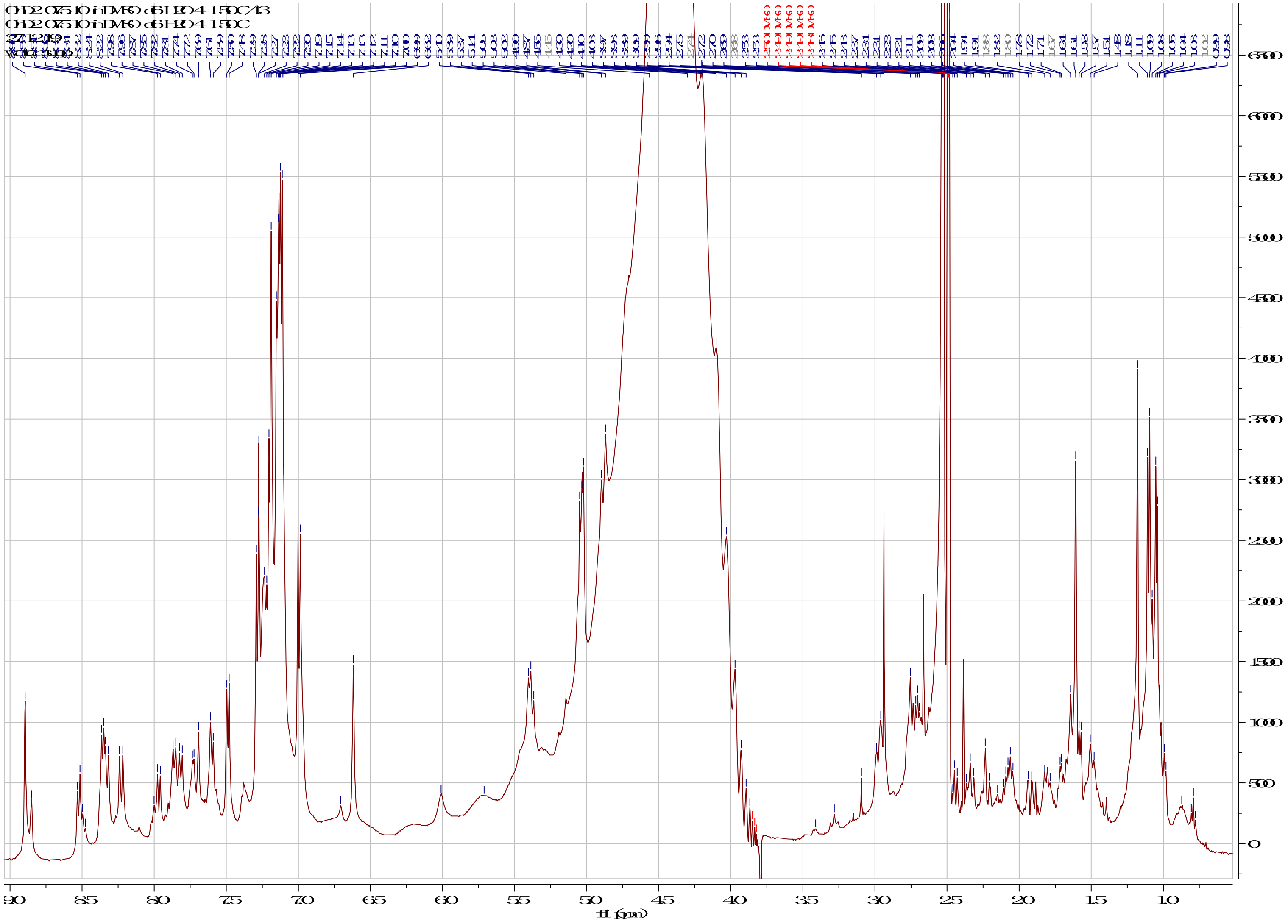

Figure S14. ^{13}C NMR spectrum (125 MHz) of theonellamide J (**1**) in 4:1 DMSO-d_6:H_2O at 50 ^{o}C

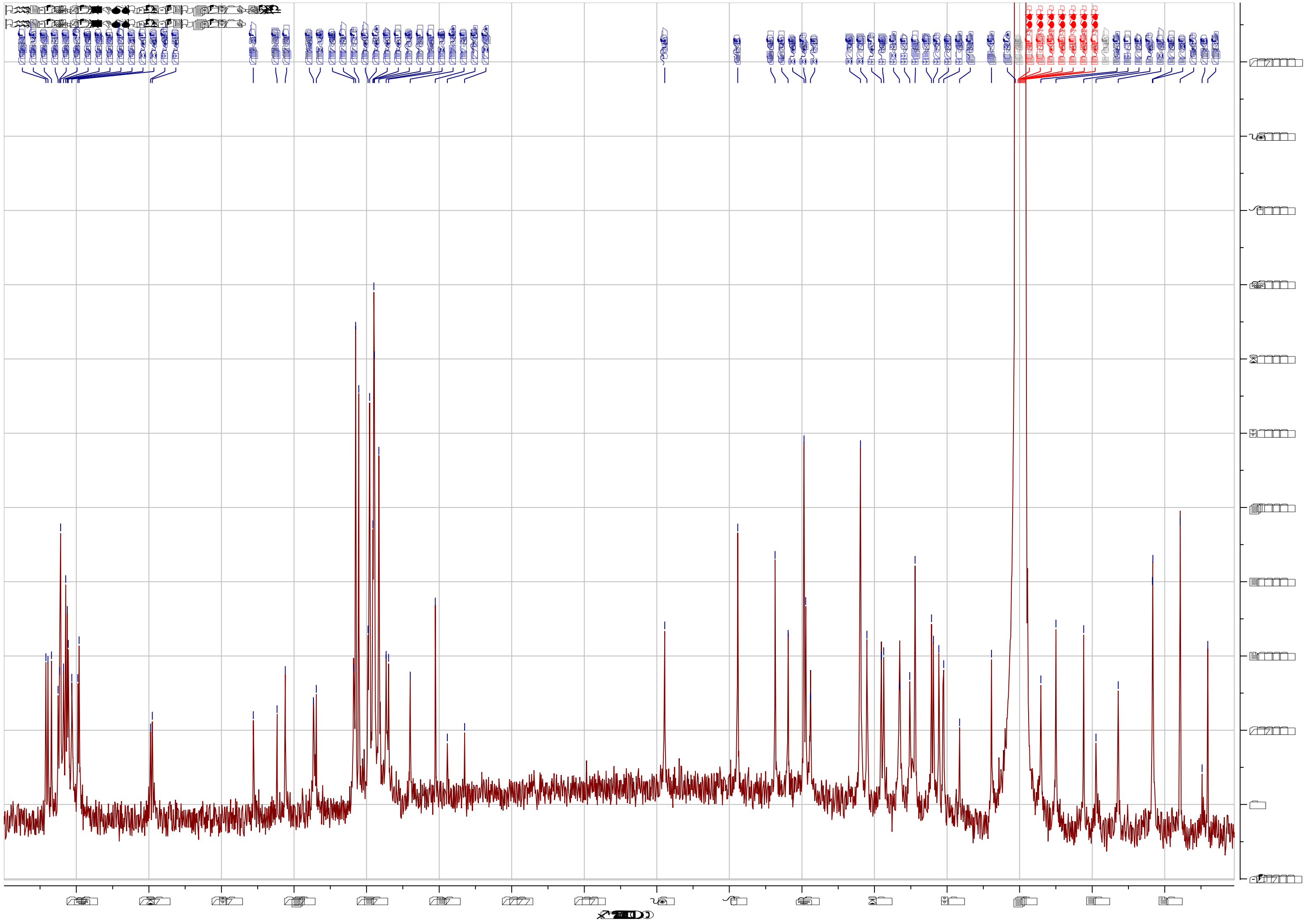

Figure S15. HSQC spectrum of theonellamide J (**1**) in 4:1 DMSO-d_6:H_2O at 50 ºC

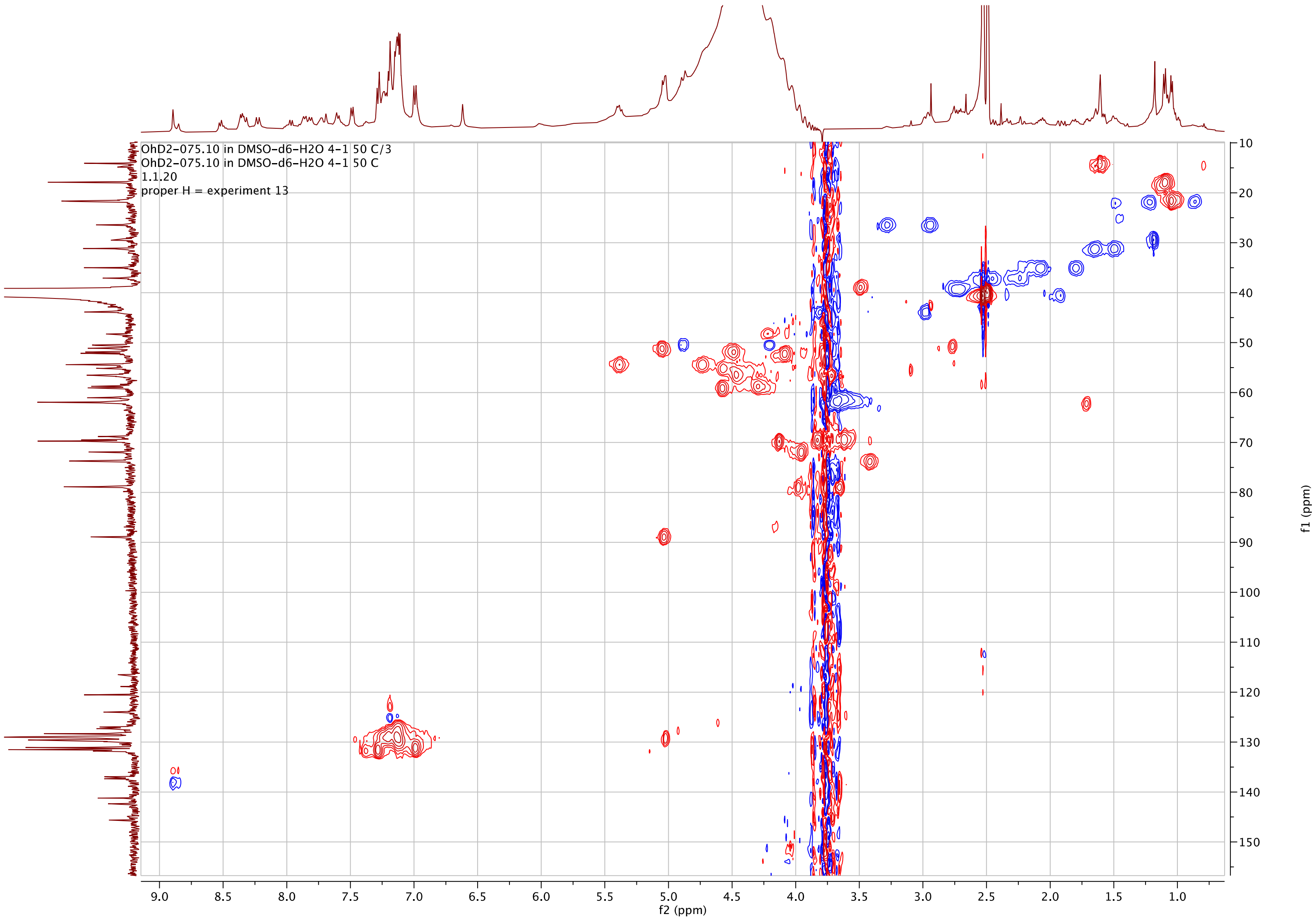

Figure S16. HMBC spectrum of theonellamide J (**1**) in 4:1 DMSO-d_6:H_2O at 50 ^oC

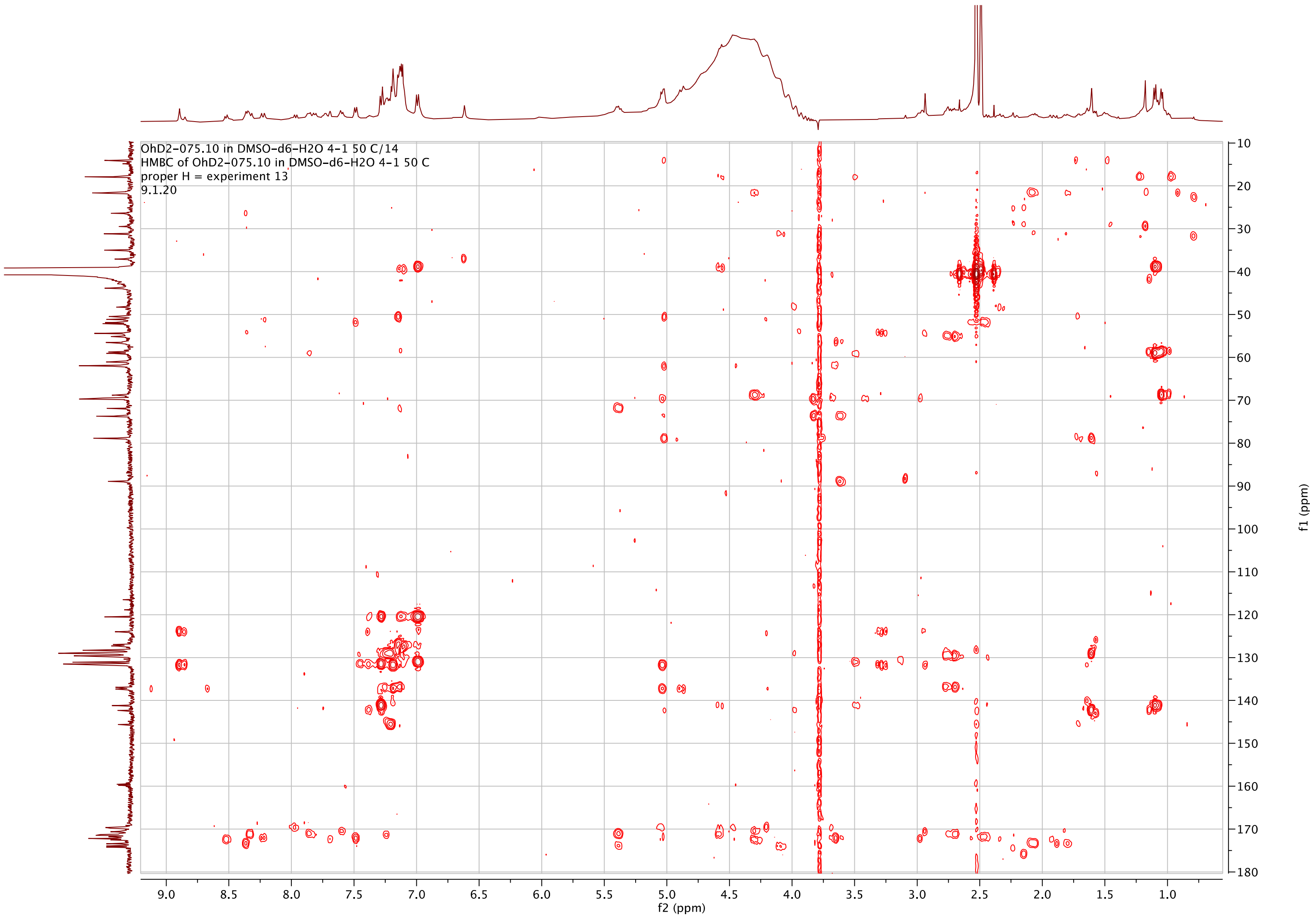

Figure S17. COSY spectrum of theonellamide J (**1**) in 4:1 DMSO-d_6:H_2O at 50 ºC

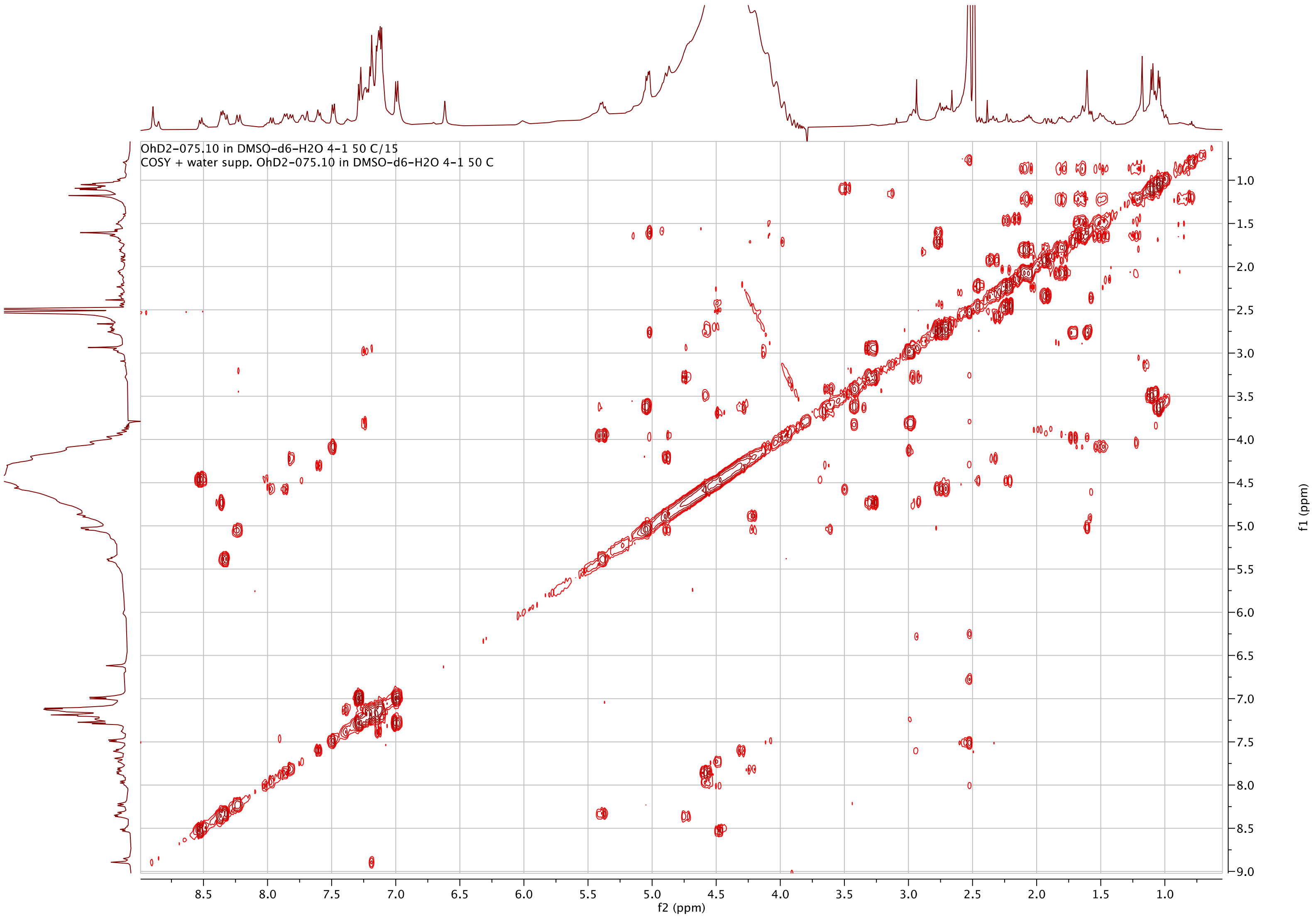

Figure S18. TOCSY spectrum of theonellamide J (**1**) in 4:1 DMSO-d_6:H_2O at 50 ºC

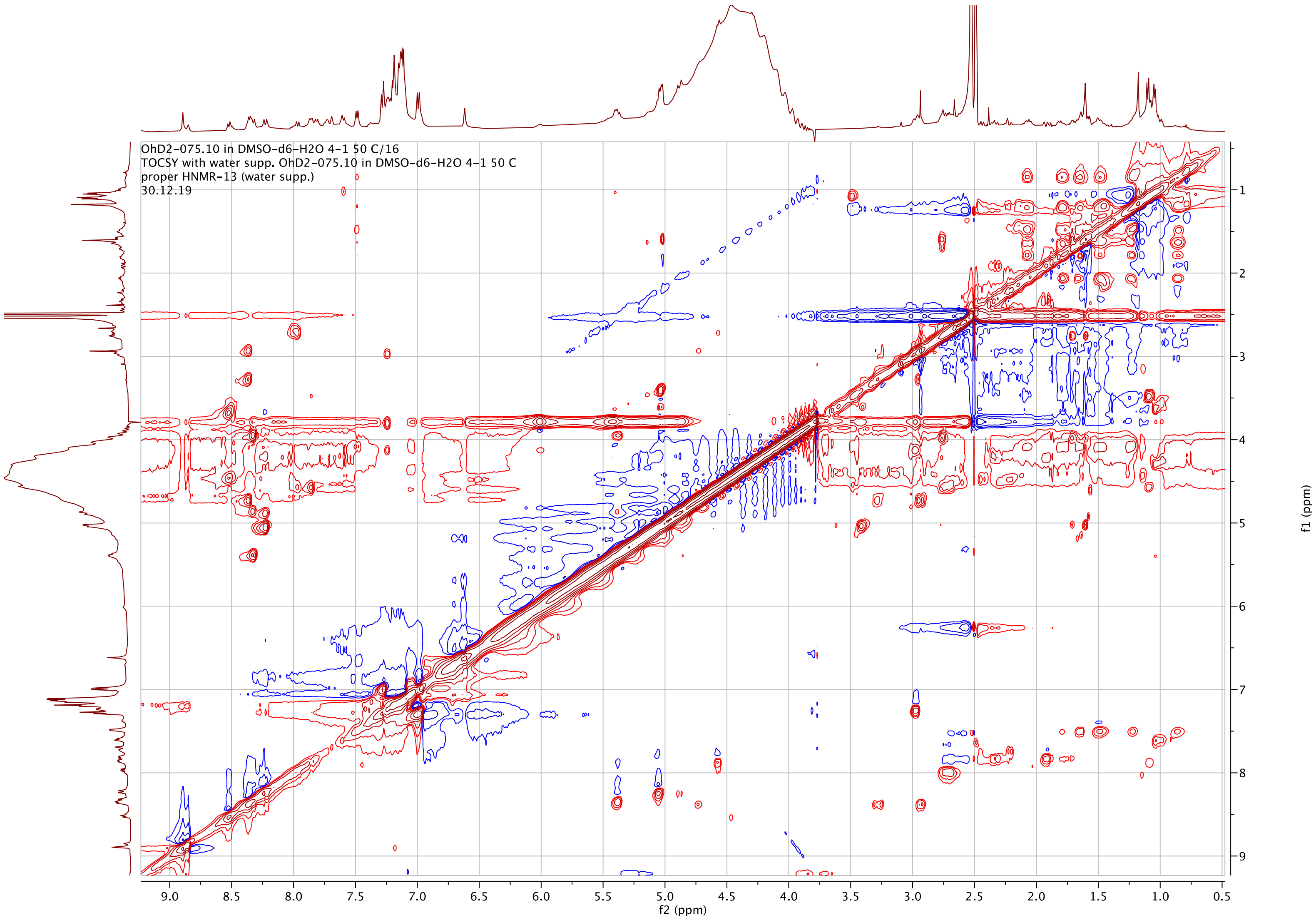

Figure S19. ROESY spectrum of theonellamide J (**1**) in 4:1 DMSO-d_6:H_2O at 50 ºC

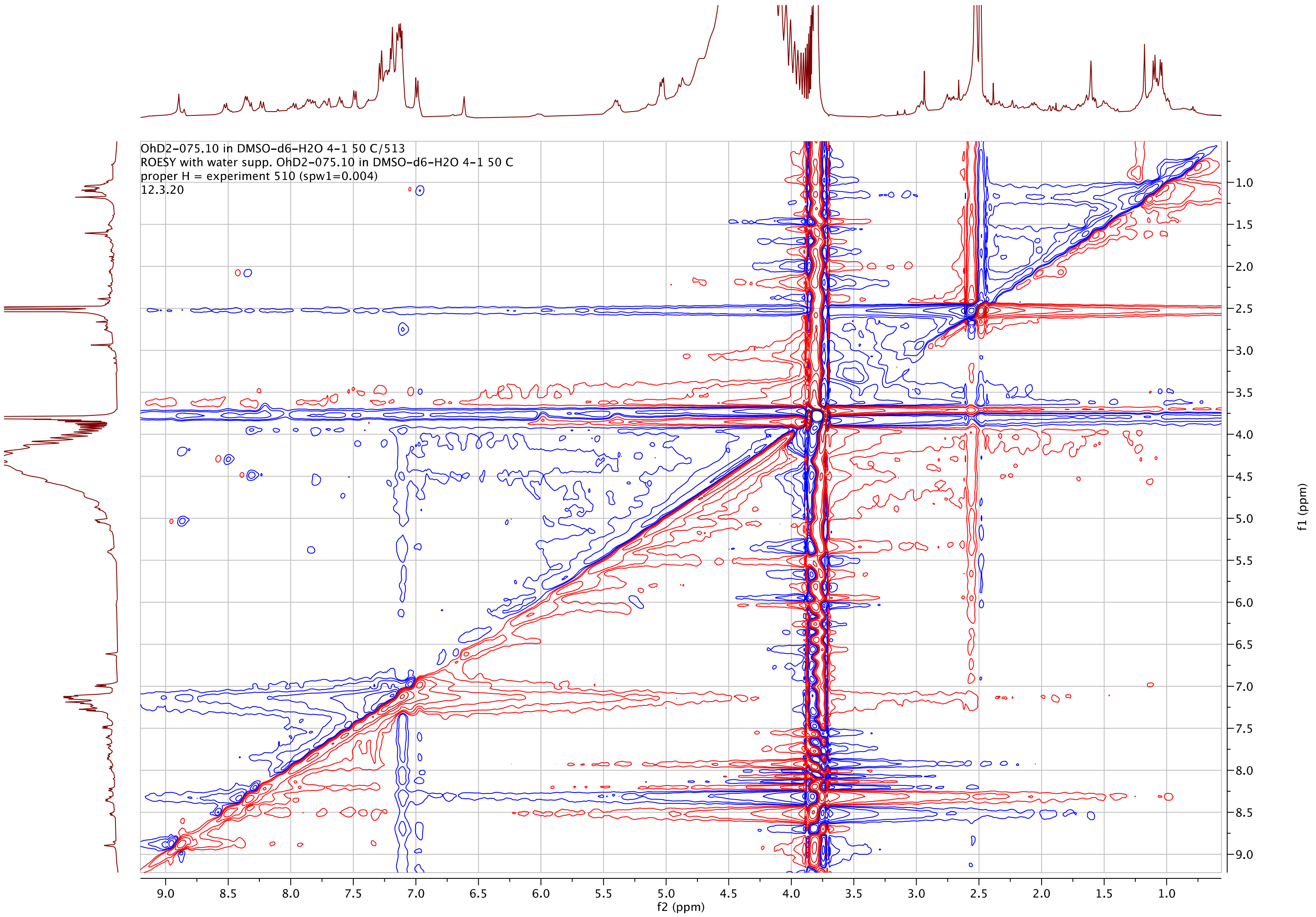

Figure S20. N-H HSQC spectrum theonellamide J (**1**) in 4:1 DMSO-d_6:H_2O at 50 ºC

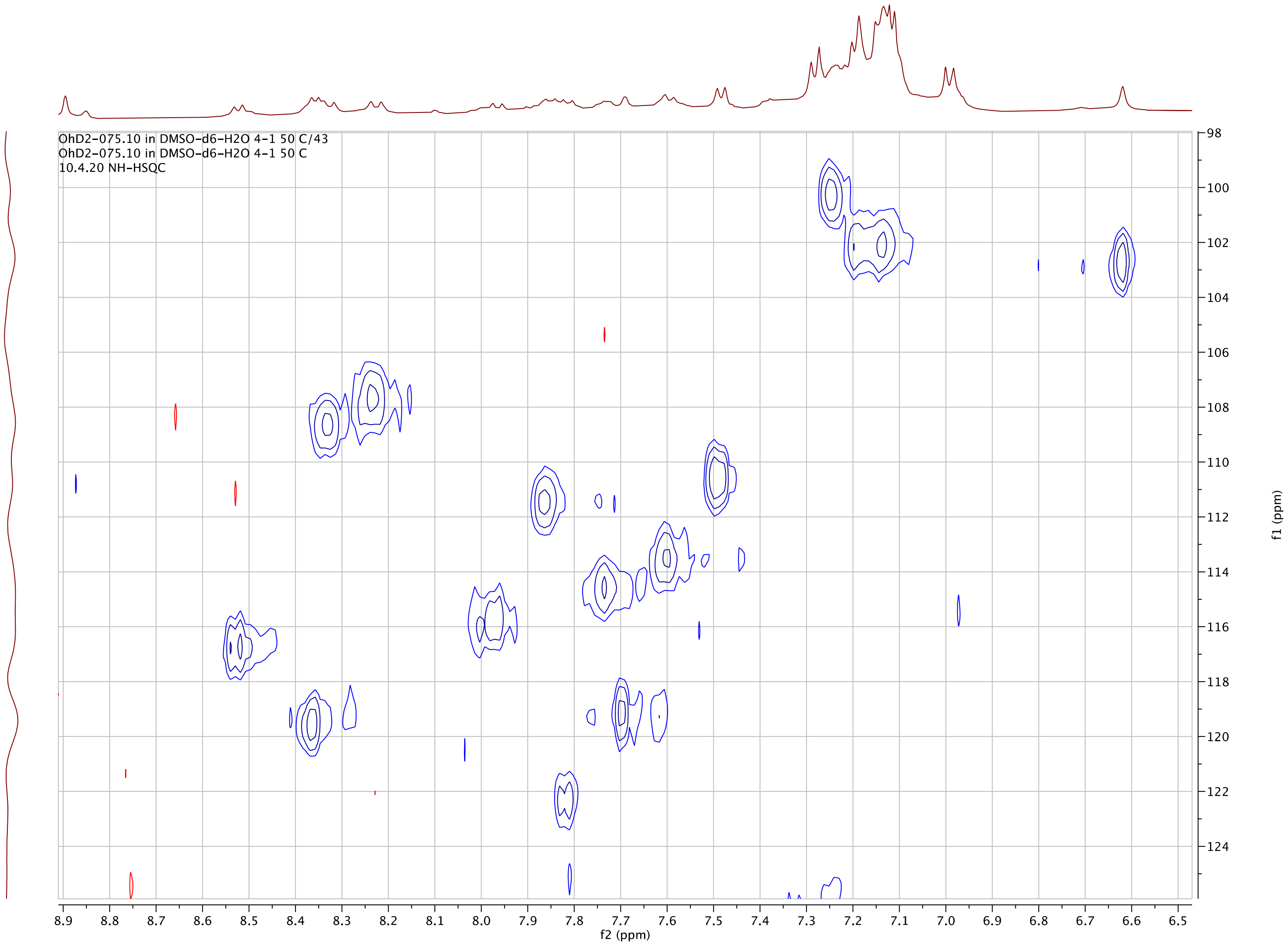

Figure S21. N-H HMBC spectrum of theonellamide J (**1**) in 4:1 DMSO-d_6:H_2O at 50 ºC

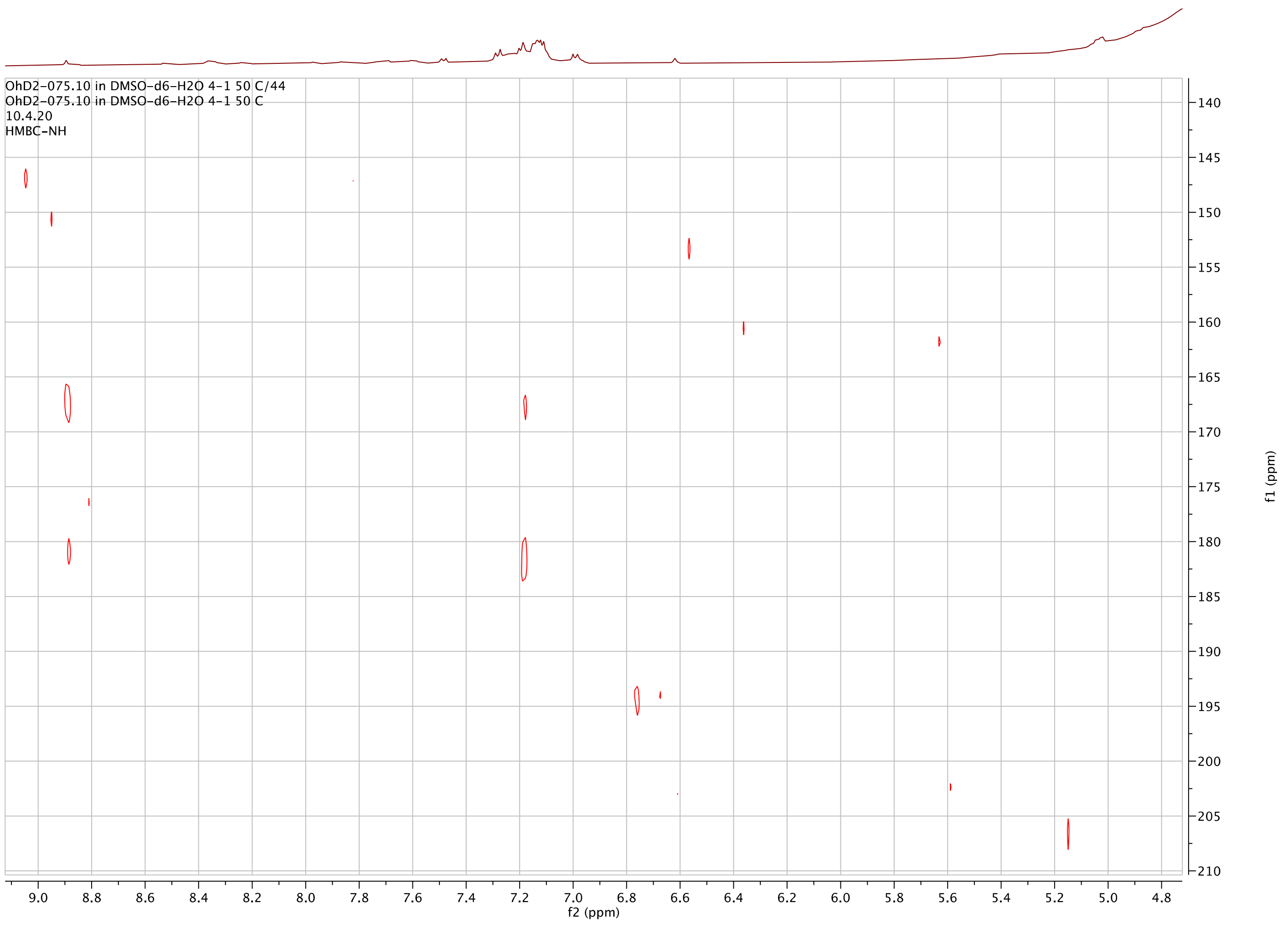

Table S3. NMR data of theonellamide J (**1**) in 4:1 DMSO-d_6:H_2O at 50 $^oC^a$

Position	$\delta_{C/N}$[b]	δ_H[c]	HMBC correlations[d]	COSY correlations	TOCSY correlations
Apcoa-1	171.8, C				
2a	40.4, CH_2	2.33, t (13.1)	Apcoa-3	Apcoa-2b,3	Apcoa-2b,3
b		1.93, dd (13.1,3.0)	Apcoa-1	Apcoa-2a,3	Apcoa-2a,3
3	48.3, CH	4.22, m		Apcoa-2a,3-NH	Apcoa-NH-3
3-*NH*	*122.3, NH*	7.82, d (9.1)	Phe-1	Apcoa-3	Apcoa-2a,2b,3 ,4
4	62.1, CH	1.71, ddd (10.4, 6.6., 4.5)	Apcoa-5,6,7,9		Apcoa-3-NH,5,7,8
5	78.9, CH	3.98, m	Apcoa-3	Apcoa-6-Me	Apcoa-4,6-Me, 8
6	142.3, C				
6-Me	14.1, CH_3	1.61, brs	Apcoa-5,6,7	Apcoa-7	Apcoa-5,7,8
7	129.1, CH	5.02, brs	Apcoa-4,6,8	Apcoa-5,6-Me	Apcoa-4,6-Me, 8
8	50.6, CH	2.76, m	Apcoa-10,10’	Apcoa-4,6-Me, 7	Apcoa-4,5,6-Me,7
9	145.6, C				
10,10’	128.3, CH x 2	7.14, d x 2 (7.6)	Apcoa-8,10’,10,12		
11,11’	129.0, CH x 2	7.20, t x 2 (7.6)	Apcoa- 9,11’,11		
12	127.0, CH	7.13, m			
Ser[1]-1	172.3, C				
2	56.6, CH	3.71, m			
2-NH	*119.2, NH*	7.69, brs			
3	61.9, CH_2	3.62, m 2H	Ser[1]-1,3		
sAla-1	169.6, C				
2	51.1, CH	5.04, m	sAla-1	sAla-2-NH,3a, 3b	
2-NH	*107.7, NH*	8.23, d (11.1)	Ser[1]-1, sAla-2	sAla-2,3a,3b	sAla-2,3a, 3b
3a	50.5, CH_2	4.88, d (13.2)	sAla-2, sHis-6	sAla-2,3a,3b	
b		4.22, m	sAla-1,2, sHis-8		
Asn-1	171.5, C				
2	51.9, CH	4.48, brm	Asn-1	Asn-2-NH,3a,3b	Asn-2-NH
2–NH	*114.6, NH*	7.73, d (5.5)	Asn-1	Asn-2	Asn-2,3a,3b
3a	37.1, CH_2	2.45, m	Asn-4,2	Asn-2,3b	Asn-2-NH,

b		2.24, m		Asn-2,3a	Asn-2-NH
4	172.2, C				
4–NH_2	102.7, NH_2	7.31, brs			
		6.62, s	Asn-3		
Han-1	171.5, C				
2	54.4, CH	5.39, m	Han-1,3,4	Han-2-NH,3	Han-2-NH,3
2–NH	*108.7, NH*	8.33, d (10.3)		Han-2	Han-2,3,3-OH
3	71.9, CH	3.95, m		Han-2,3-OH	Han-2,2-NH
3–OH		4.87, m			Han-2,2-NH
4	174.2, C				
4–NH_2	*102.2,* NH_2	7.14, brs,			
		7.19, brs			
BrMePhe-1	171.1, C				
2	59.1, CH	4.58, m	BrMePhe-1	BrMePhe-2-NH,3	BrMePhe-2-NH
2-NH	*111.5, NH*	7.86, d (9.1)	Han-1	BrMePhe-2	BrMePhe-2,3-Me3
3	38.9, CH	3.49, m	BrMePhe-2,3,3-Me,4	BrMePhe-2	BrMePhe-2-NH, 3-Me
3-Me	17.9, CH_3	1.10, d (7.2)	BrMePhe-2,3,4	BrMePhe-3	BrMePhe-2-NH, 3
4	141.2, C				
5,5’	131.1, CH x 2	6.99, d x 2 (8.4)	BrMePhe-3,5’, 5,7	BrMePhe-6,6’	
6,6’	131.5, CH x 2	7.28, d x 2 (8.4)	BrMePhe-4,5, 5’,6’,6,7	BrMePhe-5,5’	
7	120.5, C				
iSer-1	172.2, C				
2	69.7, CH	4.13, m		iSer-3b	iSer-3b,3-NH
2-OH		5.42, brs	iSer-2		
3a	43.9, CH_2	3.81, m	iSer-2	iSer-3b	iSer-3-NH
b		2.98, brd	BrMePhe-1, iSer-1,2	iSer-2,3a,3-NH	iSer-2,3-NH
3-NH	*100.3, NH*	7.24, m	BrMePhe-1	iSer-3a,3b	iSer-2,3a,3b
Ada-1	173.9, C				
2	52.2, CH	4.08, m	Ada-2	Ada-2-NH,3a, 3b	Ada-3a,3b,4a, 4b,5a,5b
2-NH	*110.6, NH*	7.49, d (7.2)	iSer-1, Ada-2	Ada-2	Ada-2,3a,3b, 4a,4b,5a,5b
3a	31.2, CH_2	1.66, m		Ada-2,3b,4a, 4b	Ada-2,3b,4a, 4b,5a,5b

b		1.51, m	Ada-5	Ada-2,3a,3b, 4a	Ada-2,3a,4a, 4b,5a,5b
4a	21.7, CH_2	1.22, m		Ada-3a,3b,4b, 5a,5b	Ada-2,3a,3b, 4b,5a,5b
b		0.88, m		Ada-3a,3b,4a, 5a,5b	Ada-2,3a,3b, 4a,5a,5b
5a	35.0, CH_2	2.08, m	Ada-3,4,6	Ada-4a,4b,5b	Ada-2,3a,3b, 4a,4b,5b
b		1.80, m	Ada-4,6	Ada-4a,4b,5a	Ada-2,3a,3b, 4a,4b,5a
6	173.4, C				
sHis-1	170.6, C				
2	54.4, CH	4.73, m		sHis-2-NH, 3a,3b	sHis-2-NH, 3a,3b
2-NH	*119.6, NH*	8.36, d (7.2)	Ada-6	sHis-2	sHis-2,3a,3b
3a	26.4, CH_2	3.28, brt (13.1)	sHis-2,4,8	sHis-2,3b	sHis-2,2-NH,3b
b		2.94, brd (13,1)	sHis-1	sHis-2,3a	sHis-2,2-NH,3a
4	131.9, C				
5-N	*181.0, N*				
6	137.3, CH	8.89, s	sHis-3,4,5,8,7-N, sAla-3	sHis-8	
7-N	*167.4, N*				
8	124.0, CH	7.19, s	sHis-4,5-N,6,7-N	sHis-6	
Thr-1	172.5, C				
2	58.7, CH	4.29, m	sHis-1, Thr-1,3, 4	Thr-2-NH,3	Thr-2-NH
2-NH	*113.5, NH*	7.60, d (9.7)	sHis-1	Thr-2	Thr-2,3,4
3	68.8, CH	3.63, m		Thr-2,3-OH,4	Thr-2-NH,4 7
3-OH		5.40, brs			Thr-4
4	21.6, CH_3	1.04, d (6.0)	Thr-2,3	Thr-3	Thr-2-NH,3,3-OH
Ser^2-1	169.8, C				
2	56.5, CH	4.46, m	Ser^2-1	Ser^2-2-NH,3	Ser^2-2-NH
2-NH	*116.7, NH*	8.52, d (7.6)	Thr-1	Ser^2-2	Ser^2-2,3
3	62.0, CH_2	3.68, m		Ser^2-2	Ser^2-2-NH
Phe-1	171.5, C				
2	55.2, CH	4.56, m		Phe-2-NH,3a, 3b	Phe-2-NH,3a, 3b
2-NH	*115.8, NH*	7.97, d (9.1) (8.01, d)	Ser^2-1	Phe-2	Phe-2,3a,3b
3a	39.2, CH_2	2.77, m	Phe-2,4,5,5’	Phe-2	Phe-2-NH
b		2.72, m	Phe-1,2,4,5,5’	Phe-2	Phe-2-NH,

4	136.9, C				Phe-2
5,5’	129.6, CH x 2	7.10, m	Phe-4,7		
6,6’	128.9, CH x 2	7.14, m	Phe-4,6’,6		
7	127.3, CH	7.13, m			
Gal-1	88.9, CH	5.03, d (8.6)	Gal-2,5, sHis-4,6	Gal-2	Gal-2,3
2	69.5, CH	3.61, m	Gal-1,3	Gal-1,3	Gal-1,3
3	73.7, CH	3.41, brd (9.0)		Gal-2,4	Gal-1,2
4	69.7, CH	3.83, m	Gal-4	Gal-3	
5	78.9, CH	3.66, m	Gal-6		
6a	61.9, CH_2	3.65, m			
b		3.58, m	Gal-4		

[a]^{1}H (500 MHz), ^{13}C (125 MHz) and ^{15}N (50 MHz). Multiplicity and assignment from HSQC experiment. [c]Multiplicity (*J* in Hz).
[d]HMBC correlations, optimized for 8 Hz.

Figure S22. ^{1}H NMR spectrum (500 MHz) of theonellamide J (**1**) after dissolving in 4:1 DMSO-d_6:D_2O at 50 ^{o}C

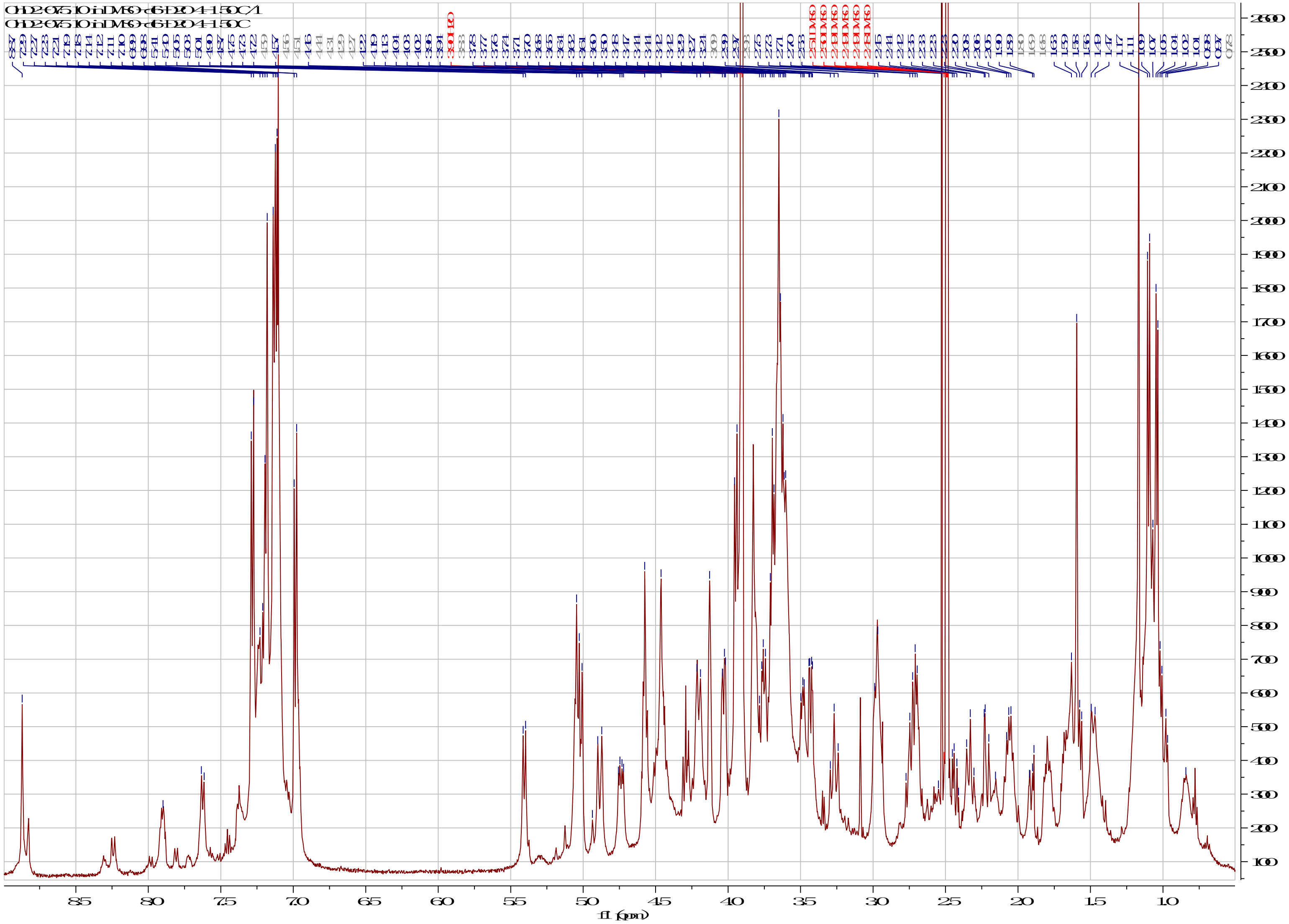

Figure S23. ^{1}H NMR spectrum (500 MHz) of theonellamide J (**1**) 24 h after dissolving in 4:1 DMSO-d_6:D_2O at 50 ^{o}C

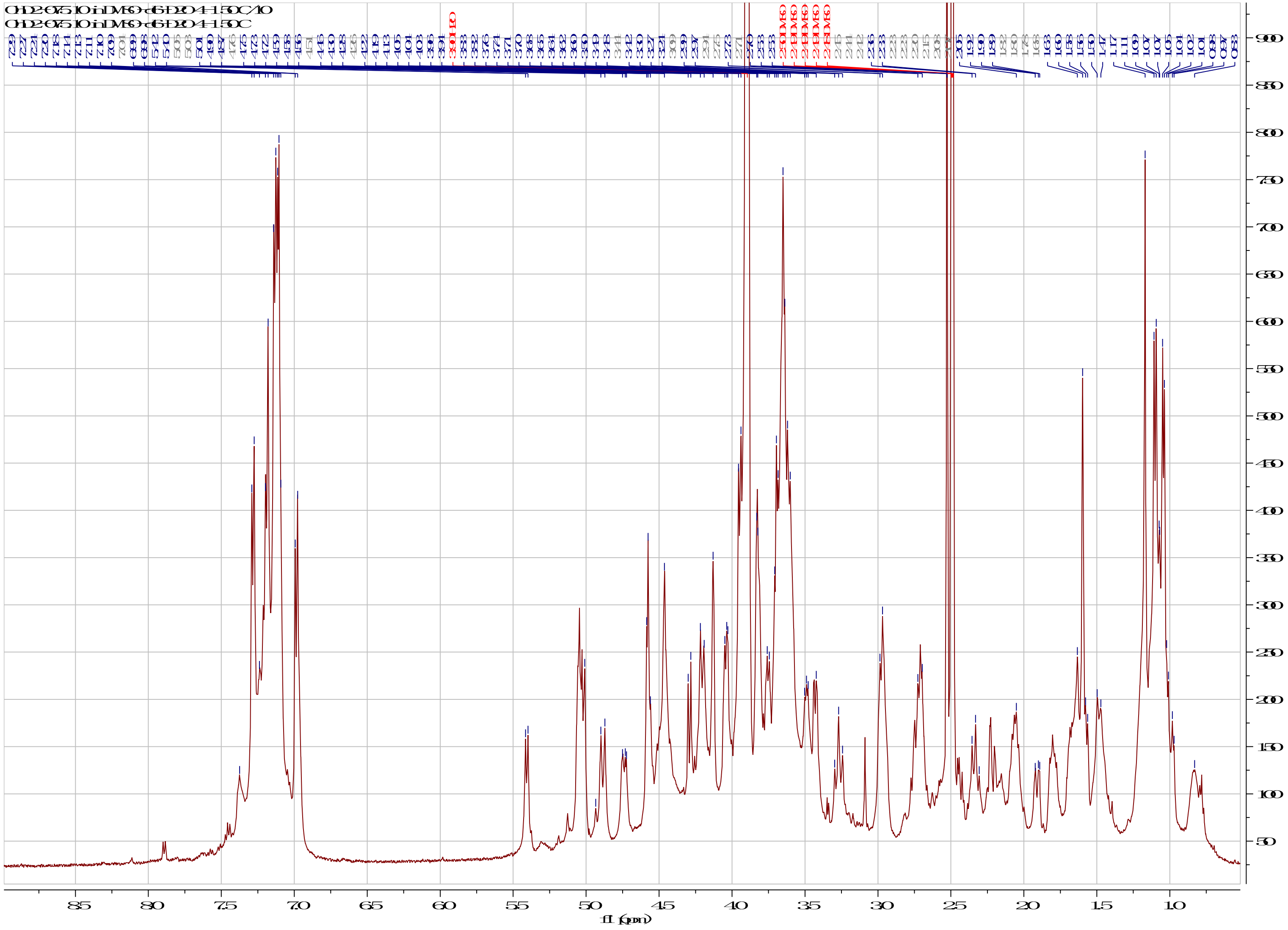

Figure S24. ^{13}C NMR spectrum (125 MHz) of theonellamide J (**1**) in 4:1 DMSO-d_6:D_2O at 50 ^{o}C

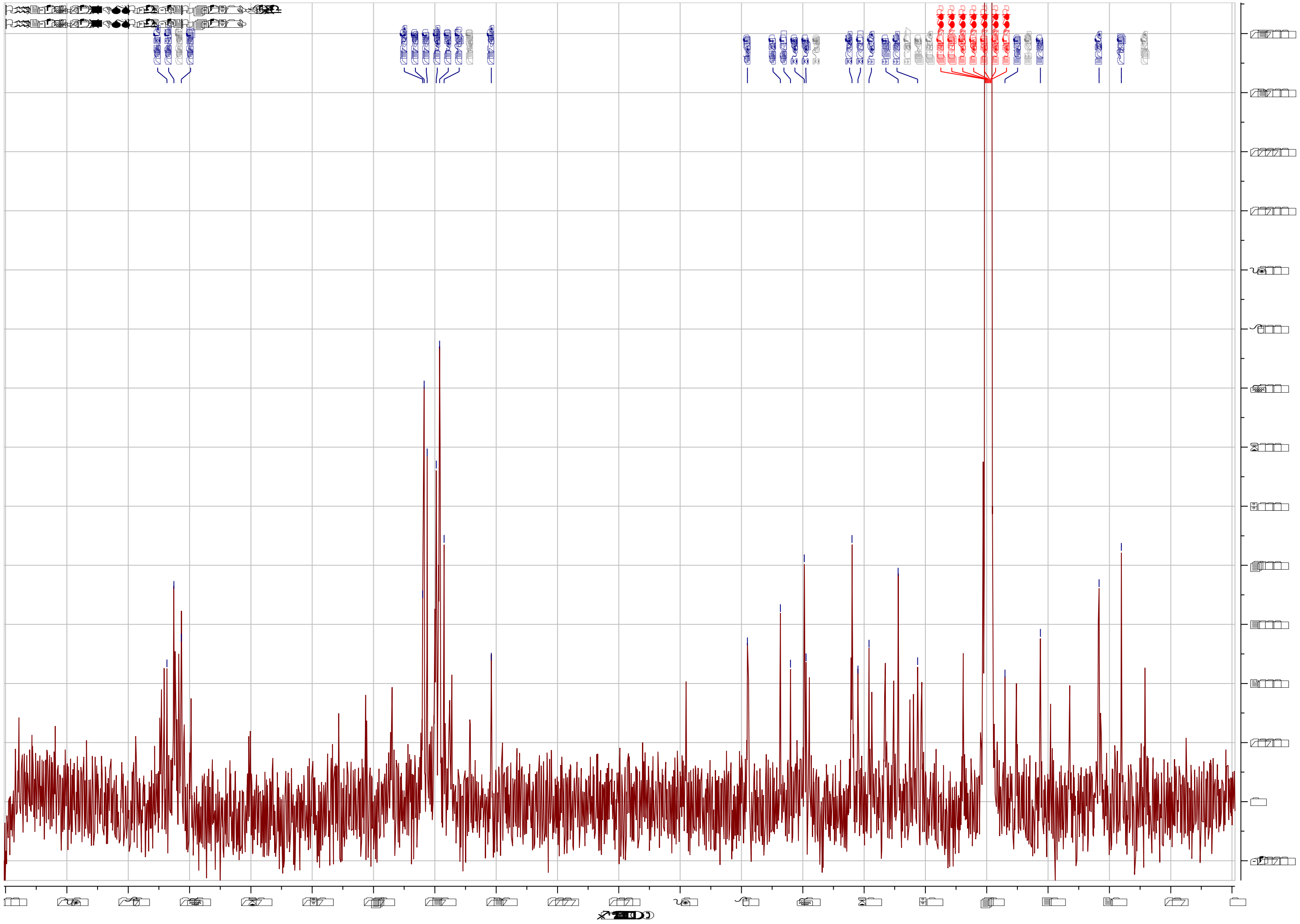

Figure S245. HSQC spectrum of theonellamide J (**1**) in 4:1 DMSO-d_6:D_2O at 50 ºC

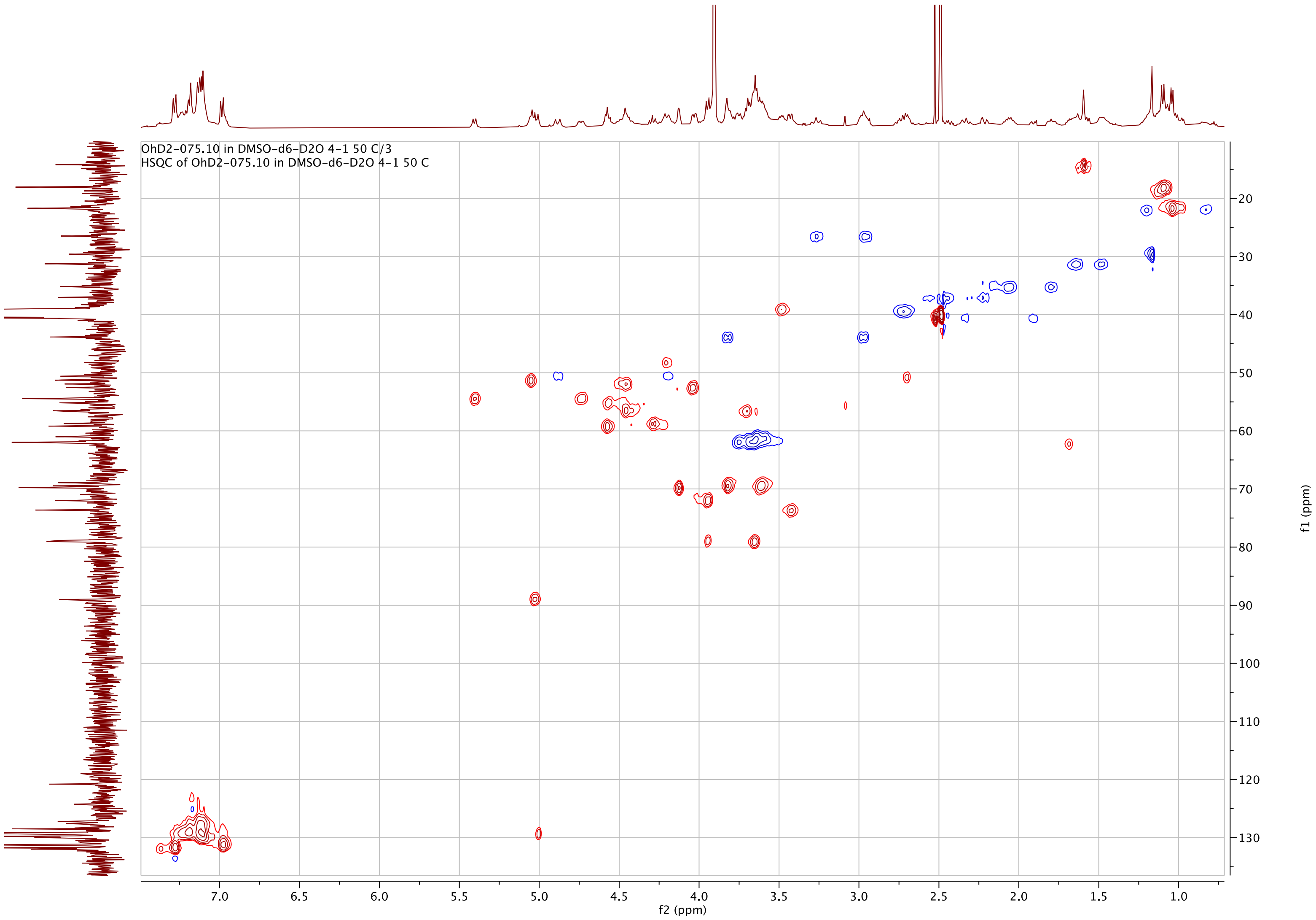

Figure S26. HMBC spectrum of theonellamide J (**1**) in 4:1 DMSO-d_6:D_2O at 50 ^{o}C

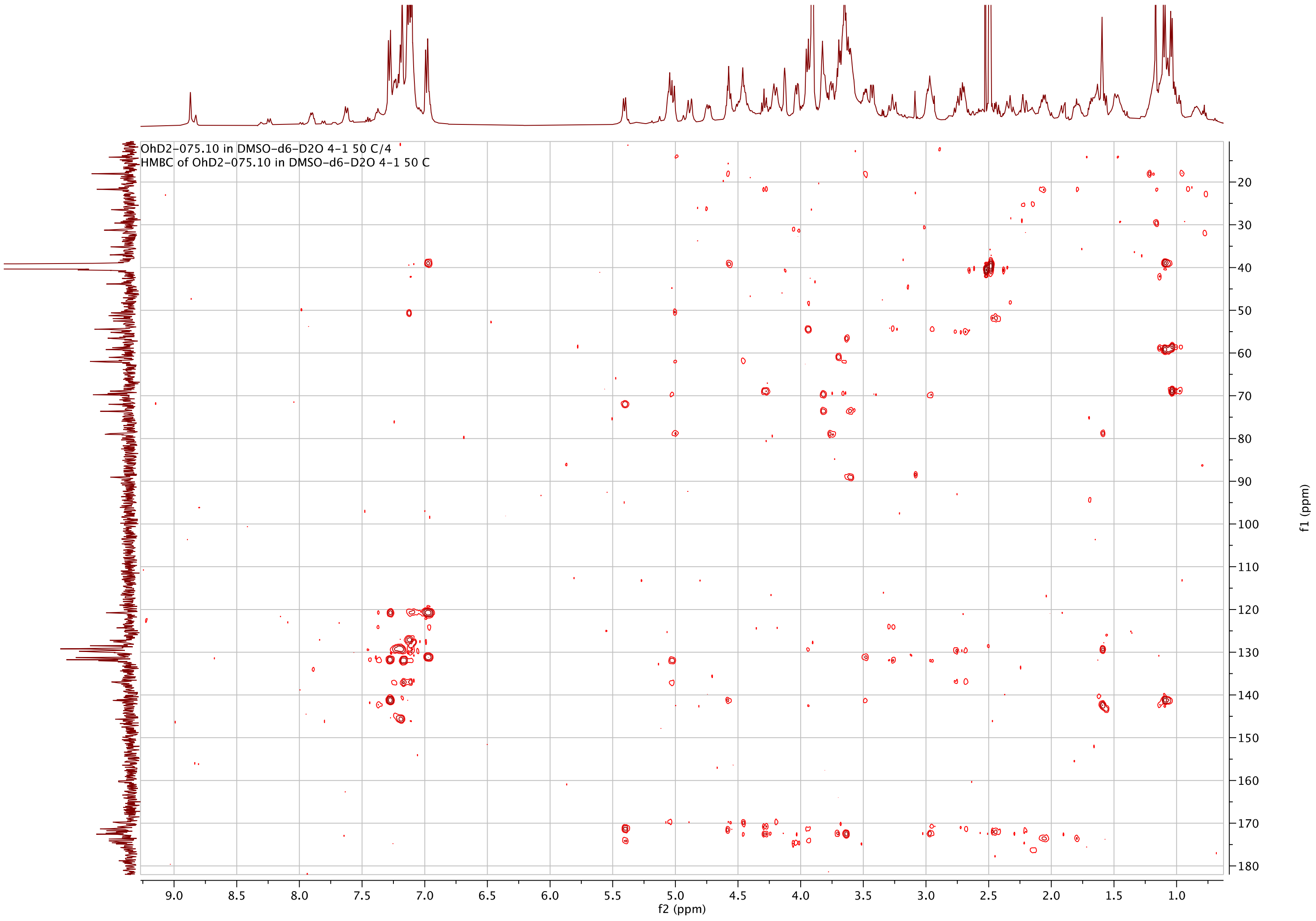

Figure S27. COSY spectrum of theonellamide J (**1**) in 4:1 DMSO-d_6:D_2O at 50 ºC

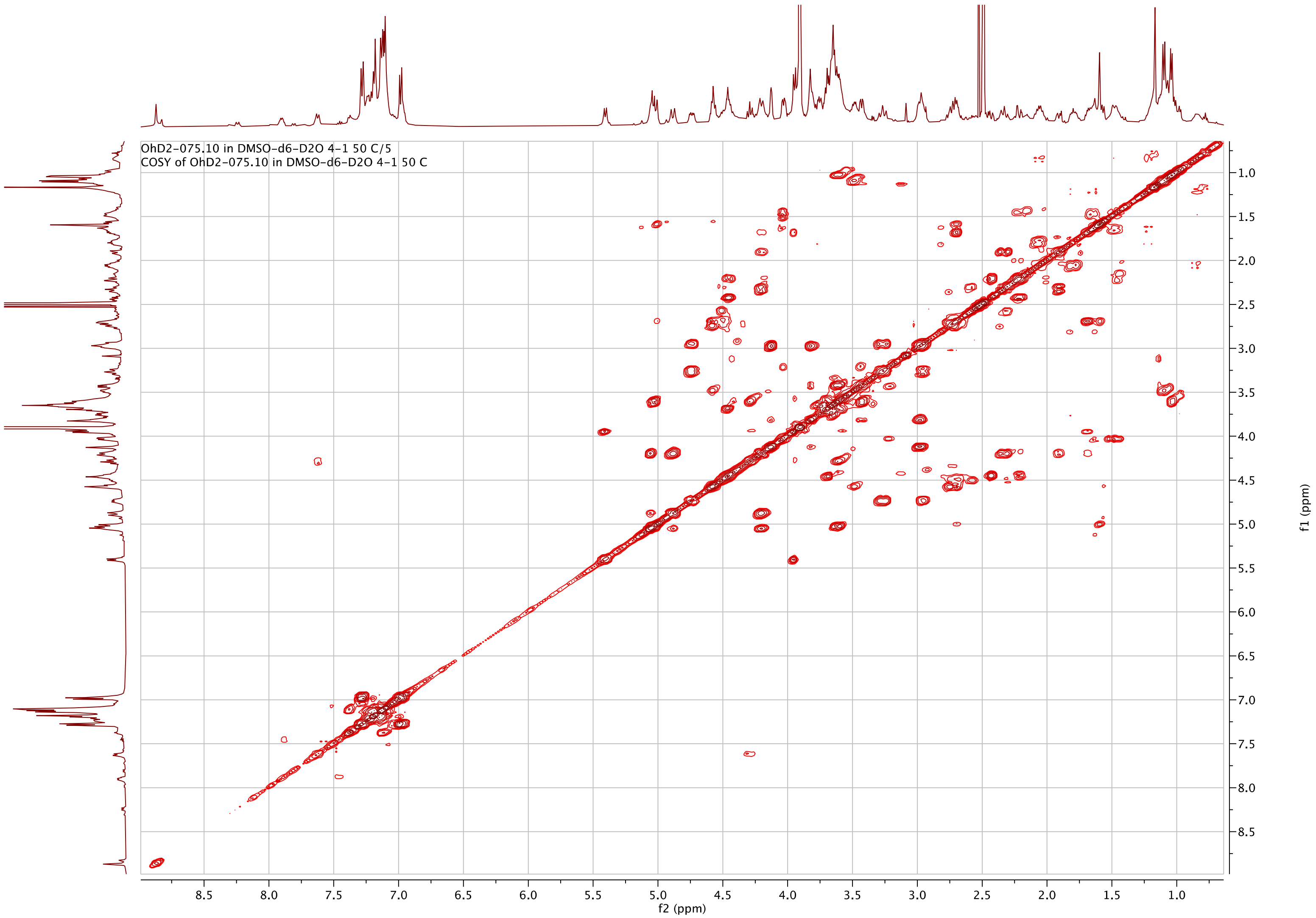

Figure S28. TOCSY spectrum of theonellamide J (**1**) in 4:1 DMSO-d_6:D_2O at 50 °C

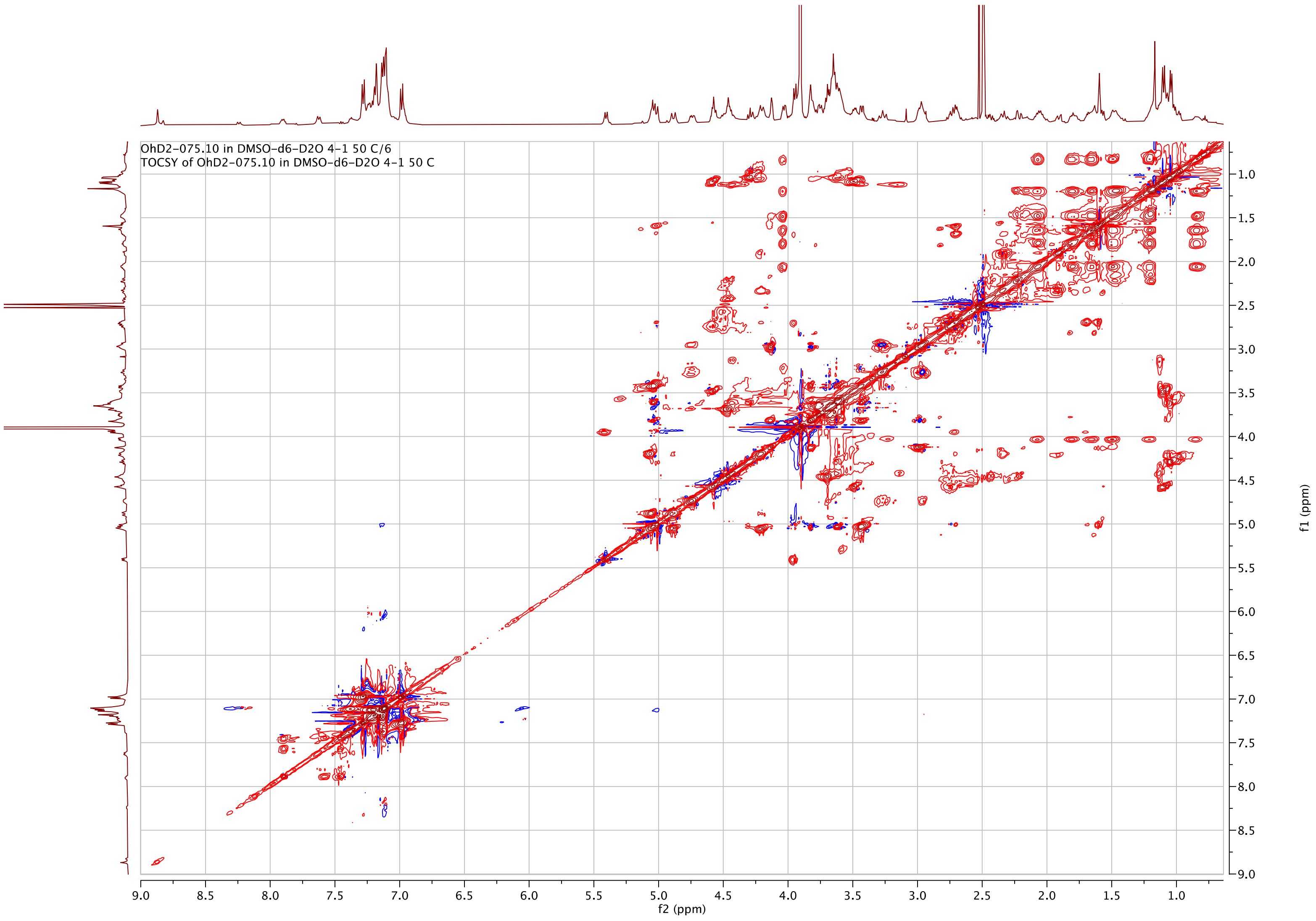

Figure S29. ROESY spectrum of theonellamide J (**1**) in 4:1 DMSO-d_6:D_2O at 50 ºC

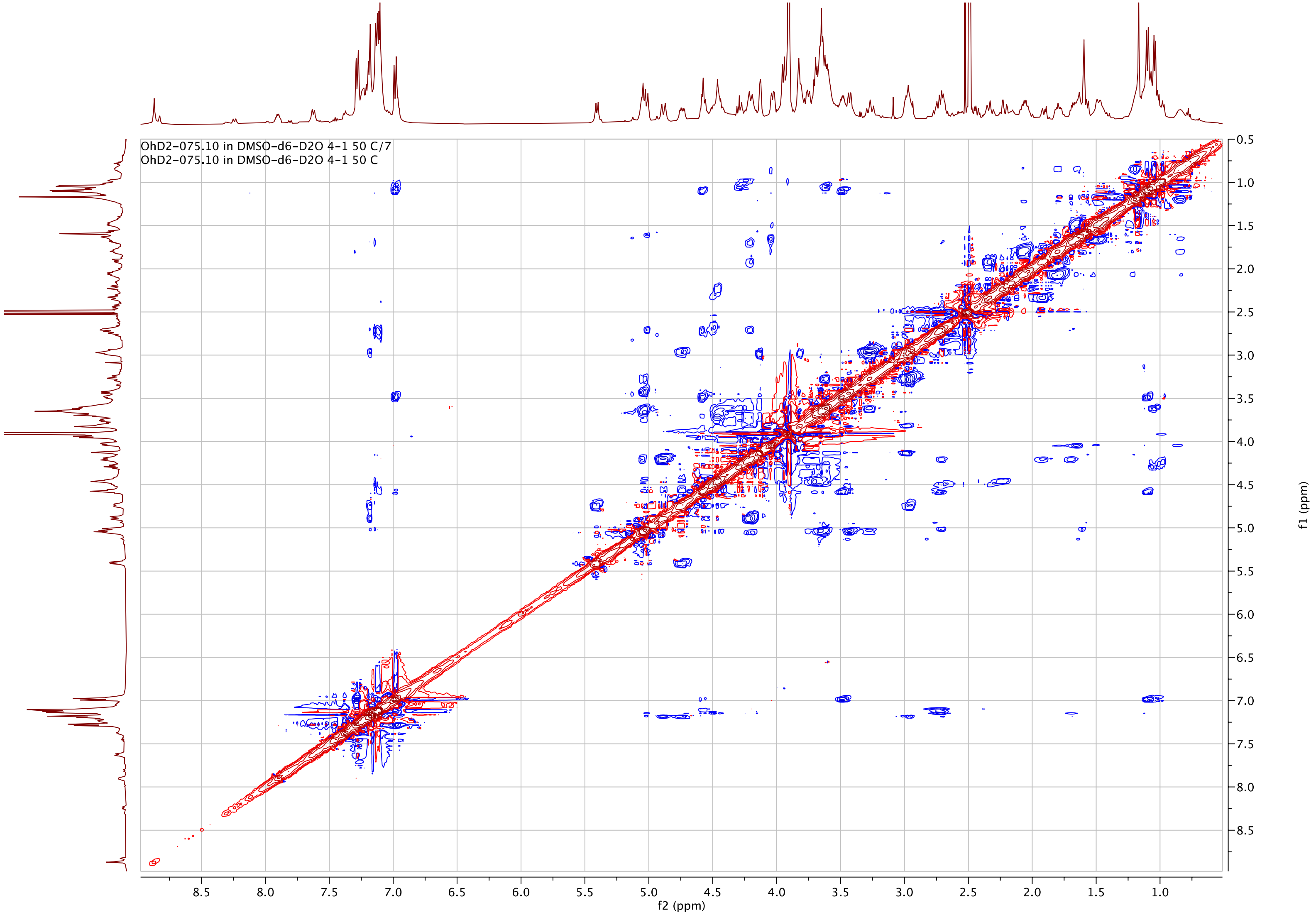

Table S4. NMR data of theonellamide J (**1**) in 4:1 DMSO-d_6:D_2O at 50 °C[a]

Position	δ_C[b]	δ_H[c]	HMBC correlations[d]	COSY correlations	TOCSY correlations	ROESY correlations[e]
Apcoa-1	172.6, C					
2a	40.4, CH_2	2.33, dd (12.8, 12.3)	Apcoa-1,3	Apcoa-2b,3	Apcoa-2b,3	Apcoa-2b
b		1.91, d (12.8)	Apcoa-1	Apcoa-2a,3	Apcoa-2a,3	Apcoa-2a,3
3	48.3, CH	4.20, m		Apcoa-2a,2b,4	Apcoa-2a,2b, 8	Apcoa-2b,4,8,10,10’
4	62.1, CH	1.69, m		Apcoa-3,5,8	Apcoa-5,6-Me,7,8	Apcoa-3,8,10,10’
5	78.9, CH	3.95, m	Apcoa-3,6,6-Me,7	Apcoa-4,6-Me	Apcoa-4,6-Me,7,8	
6	142.4, C					
6-Me	14.2, CH_3	1.59, brs	Apcoa-5,6, 7	Apcoa-7,8	Apcoa-4,5,7, 8	Apcoa-7, Phe-7
7	129.4, C	5.01, brs	Apcoa-4,5,6,6-Me,8	Apcoa-6-Me,7	Apcoa-4,5,6-Me,8	Apcoa-6-Me,8,10, 10’
8	50.7, CH	2.70, m		Apcoa-4,6-Me,7	Apcoa-3,4,5,6-Me,7	Apcoa-3,4,7,10,10’
9	145.7, C					
10,10’	129.1, CHx2	7.14, d (7.8)	Apcoa-8,10’,10,12			Apcoa-3,4,7,8
11,11’	129.2, CHx2	7.20, t (7.8)	Apcoa-9,11’,11	Apcoa-12		
12	127.2, CH	7.12, m		Apcoa-11,11’		
Ser[1]-1	172.4, C					
2	56.6, CH	3.71, dd (6.6,5.9)	Ser[1]-1	Ser[1]-3		
3	62.0, CH_2	3.62 m 2H	Ser[1]-1,2	Ser[1]-2		
sAla-1	169.8, C					
2	51.2, CH	5.06, m	sAla-1, Ser[1]-1	sAla-3a,3b	sAla-3a,3b	sAla-3b
3a	50.6, CH_2	4.88, d (14.0)	sAla-1	sAla-2,3b	sAla-2,3b	sAla-2,3b
b		4.20, m	sAla-1	sAla-2,3a	sAla-2,3a	sAla-2,3a, sHis-7
Asn-1	171.4, C					
2	52.0, CH	4.46, brm		Asn-3a,3b		Asn-3a,3b

3a	37.0, CH_2	2.45, dd (15.8,9.8)	Asn-2,4	Asn-2,3b	Asn-2,3b	Asn-2
b		2.21, dd (15.8,3.2)	Asn-1	Asn-2,3a	Asn-2,3a	Asn-2
4	172.1, C					
Han-1	171.3, C					
2	54.4, CH	5.41, d (8.6)	Han-1,3,4	Han-3	Han-3	Han-3, sHis-2, Thr-4
3	72.0, CH	3.95, d (8.6)	Han-1,2,4	Han-2	Han-2	Han-2
4	174.1, C					
BrMePhe-1	171.8, C					
2	59.2, CH	4.58, m	BrMePhe-1, 3-Me,4	BrMePhe-3	BrMePhe-3, 3-Me	BrMePh-3,3-Me,5,5
3	39.0, CH	3.49, dq (4.4,7.2)	BrMePhe-2,3-Me,4, 5,5’	BrMePhe-2, 3-Me	BrMePhe-2, 3-Me	BrMePhe-2,3-Me,5,5’
3-Me	18.0, CH_3	1.10, d (7.2)	BrMePhe-2, 3,4	BrMePhe-3	BrMePhe-2,3	BrMePhe-2, 3,5,5’
4	141.3, C					
5,5’	131.3, CHx2	6.99, d (8.3)	BrMePhe-3,5’,5,7	BrMePhe-6,6’		BrMePhe-2, 3,3-Me
6,6’	131.8, CHx2	7.28, d (8.3)	BrMePhe-4, 6’,6,7	BrMePhe-5,5’		
7	120.8, C					
iSer-1	172.6, C					
2	69.8, CH	4.13, brs	iSer-1	iSer-3a,3b	iSer-3a,3b	iSer-3b
3a	43.9, CH_2	3.83, m	iSer-2	iSer-2,3b	iSer-2,3b	iSer-3b, Gal-3,5
b		2.98, m	iSer-1,2	iSer-2,3a	iSer-2,3a	iSer-2,3a
Ada-1	174.6, C					
2	52.5, CH	4.03, dd (9.7,3.5)	Ada-1,3, iSer-1	Ada-3a,3b	Ada-3a,3b,4a,4b,5a,5b	Ada-3a,3b, 4b
3a	31.3, CH_2	1.65, m		Ada-2,3b,4a, 4b	Ada-2,3b,4a,4b,5a,5b	Ada-2, 3b
b		1.49, m		Ada-2,3a,4a, 4b	Ada-2,3a,4a, 4b,5a,5b	Ada-2, 3a
4a	21.8, CH_2	1.20, m		Ada-3a,3b,4b,5a,5b	Ada-2,3a,3b,4b,5a,5b	Ada-3b
b		0.83, m		Ada-3a,3b,4a,5a,5b	Ada-2,3a,3b,4a,5a,5b	Ada-2,3a

5a	35.2, CH_2	2.06, m	Ada-4,6	Ada-4a,4b,5b	Ada-2,3a,3b,4a,4b,5b	Ada-5b
b		1.79, m	Ada-3,4,6	Ada-4a,4b,5a	Ada-2,3a,3b,4a,4b,5a	Ada-5a
6	173.7, C					
sHis-1	170.9, C					
2	54.4, CH	4.74, dd (12.2,4.6)	sHis-3	sHis-3a,3b	sHis-3a,3b	Has-2, sHis-3b,7
3a	26.5, CH_2	3.27, brt (13.5)	sHis-2,4,7	sHis-2,3b	sHis-2,3b	sHis-3b Gal-1,2
b		2.97, m	sHis-1,2,4	sHis-2,3a	sHis-2,3a	sHis-2,3a,7
4	132.0, C					
6	137.2, CH	8.88, s[f]				
8	124.3, CH	7.18, s	sHis-4,6		sHis-3a,3b	sHis-2,3b, sAla-3a
Thr-1	172.6, C					
2	58.7, CH	4.29, t (9.5)	Thr-1,3,4, sHis-1	Thr-3	Thr-3,4	Thr-3,4
3	68.9, CH	3.61, m	Thr-2	Thr-2,4	Thr-2,4	Thr-3,4, sHis-3a
4	21.7, CH_3	1.04, d (6.1)	Thr-3	Thr-3	Thr-2,3	Has-2, Thr-2,3
Ser^2-1	170.0, C					
2	56.5, CH	4.46, m	Ser^2-1,3, Thr-1	Ser^2-3	Ser^2-3	Ser^2-3
3	62.0, CH_2	3.69, m	Ser^2-1	Ser^2-2	Ser^2-2	Ser^2-2
Phe-1	171.3, C					
2	55.2, CH	4.57, m	Phe-3, Ser^2-1	Phe-3a,3b	Phe-3a,3b	Phe-3b,5,5’
3a	39.2, CH_2	2.76, m	Phe-1,2,4, 5,5’	Phe-2	Phe-2,3b	Phe-5,5’
b		2.72, m	Phe-2,4,5, 5’	Phe-2	Phe-2,3a	Phe-2,5,5’
4	137.0, C					
5,5’	129.8, CHx2	7.11, m	Phe-6,6’,7			Phe-2,3a,3b
6,6’	128.5, CHx2	7.12, m	Phe-4			Ser^2-2
7	127.6, CH	7.13, m				Apcoa-6-Me
Gal-1	89.0, CH	5.03, d (8.9)	sHis-4,6, Gal-4	Gal-2	Gal-2,3,4	Gal-2,3, sHis-3a
2	69.5, CH	3.62, m	Gal-1,3	Gal-1,3	Gal-1,3	
3	73.7, CH	3.43, dd (9.2,2.9)		Gal-2,4	Gal-1,2,4	Gal-1,4
4	69.8, CH	3.83, m	Gal-3	Gal-3,5	Gal-1,2,3,5,6	

5	79.0, CH	3.66, m	Gal-2,6	Gal-4
6	62.0, CH_2	3.75, m	Gal-5	Gal-4
		3.58, m		

[a] ^{1}H (500 MHz), ^{13}C (125 MHz) and ^{15}N (50 MHz). [b] Multiplicity and assignment from HSQC experiment. [c] Multiplicity (*J* in Hz). [d] HMBC correlations, optimized for 8 Hz. [e] Selected NOEs from ROESY experiment. [f] Exchanged with D_2O.

Figure S30. ^{1}H NMR spectrum (500 MHz) of theonellamide J (**1**) in DMF-d_7

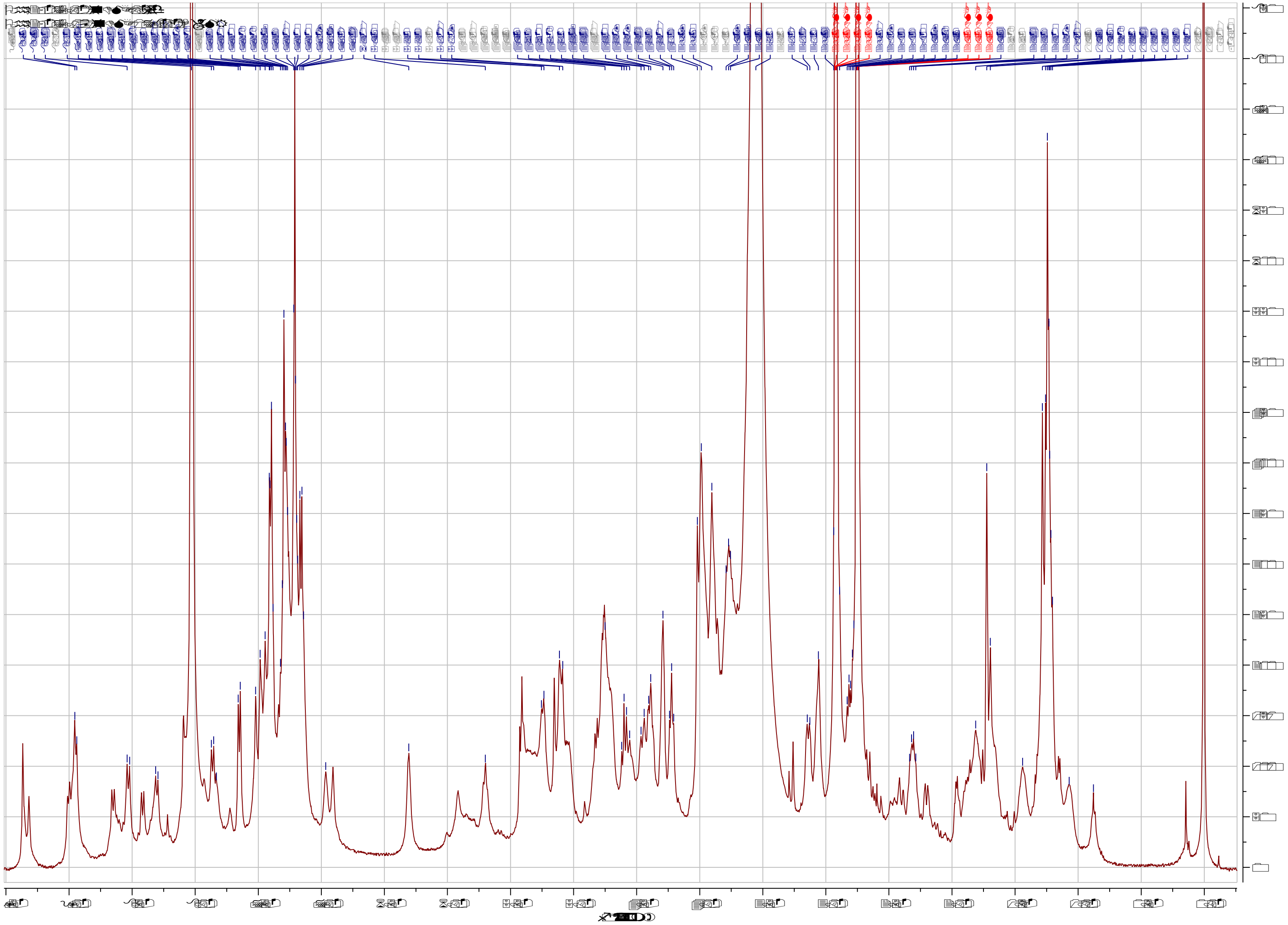

Figure S31. ^{13}C NMR spectrum (125 MHz) of theonellamide J (**1**) in DMF-d_7

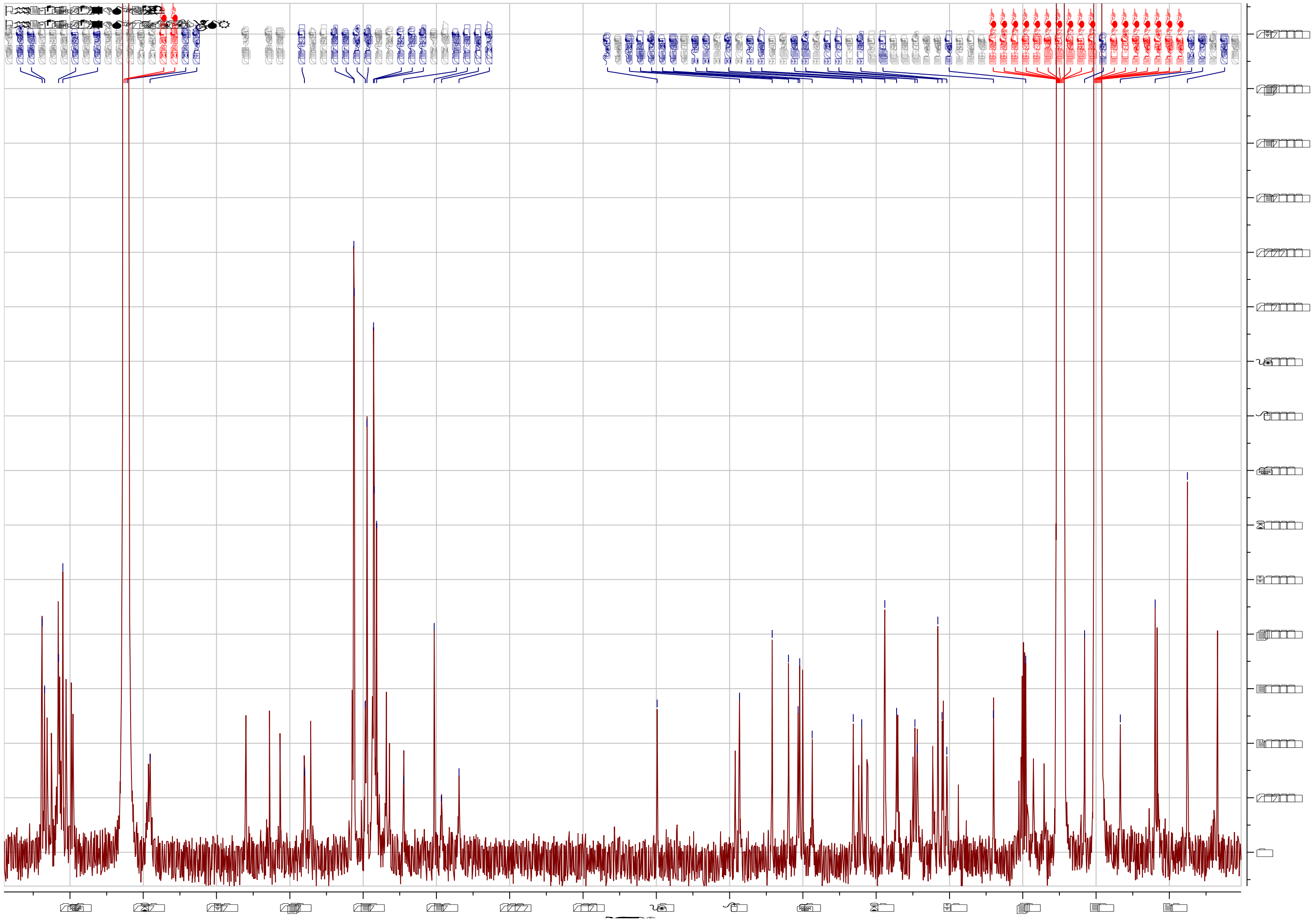

Figure S32. HSQC spectrum of theonellamide J (**1**) in DMF-d_7

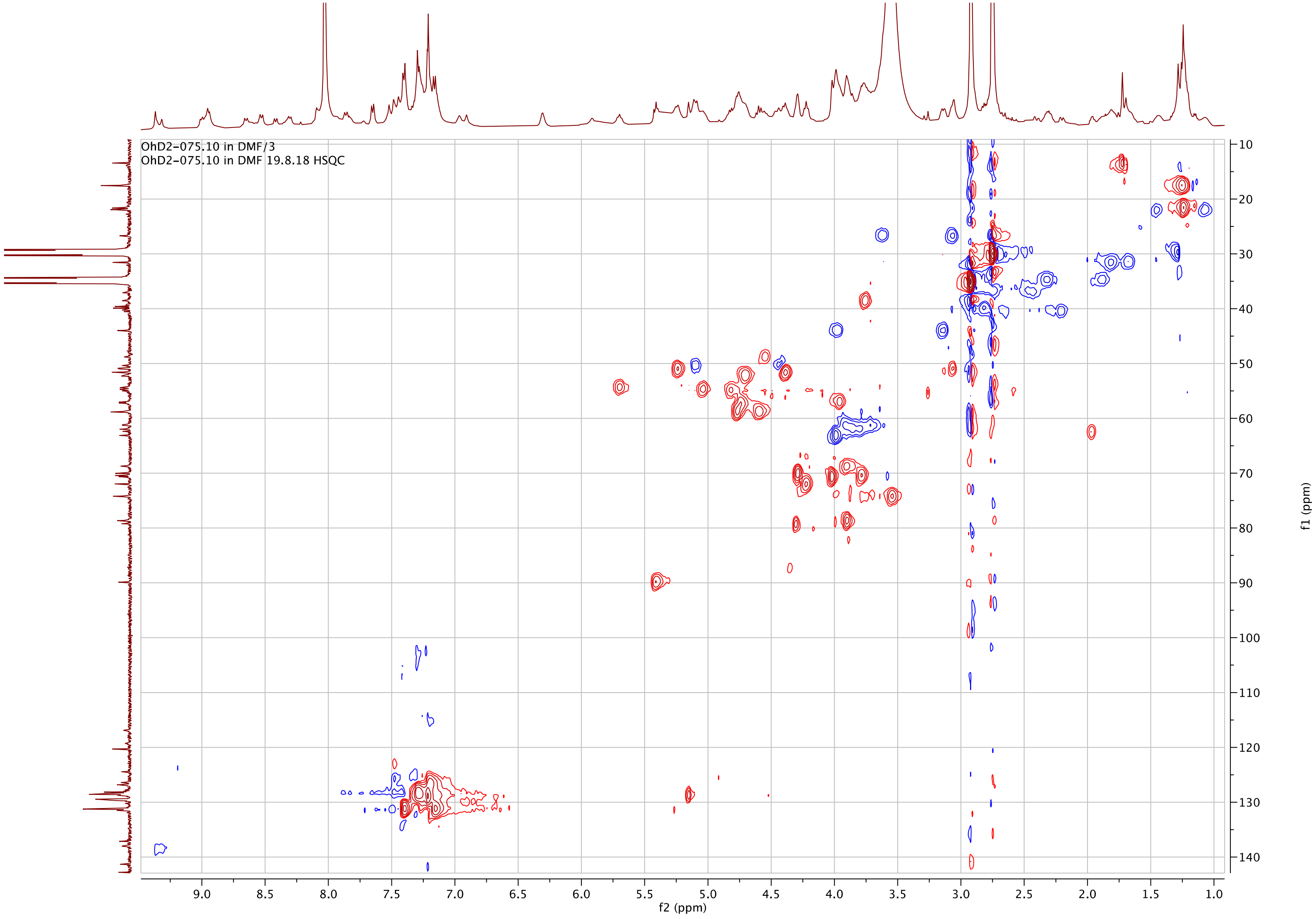

Figure S33. HMBC spectrum of theonellamide J (**1**) in DMF-d_7

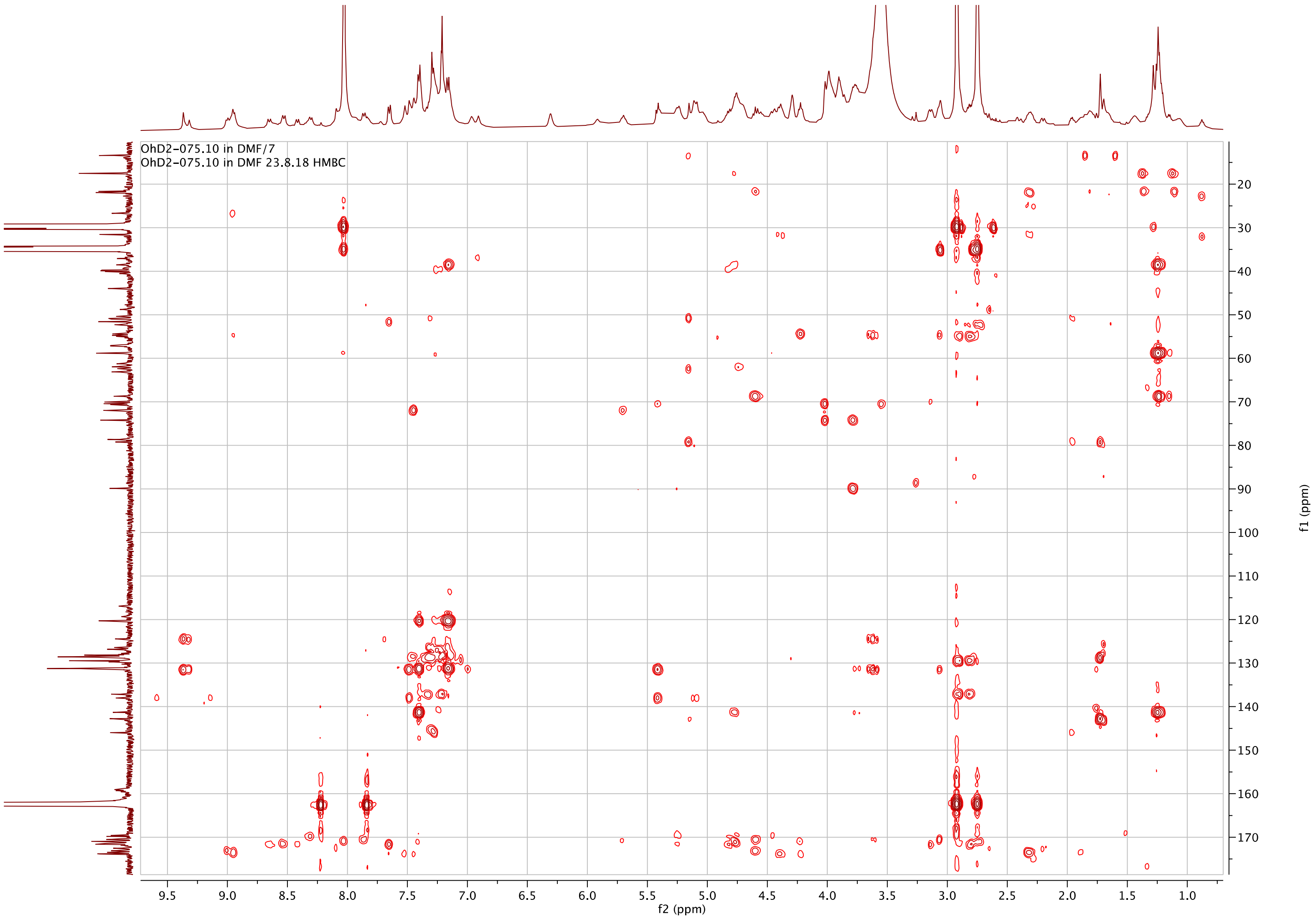

Figure S34. COSY spectrum of theonellamide J (**1**) in DMF-d_7

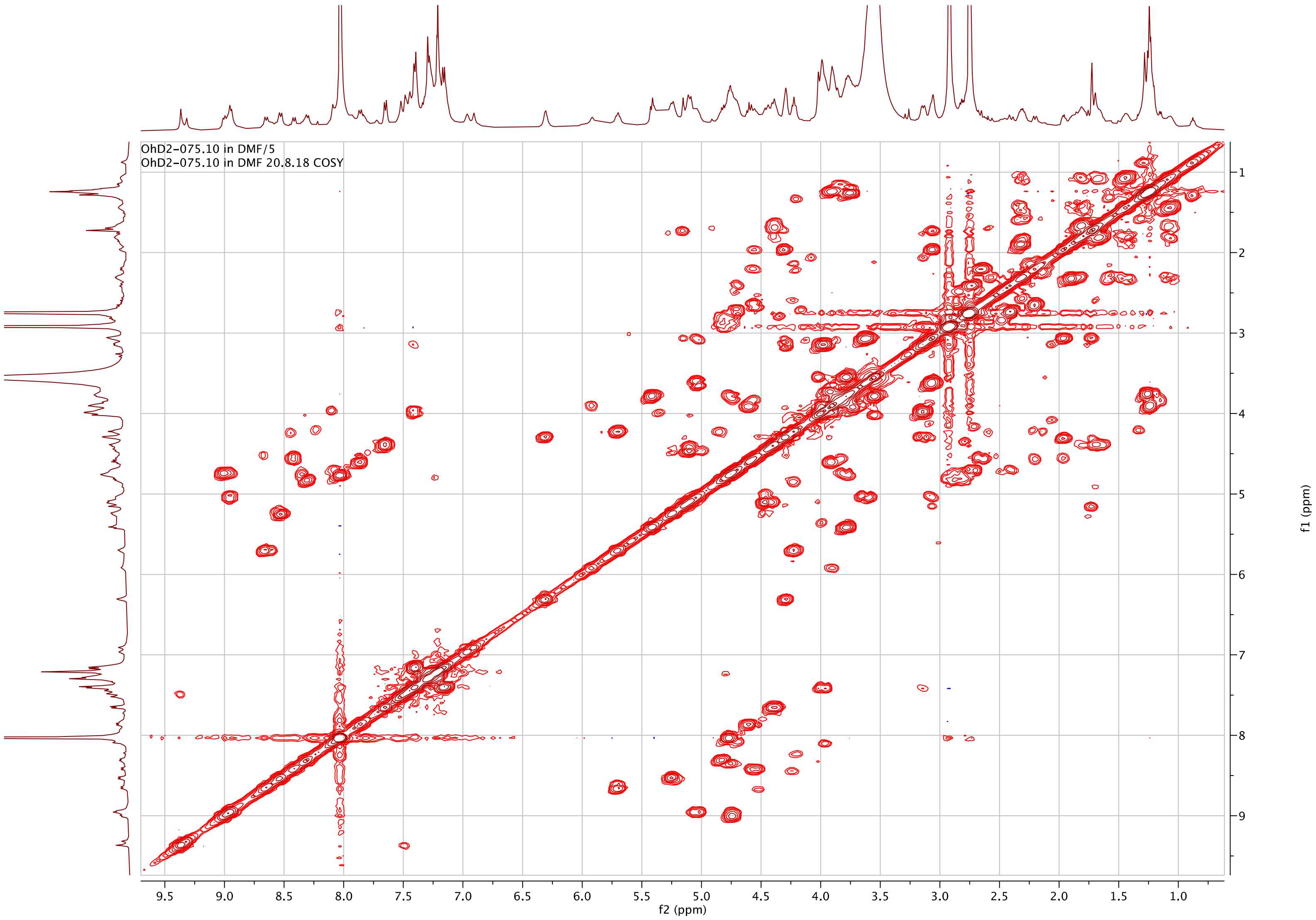

Figure S35. TOCSY spectrum of theonellamide J (**1**) in DMF-d_7

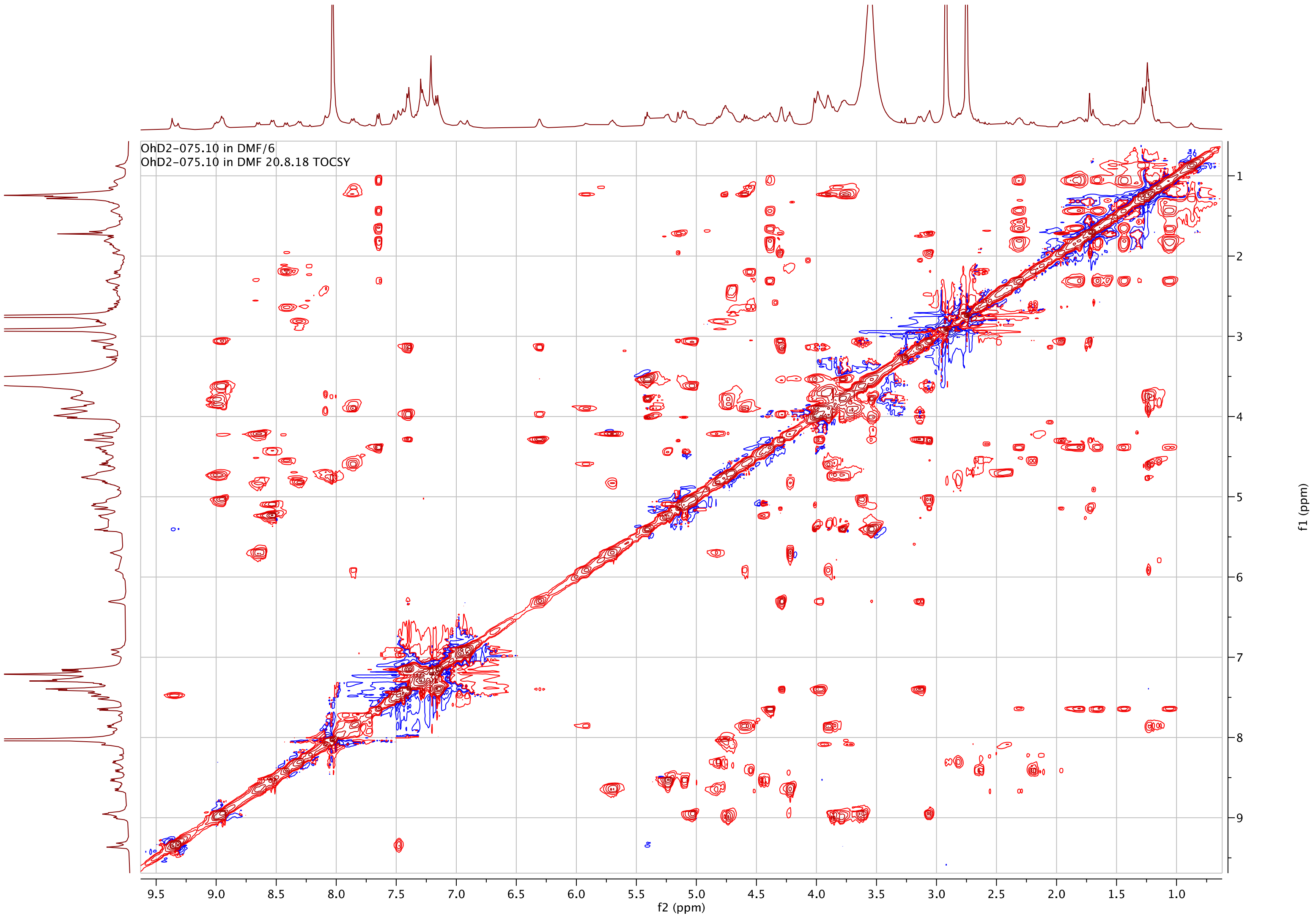

Figure S36. ROESY spectrum of theonellamide J (**1**) in DMF-d_7

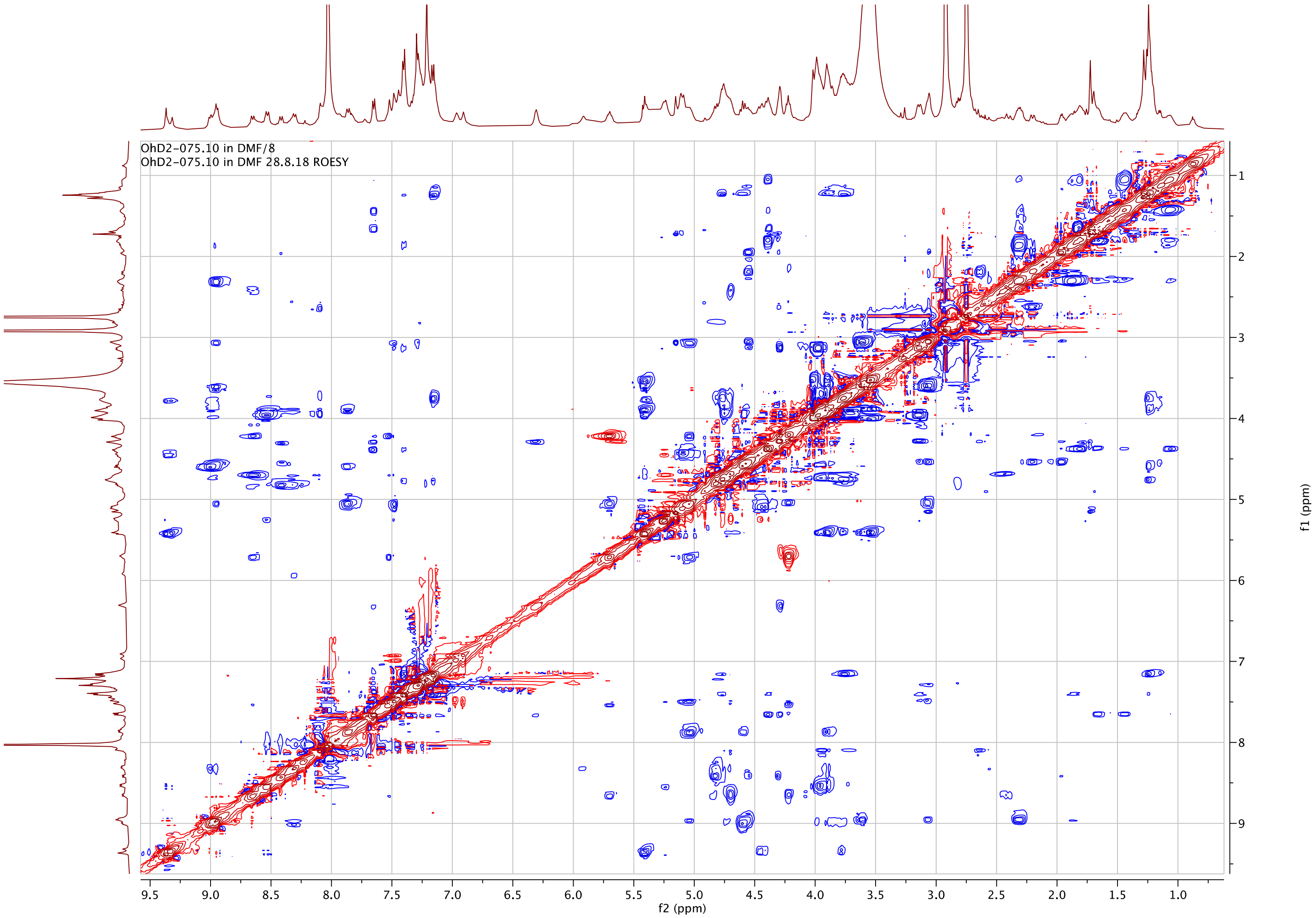

Figure S37. N-H HSQC spectrum theonellamide J (**1**) in DMF-d_7

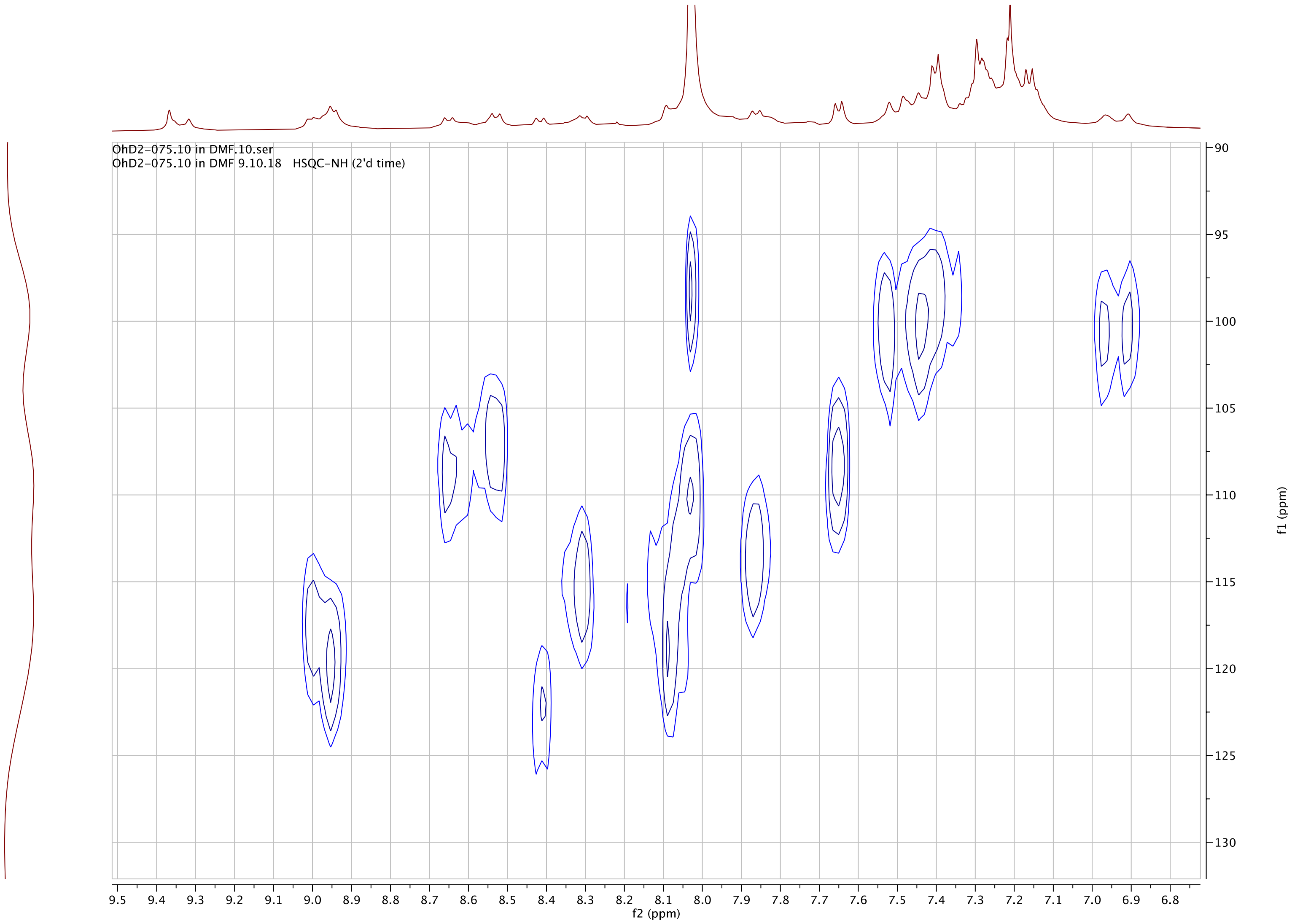

Table S5. NMR data of theonellamide J (**1**) in DMF-d_7[a]

Position	δ_C[b]	δ_H[c]	HMBC correlations[d]	COSY correlations	TOCSY correlations	ROESY correlations[e]
Apcoa-1	172.5, C					
2a	40.6, CH_2	2.66, t (12.5)	Apcoa-1,3	Apcoa-2b,3	Apcoa-2b,3,3-NH	Apcoa-2b,3-NH, Ser[1]-2-NH
b		2.20, dd (12.5,3.3)	Apcoa-1,3	Apcoa-2a,3	Apcoa-2a,3,3-NH	Apcoa-2a,3,4,8
3	48.8, CH	4.56, m		Apcoa-2a,2b,3-NH,4	Apcoa-2a,2b,3-NH,4,8	Apcoa-2b,3-NH,4,8,10,10’
3-NH	*122.3, NH*	8.42, d (9.4)	Phe-1	Apcoa-3	Apcoa-2a,2b,3,4	Apcoa-2a,3,4,5, Phe-2
4	62.4, CH	1.96, dt (5.5,5.8)	Apcoa-5,8,9	Apcoa-3,5,8	Apcoa-2a, 2b,3,3-NH, 5,6-Me,7,8	Apcoa-2b,3,3-NH,5,6-Me, 7,8,10,10’
5	79.2, CH	4.30, brs ($\nu_{1/2}$=12.9 Hz)	Apcoa-6,7	Apcoa-4,6-Me	Apcoa-4, 6-Me,7,8	Apcoa-3-NH,4,6-Me
6	142.8, C					
6-Me	13.4, CH_3	1.72, s	Apcoa-5,6,7	Apcoa-5,7,8	Apcoa-4,5,7,8	Apcoa-4,5,7, Phe-5,5’
7	128.8, CH	5.15, brs	Apcoa-4,5,6,6-Me,8	Apcoa-6-Me,8		Apcoa-4,6-Me,8,10,10’
8	50.9, CH	3.06, brs ($\nu_{1/2}$~9 Hz)	Apcoa-4,6,9	Apcoa-4,7	Apcoa-4,5,6-Me	Apcoa-2b,3,4,7,10,10’
9	146.0, C					
10,10’	128.6, CH x 2	7.29, m	Apcoa-10’,10, 11,11'			Apcoa-3,4,7,8
11,11’	128.2, CH x 2	7.21, m	Apcoa-9,11’, 11,12			
12	126.4, CH	7.19, m				
Ser[1]-1	171.4, C					

2	57.0, CH	3.96, m		Ser^{1}-2-NH,3a,3b	Ser^{1}-2-NH	Ser^{1}-3b, Ser^{2}-2, sAla-2,2-NH
2-NH	*114.6, NH*	8.09, s	Apcoa-1	Ser^{1}-2	Ser^{1}-2, 3a,3b	Ser^{1}-3a,3b
3	61.2, CH_2	3.91, m		Ser^{1}-2,3b	Ser^{1}-2-NH	Ser^{1}-2-NH Ser^{1}-2-NH, sAla-2-NH
		3.71, m	Ser^{1}-1	Ser^{1}-3,3a	Ser^{1}-2-NH	
sAla-1	169.6, C					
2	51.0, CH	5.25, brd (9.9)	sAla-1	sAla-2-NH	sAla-2-NH, 3a,3b	sAla-2-NH, 3b(neg)
2-NH	*107.0, NH*	8.53, d (9.9)	Ser^{1}-1, sAla-2	sAla-2	sAla-2-NH, 3a,3b	sAla-2, sHis-6, Ser^{1}-2,3b, Thr-3
3a	50.4, CH_2	5.11, brd (12.1)	sAla-2, sHis-6	sAla-3b	sAla-2,2-NH,3b	sAla-3b, sHis-3b,8 sHis-6,
b		4.45, brd (12.1)	sAla-1	sAla-3a	sAla-2,2-NH,3a	sAla-2(neg),3a, Ser^{1}-2
Asn-1	171.0, C					
2	52.3, CH	4.71, brm		Asn-2-NH,3a,3b		Has-2-NH
2-NH	*118.9, NH*	8.08, m		Asn-2	Asn-2-NH,3a,3b	
3a	37.1, CH_2	2.73, m	Asn-1,2	Asn-2,3b	Asn-2,2-NH,3b	
b		2.41, dd (15.4,2.8)		Asn—2,3a	Asn-2,2-NH,3a	
4	173.9, C					
4-NH_2	*100.6, NH_2*	6.97, s				
		7.45, s	Asn-4			
Han-1	170.9, C					
2	54.4, CH	5.71, brdd (8.6,7.8)	Han-3	Han-2-NH,3	Han-2-NH, ,3-OH	sHis-2, Hans-3(neg), BrMePhe-2-NH, Thr-4
2-NH	*108.3, NH*	8.65, brd (8.6)		Han-2	Han-2,3,3-OH	Han-2,3, Asn-2,
3	72.0, CH	4.23, brt (7.8)	Han-1,2, 4	Han-2,3-OH	Han-2,2-NH,3,3-OH	sHis-2, Han-2(neg)
3-OH		4.83, m				
4	173.8, C					

4-NH_2	*100.4, NH_2*	6.91, s<br>7.52, s	 Han-3,4			Han-2,3
BrMePhe-1	171.0, C					
2	58.8, CH	4.77, m	BrMePhe-1,3,3-Me,4	BrMePhe-2-NH,3	BrMePhe-2-NH,3,3-Me	BrMePhe-3,3-Me
2-NH	*110.3, NH*	8.03, m	Han-1, BrMePhe-2	BrMePhe-2	BrMePhe-2,3,3-Me	Has-2,3 BrMePhe-2,3
3	38.6, CH	3.76, m	BrMePhe-3-Me,4, 5,5’	BrMePhe-2,3-Me	BrMePhe-2,2-NH,3-Me	BrMePhe-2,3-Me
3-Me	17.5, CH_3	1.25, d (7.1)	BrMePhe-1,2,3,4	BrMePhe-3	BrMePhe-2,3	
4	141.3, C					
5,5’	131.2, CH x 2	7.17, d x 2 (8.0)	BrMePhe-3,5’,5,6,6’,7	BrMePhe-6,6’		BrMePhe-3,3-Me
6,6’	131.3, CH x 2	7.41, d x 2 (8.0)	BrMePhe-4,5,5’,6’,6,7	BrMePhe-5,5’		
7	120.3, C					
iSer-1	171.6, C					
2	70.0, CH	4.29, brs		iSer-2-OH,3a,3b	iSer-3-NH	iSer-2-OH,3a, 3b, Ada-2-NH
2-OH		6.31, brs		iSer-2	iSer-2,3a, 3b	iSer-2
3a	44.0, CH_2	3.98, m		iSer-2,3b	iSer-2,2-OH,3b,3-NH	iSer-2,3b,
b		3.14, brd (11.3)	iSer-1,2	iSer-2,3a	iSer-2,2-OH,3a,3-NH	iSer-2,3a,
3-NH	*98.9, NH*	7.42, m	BrMePhe-1	iSer-3a	iSer-2,3a, 3b	Ada-2,2-NH, iSer-3b BrMePhe-2,3-Me
Ada-1	173.8, C					
2	51.6, CH	4.39, ddd (12.2, 8.7, 2.8)	Ada-1,3	Ada-2-NH,3a, 3b	Ada-2-NH, 3a,3b,4a, 4b,5a,5b	Ada-3a, 3b,4a,4b
2-NH	*108.2, NH*	7.65, d (8.7)	iSer-1, Ada-1,2,3	Ada-2	Ada-2, 3a,3b,4a, 4b,5a,5b	iSer-2,3-NH, Ada-2,3b,4a,
3a	31.6, CH_2	1.81, m		Ada-2,3b, 4a,4b	Ada-2,2-NH,3b, 4a,4b, 5a,5b	

b		1.68, m	Ada-2	Ada-2,3a, 4a,4b	Ada-2,2-NH,3a, 4a,4b, 5a,5b	
4a	21.9, CH_2	1.44, m		Ada-3a, 3b,4b,5a,5b	Ada-2,2-NH,3a,3b, 4b,5a,5b	
b		1.07, m		Ada-3a, 3b,4a,5a,5b	Ada-2,2-NH,3a,3b, 4a,5a,5b	
5a	34.8, CH_2	2.33 m	Ada-3,4,6	Ada-4a,4b, 5b	Ada-2,2-NH,3a,3b, 4a,4b,5b	
b		1.90, m	Ada-3,6	Ada-4a,4b, 5a	Ada-2,2-NH,3a,3b, 4a,4b,5a	
6	173.5, C					
sHis-1	170.5, C					
2	54.7, CH	5.04, brm		sHis-2-NH,3a,3b	sHis-2-NH,3a,3b	sHis-2-NH,3b Han-2,3
2-NH	*119.8, NH*	8.95, d (7.1)	Ada-6, sHis-2,3	sHis-2	sHis-2,3a,3b	sHis-2,3a,3b,
3a	26.7, CH_2	3.63, m	sHis-1,2,4,8	sHis-2,3b	sHis-2,2-NH,3b	sHis-2-NH,3b
b		3.08, m	sHis-1,2,4	sHis-2,3a	sHis-2,3a	sHis-2,2-NH,3a,
4	131.5, C					
6	138.0, CH	9.37, s	5.11, sHis-4,8	sHis-8	sHis-8	Gal-1,2, sAla-2-NH, 3b Ser^2-2
8	124.5, CH	7.48, brs	sHis-4,6	sHis-6	sHis-6	Gal-1, sHis-3b,
Thr-1	173.1, C					
2	58.8, CH	4.60, t (9.4)	sHis-1, Thr-1,3,4	Thr-2-NH,3	Thr-2-NH,3,3-OH,4	Ser^2-2-NH, sHis-3b Thr-2-NH,4
2-NH	*113.7, NH*	7.86, d (9.6)	sHis-1	Thr-2	Thr-2,3,3-OH,4	sHis-2, Thr-2,3,4
3	68.7, CH	3.91, m		Thr-2,3-OH,4	Thr-2,2-NH,3-OH,4	Gal-1, Ser^2-2, sAla-2-NH, Thr-2,2-NH,4
3-OH		5.91, brs		Thr-3	Thr-2,2-NH,3,4	Thr-4, Phe-2-NH
4	21.7, CH_3	1.24, d (6.1)	Thr-2,3	Thr-3	Thr-2,2-NH,3,3-OH	Thr-2,2-NH,3, Ser^2-2, Han-2

Ser^2-1	169.8, C					
2	57.2, CH	4.75, m	Ser^2-1,3	Ser^2-2-NH	Ser^2-2-NH, 3a,3b,3-OH	sHis-6 Ser^2-2-NH, 3a, Ser^1-2, Phe-2-NH
2-NH	*117.9, NH*	9.00, d (7.5)	Thr-1	Ser^2-2,	Ser^2-2,3a, 3b,3-OH	Phe-2-NH, Ser^2-2,3a, 3b, Thr-2
3a	62.0, CH_2	3.86, m	Ser^2-1		Ser^2-3-OH	Ser^2-2-NH
b		3.80, m			Ser^2-3-OH	
3-OH		5.32, m			Ser^2-2,2-NH,3a,3b	
Phe-1	171.6, C					
2	55.0, CH	4.82, m	Phe-3	Phe-2-NH, 3a,3b	Phe-2-NH,3a,3b	Apcoa-3-NH, Phe-3a, 3b,5,5’
2-NH	*115.2, NH*	8.30, d (9.6)	Ser^2-1	Phe-2	Phe-2,3a,3b	Ser^2-2,2-NH, Thr-3-OH, Phe-3a,3b
3a	40.2, CH_2	2.91, m	Phe-1,2,4,	Phe-2,3b	Phe-2,2-NH, 3b	Phe-2,2-NH, 3b
b		2.81, dd (12.9,6.9)	Phe-1,2,4	Phe-2,3a	Phe-2,2-NH, 3a	Phe-2
4	137.2, C					
5,5’	129.5, CH x 2	7.21, m	Phe-4,5’,5, 6,6’			Phe-2, Apcoa-6-Me
6,6’	128.5, CH x 2	7.26, m	Phe-4			
7	126.8, CH	7.20, m				
Gal-1	89.9, CH	5.41, d (8.3)	sHis-4,6, Gal-2,4	Gal-2	Gal-2,3,4,5,6,6’	sHis-6, Gal-3,6a,6b
2	70.4, CH	3.78, brs	Gal1,3	Gal-1,3	Gal-1,3,4	sHis-6
3	74.2, CH	3.54, m	Gal-2,4	Gal-2,4	Gal-1,2,4,5,6,6’	Gal-1
4	70.7, CH	4.02, m	Gal-2,3,5	Gal-3	Gal-1,2,3,4,5,6,6’	
5	78.6, CH	3.90, m			Gal-4, 5,6,6’	
6	63.2, CH_2	3.99, m			Gal-4, 5,6,6’	Gal-1

[a] ^{1}H (500 MHz), ^{13}C (125 MHz) and ^{15}N (50 MHz). [b] Multiplicity and assignment from HSQC experiment. [c] Multiplicity (*J* in Hz). [d] HMBC correlations, optimized for 8 Hz. [e] Selected NOEs from ROESY experiment.

Figure S38. (+)-HRESIMS data of theonellamide J (**1**)

Elemental Composition Report

Single Mass Analysis

Tolerance = 20.0 PPM / DBE: min = -1.5, max = 400.0
Element prediction: Off
Number of isotope peaks used for i-FIT = 5

Monoisotopic Mass, Even Electron Ions
48 formula(e) evaluated with 11 results within limits (up to 3 closest results for each mass)
Elements Used:
C: 70-80 H: 90-100 N: 10-20 O: 25-30 79Br: 1-1

OHD2-075/10 Carmeli1281 59 (2.613) Cm (59:60)
Ohad Hasin
1: TOF MS ES+

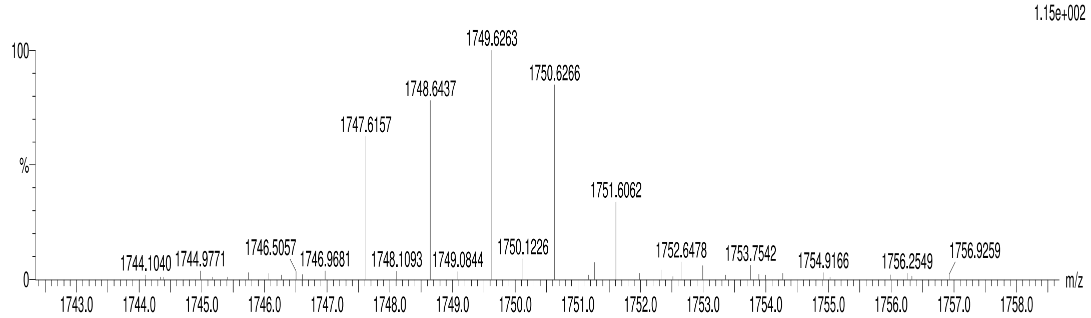

		mDa	PPM	DBE	i-FIT	i-FIT (Norm)	Formula
Minimum:				-1.5			
Maximum:		5.0	20.0	400.0			

Mass	Calc. Mass	mDa	PPM	DBE	i-FIT	i-FIT (Norm)	Formula
1747.6157	1747.6127	3.0	1.7	34.5	44.0	1.0	C76 H100 N16 O27 79Br
	1747.6199	-4.2	-2.4	30.5	44.0	1.0	C70 H100 N20 O28 79Br
	1747.6087	7.0	4.0	30.5	44.4	1.4	C71 H100 N18 O29 79Br

Figure S39. ^{1}H NMR spectrum (500 MHz) of 5-*cis*-Apoa-theopalauamide (**2**) in 4:1 DMSO-d_6:H_2O at 50 ^{o}C

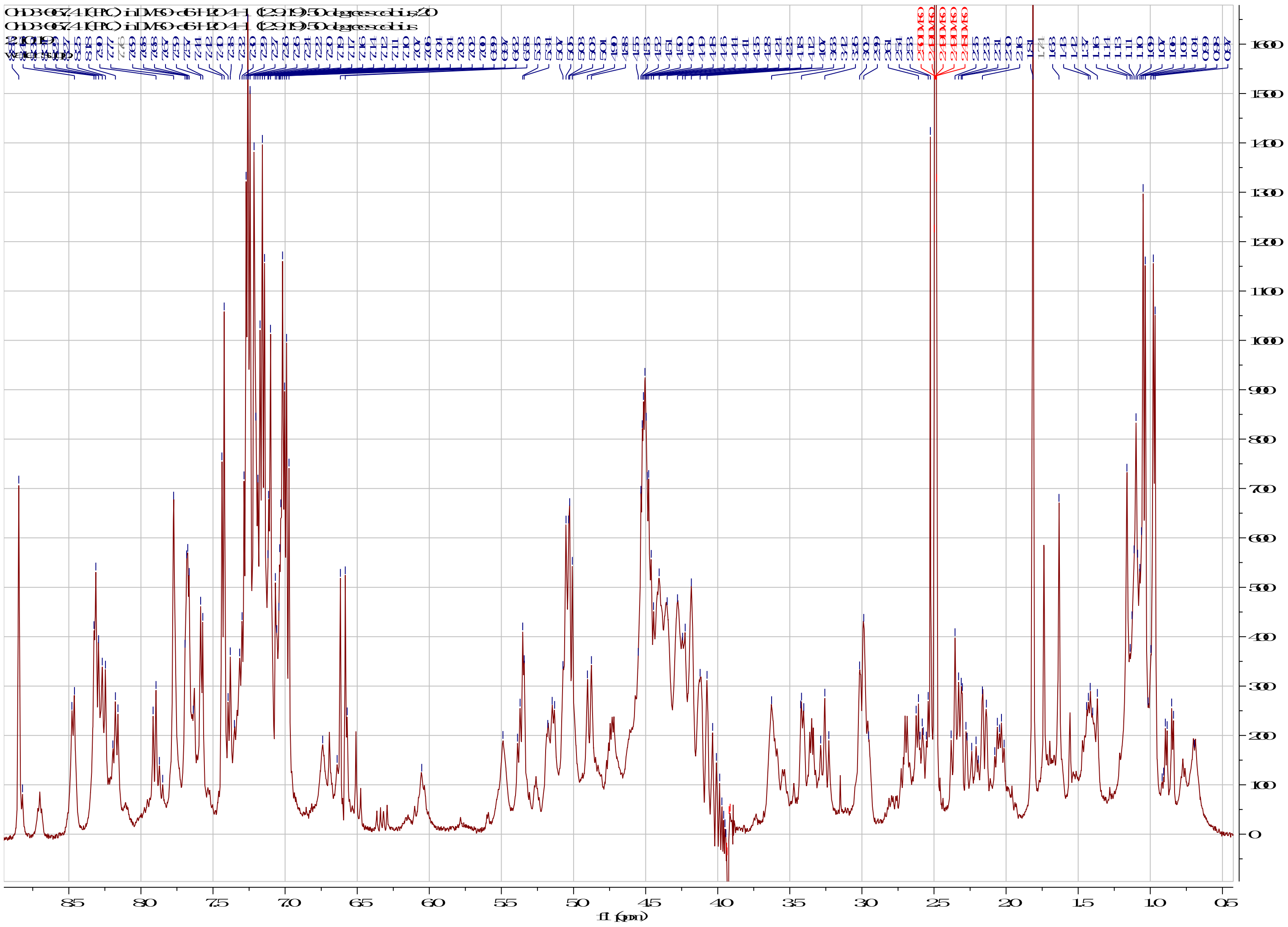

Figure S40. ^{13}C NMR spectrum (125 MHz) of 5-*cis*-Apoa-theopalauamide (**2**) in 4:1 DMSO-d_6:H_2O at 50 ^{o}C

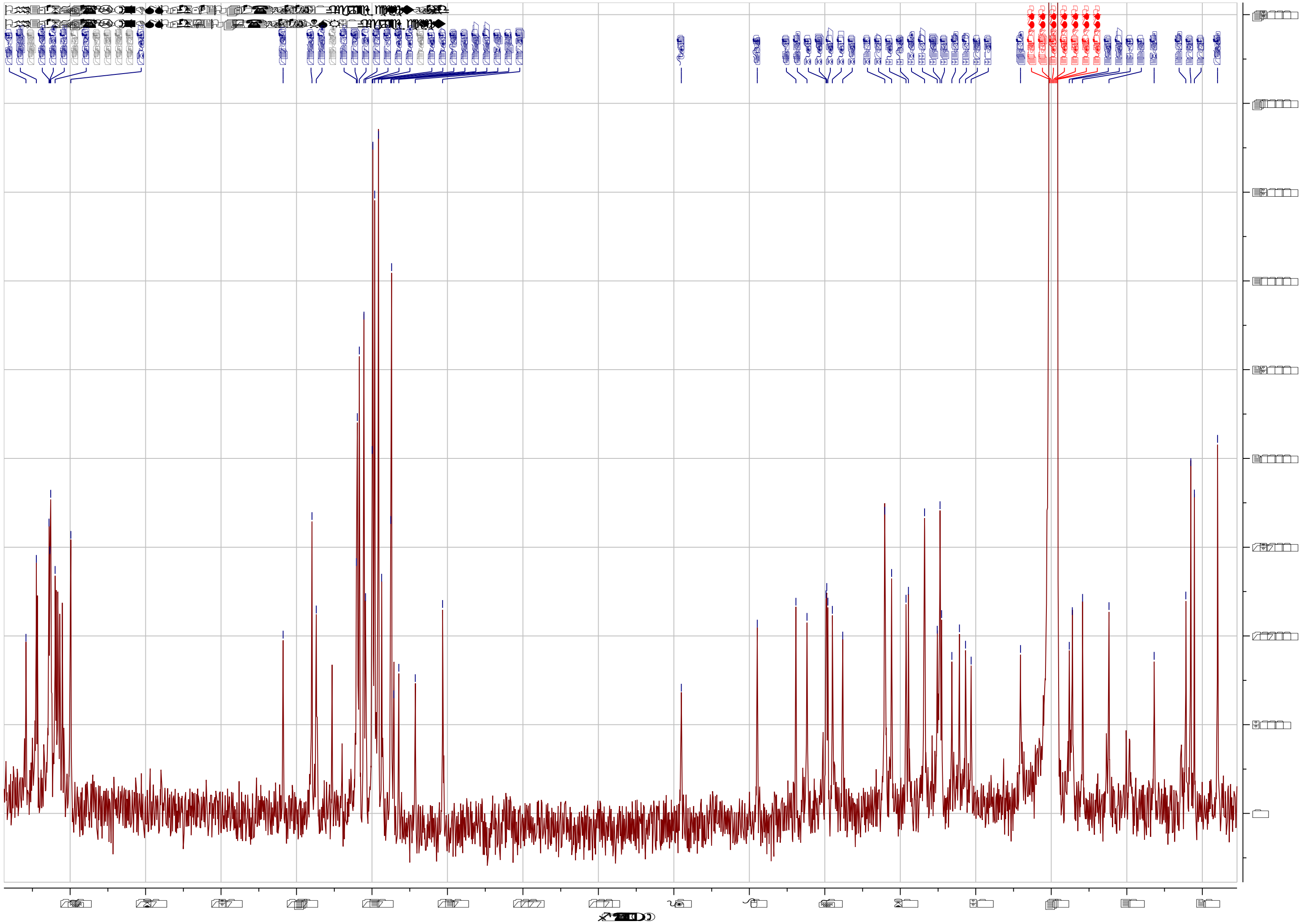

Figure S41. HSQC spectrum of 5-*cis*-Apoa-theopalauamide (**2**) in 4:1 DMSO-d_6:H_2O at 50 ºC

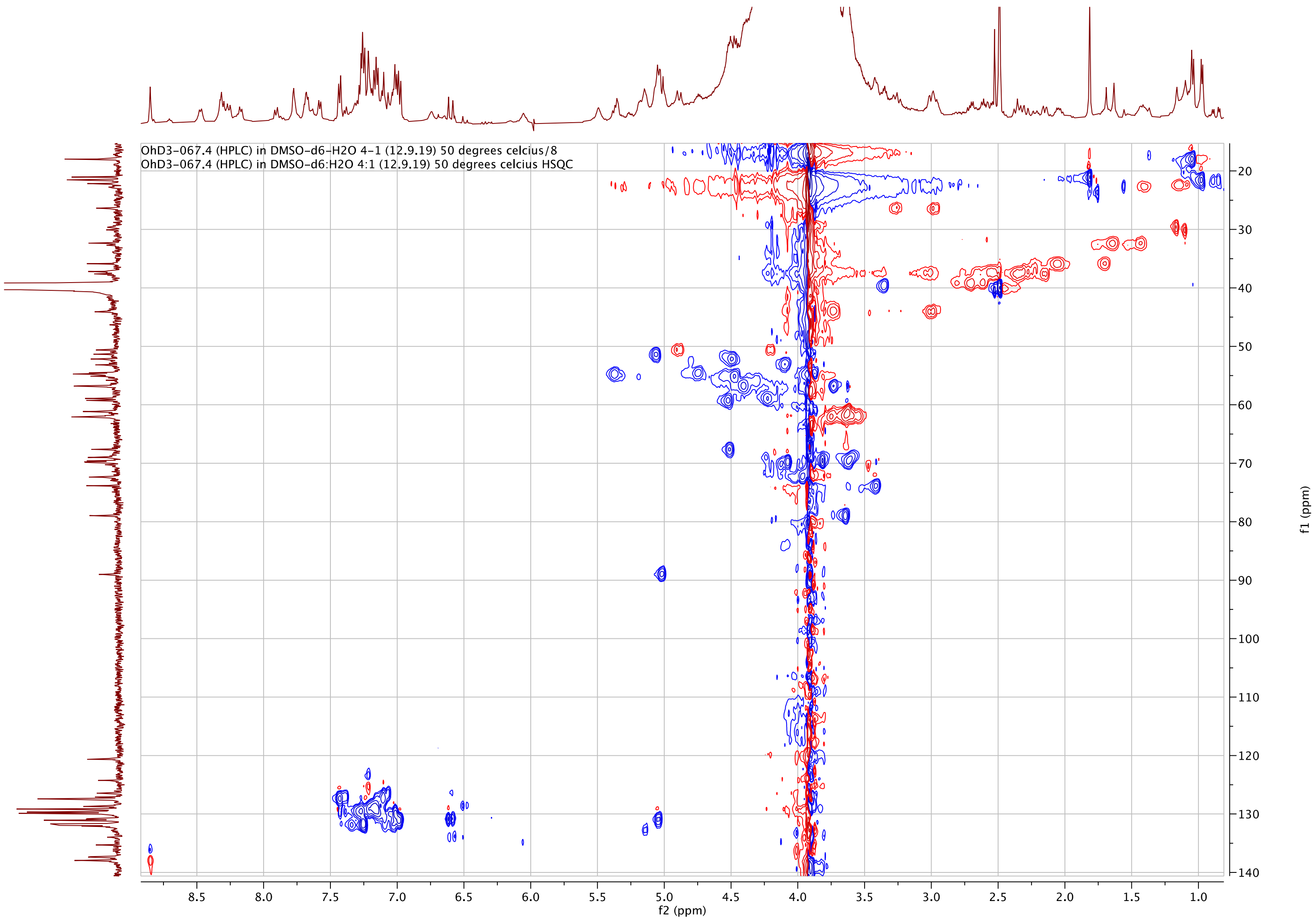

Figure S42. HMBC spectrum of 5-*cis*-Apoa-theopalauamide (**2**) in 4:1 DMSO-d_6:H_2O at 50 ºC

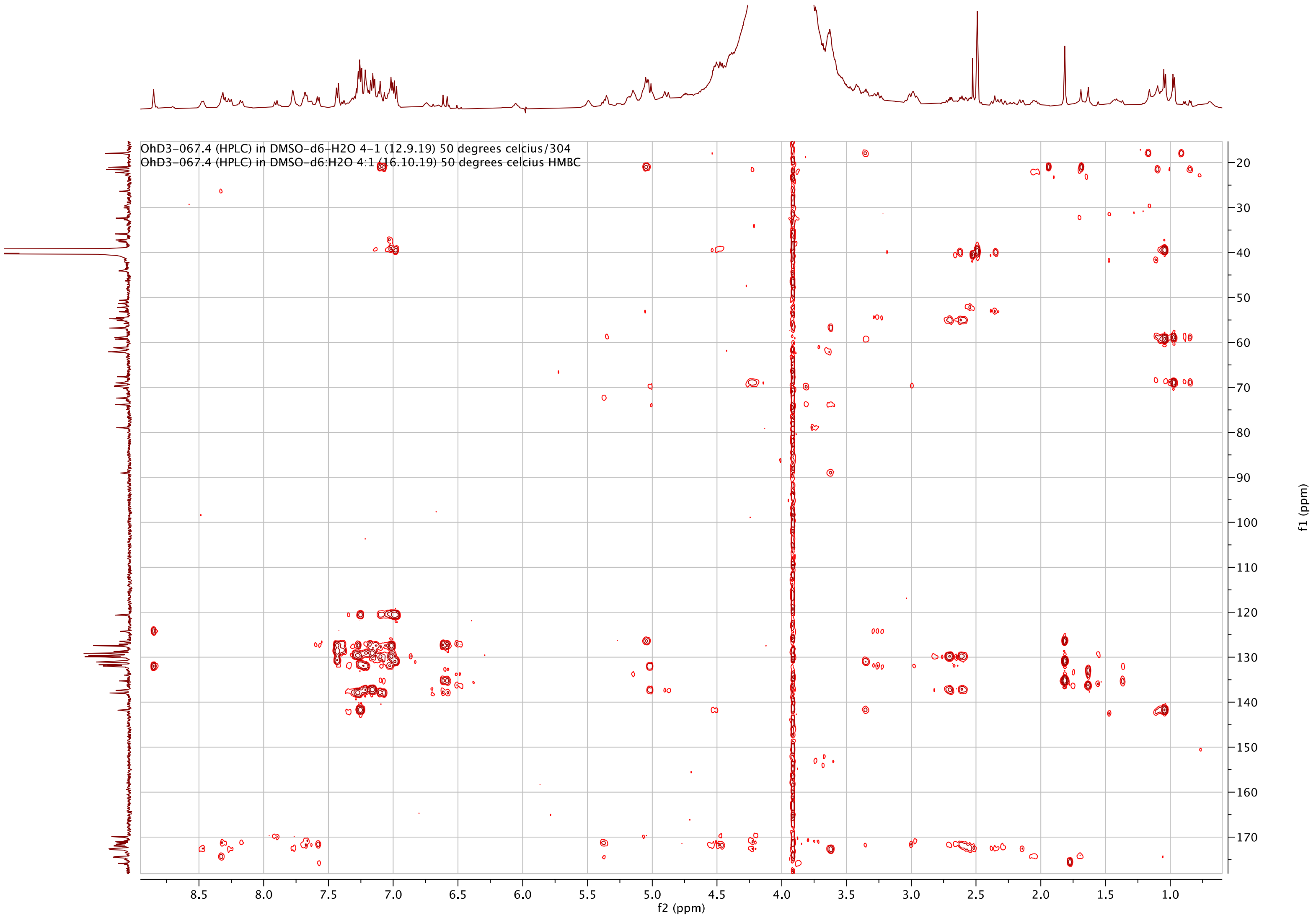

Figure S43. COSY spectrum of 5-*cis*-Apoa-theopalauamide (**2**) in 4:1 DMSO-d_6:H_2O at 50 ºC

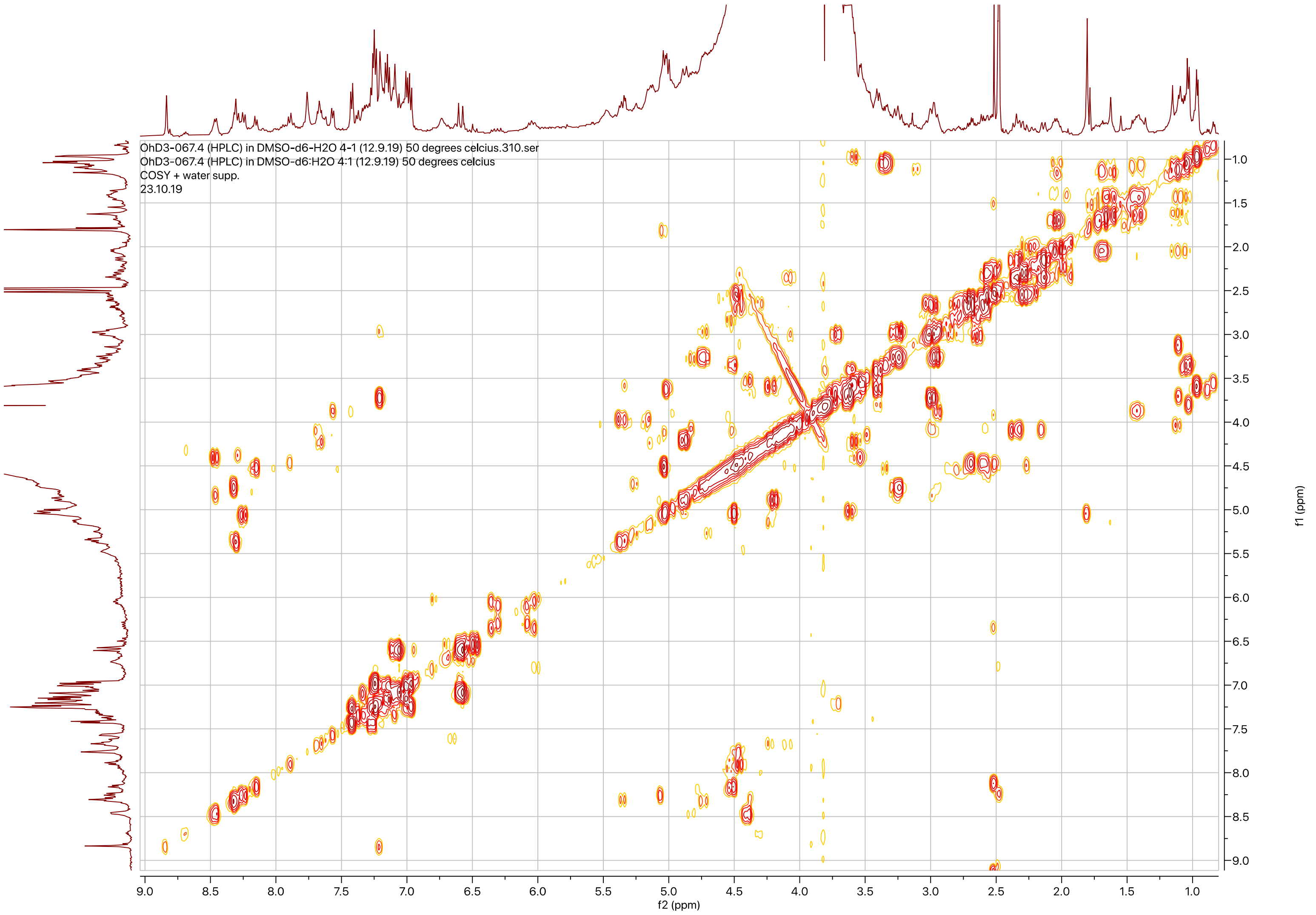

Figure S44. TOCSY spectrum of 5-*cis*-Apoa-theopalauamide (**2**) in 4:1 DMSO-d_6:H_2O at 50 ºC

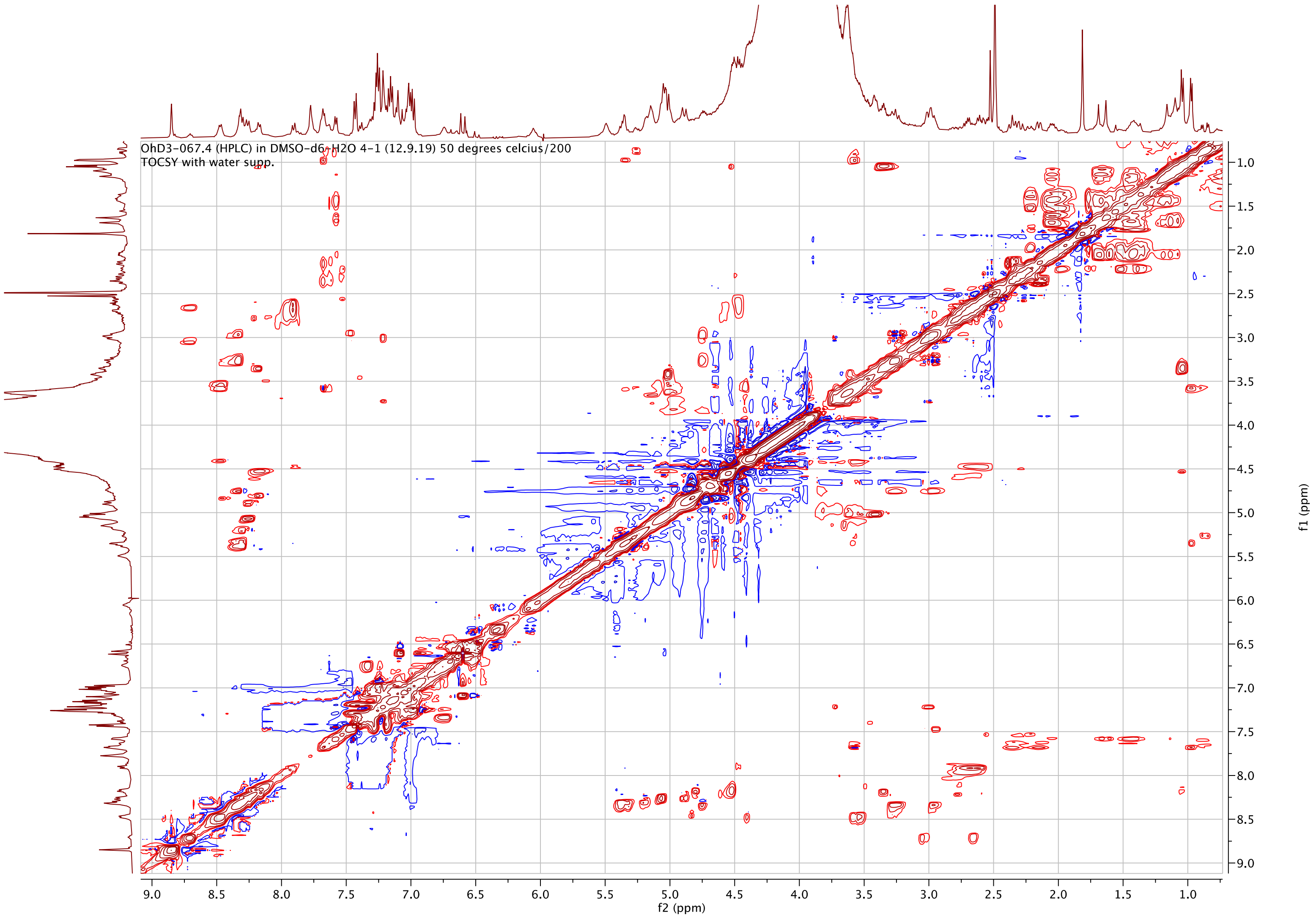

Figure S45. ROESY spectrum of 5-*cis*-Apoa-theopalauamide (**2**) in 4:1 DMSO-d_6:H_2O at 50 °C

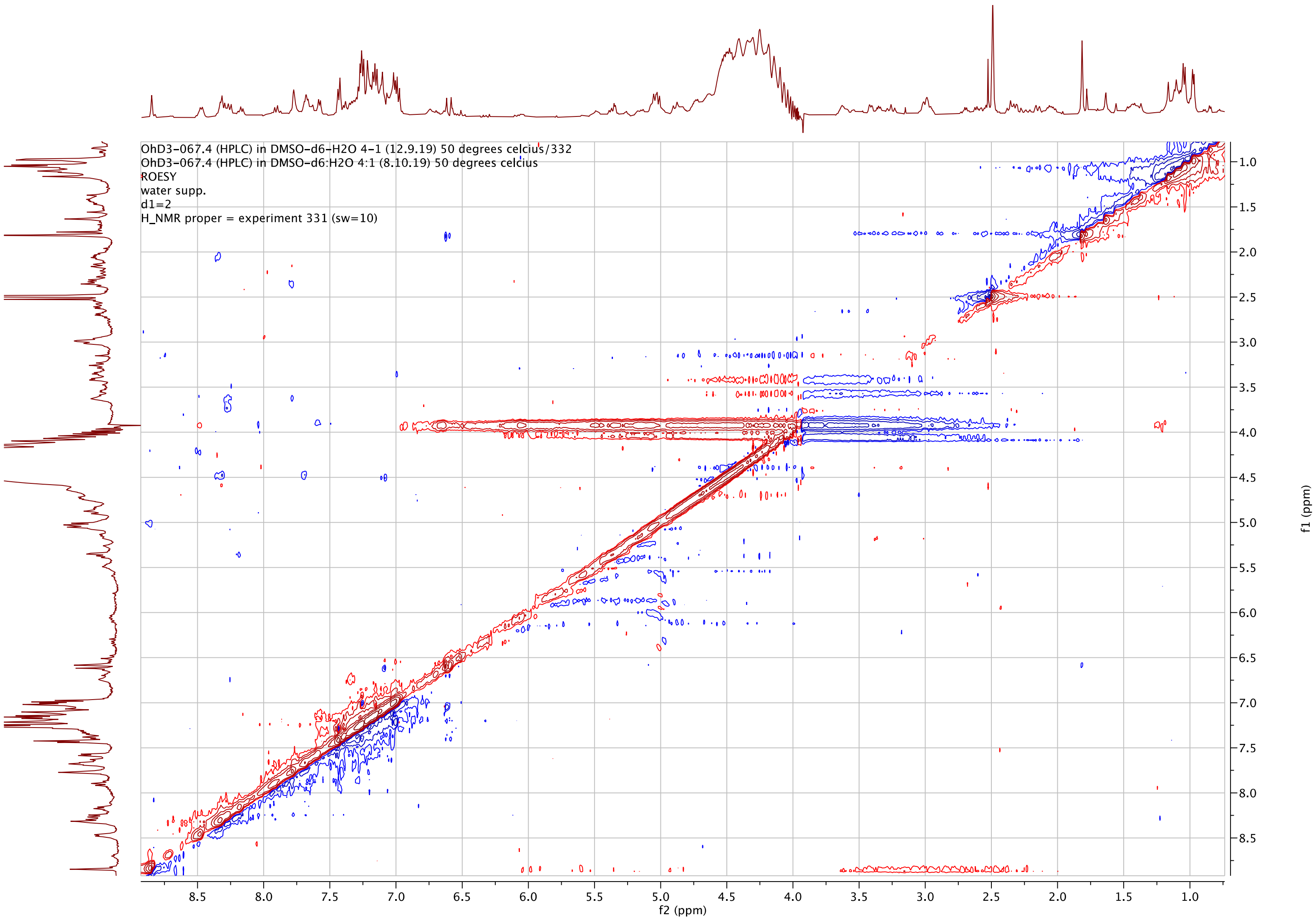

Figure S46. N-H HSQC spectrum of 5-*cis*-Apoa-theopalauamide (**2**) in 4:1 DMSO-d_6:H_2O at 50 ºC

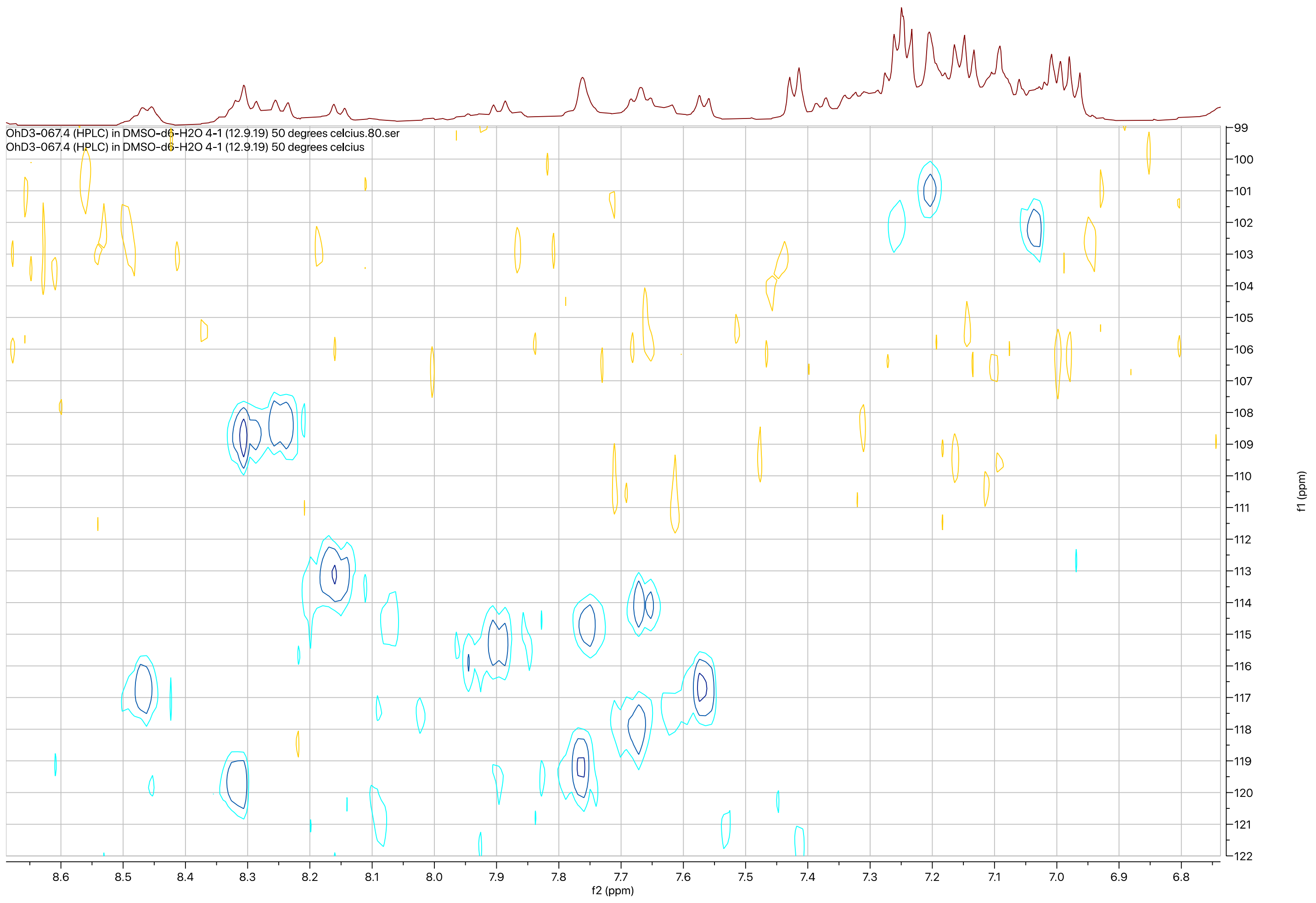

Figure S47. N-H HMBC spectrum of 5-*cis*-Apoa-theopalauamide (**2**) in 4:1 DMSO-d_6:H_2O at 50 ºC

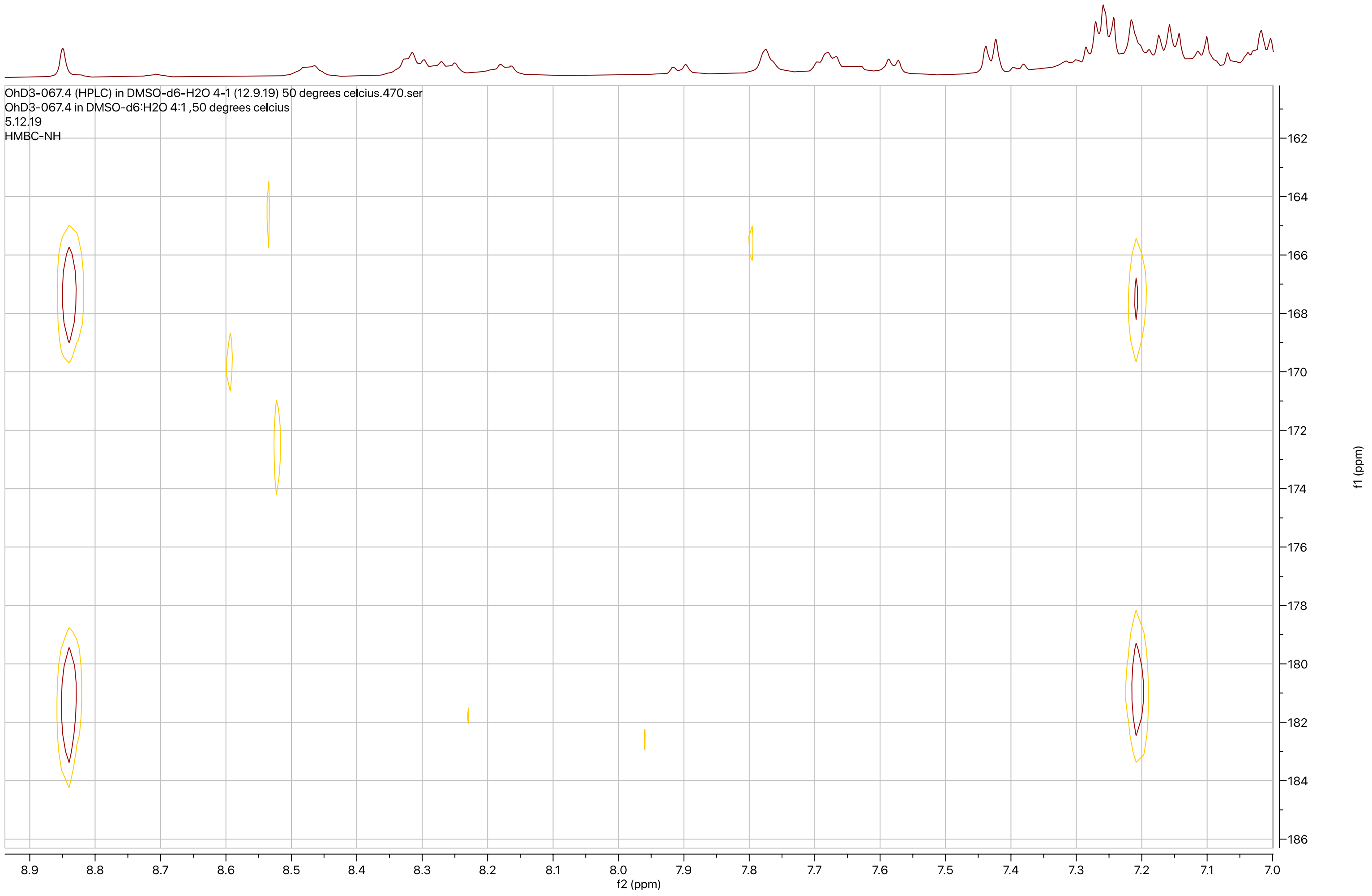

Table S6. NMR data of theopalauamide (**2**) in 4:1 DMSO-d_6:H_2O at 50 $^oC^a$

Position	$\delta_{C/N}$[b]	δ_H[c]	HMBC correlations[d]	COSY correlations	NOE correlations[e]
5-cis-Apoa-1	172.6, C				
2a	37.6, CH_2	2.36, t (12.5)	Apoa-1,3	Apoa-2b,3	Ser[1]-NH
b		2.15, dd (12.5,2.6)	Apoa-1,3	Apoa-2a,3	
3	53.2, CH	4.10, m		Apoa-2a,2b, NH	
3-NH	*118.0*, NH	7.69, d (8.1)	Phe-1	Apoa-3	Phe-2
4	67.7, CH	4.51, m		Apoa-5	
4-OH		5.28, d (5.0)			
5	130.9, CH	5.05, brd (10.1)	Apoa-3,6-Me,7	Apoa-4,6-Me,	Apoa-6-Me
6	135.3, C				
6-Me	21.1, CH_3	1.82, brs	Apoa-5,6,7	Apoa-5	Apoa-5,8
7	126.5, CH	7.09, d (16.0)	Apoa-6,6-Me,8	Apoa-8	
8	130.8, CH	6.60, d (16.0)	Apoa-6,7,9, 10,10’	Apoa-7	Apoa-6-Me
9	138.1, C				
10,10’	127.6, CH x 2	7.43, d x 2 (8.1)	Apoa-8,10’, 10,12	Apoa-11,11’	Apoa-11,11’
11,11’	129.7, CH x 2	7.27, dd (8.1,7.4)	Apoa-9,11’, 11	Apoa-10,10’,12	Apoa-10,10’
12	129.2, CH	7.17, t (7.4)	Apoa-10,10’	Apoa-11,11’	
Ser[1]-1	172.8, C				
2	56.8, CH	3.74, m	Ser[1]-3	Ser[1]-NH,3	sAla-2-NH
2-NH	*114.7*, NH	7.77, m	Apoa-1,	Ser[1]-2	Apoa-2a, Ser[1]-3
3	61.2, CH_2	3.62, m	Ser[1]-1,2	Ser[1]-2	Ser[1]-NH, sAla-NH
3-OH					
sAla-1	169.88, C				
2	51.4, CH	5.06, m	sAla-1	sAla-2-NH, 3a,3b	
2-NH	*108.5*, NH	8.26, d (10.9)	Ser[1]-1,	sAla-2	Ser[1]-2
3a	50.6, CH	4.89, brd (13.4)	sHis-6	sAla-2,3b	
b		4.20, m	sAla-1	sAla-2,3a	sHis-6

Asn-1	171.4, C				
2	52.2, CH	4.49, m	Asn-1	Asn-NH,3a, 3b	Asn-2-NH
2-NH	*119.2*, NH	7.78, s	sAla-1	Asn-2	
3a	37.4, CH_2	2.55, dd (16.0,5.9)	Asn-1,2,4	Asn-2,3b	Asn-4-NH_2b
b		2.30, dd (16.0,2.6)	Asn-1,4	Asn-2,3a	
4	172.5, C				
4-NH_2a	*103.9*, NH_2	7.31, brs			
b		6.73, brs			Asn-3a
Han-1	171.3, C				
2	54.7, CH	5.37, t (9.1)	Han-1,3,4	Han-NH	BrMePhe-2-NH
2-NH	*108.8*, NH	8.30, d (9.2)	Asn-1	Han-2	Asn-3
3	72.4, CH	3.96, m			Asn-NH
3-OH		5.18, d (5.7)		Han-3	
4	174.5, C				
4-NH_2	*102.0*, NH_2	7.04, m			
		7.25, m			
BrMePhe-1	172.0, C				
2	59.2, CH	4.52, m	BrMePhe-1,3,3-Me,4	BrMePhe-NH,3	
2-NH	*113.1*, NH	8.17, d (9.2)	Has-1	BrMePhe-2	Asn-2
3	39.6, CH	3.35, m	BrMePhe-1, 2,3-Me,4,5, 5’	BrMePhe-2,3-Me	BrMePhe-5,5’
3-Me	18.0, CH_3	1.04, d (7.2)	BrMePhe-2,3,4	BrMePhe-3	BrMePhe-5,5’
4	141.8, C				
5,5’	131.1, CH x 2	6.98, d x 2 (8.4)	BrMePhe-3, 5’,5,7	BrMePhe-6,6’	BrMePhe-3, 3-Me
6,6’	131.7, CH x 2	7.25, d x 2 (8.4)	BrMePhe-4,6’,6,7	BrMePhe-5,5’	
7	120.6, C				
iSer-1	171.6, C				
2	69.9, CH	4.08, m	iSer-1	iSer-2-OH, 3a,3b	
2-OH		5.03 m		iSer-2	
3a	44.1, CH_2	3.73, m	BrMePhe-1	iSer-2,3b,NH	

				iSer-2,3a,NH	
b		3.00, brd (14.4)	BrMePhe-1, iSer-1		
3-NH	*101.0*, NH	7.20, m	BrMePhe-1	iSer-3a,3b	
Aad-1	175.9, C				
2	54.7, CH	3.88, m	Aad-1	Aad-2-NH, 3a,3b	Aad-2-NH
2-NH	*116.7*, NH	7.58, d (8.0)	iSer-1, Aad-1	Aad-2	Aad-2
3a	32.4, CH_2	1.65, m		Aad-2,4	
b		1.43, m		Aad-2,4	
4	22.2, CH_2	1.11, m		Aad-3a,3b, 5a,5b	
5a	35.9, CH_2	2.05, dt (13.3,7.5)	Aad-6,4	Aad-5b,4	sHis-NH
b		1.70, m	Aad-6	Aad-5a,4	
6	174.3, C				
sHis-1	171.0, C				
2	54.5, CH	4.74, ddd (12.8,7.3,5.7)		sHis-NH, 3a,3b	Thr-NH
2-NH	*119.6*, NH	8.32, d (5.9)	Aad-6, sHis-3	sHis-2	Aad-5a
3a	26.4, CH_2	3.26, t (13.3)	sHis-1,2,4,8	sHis-2,3b	
b		2.96, m	sHis-1,2,8	sHis-2,3a	
4	132.0, C				
5-N	*181.2*, N				
6	137.4, CH	8.85, s	sHis-5-N,7-N,4,8, Gal-1,	sHis-8	sAla-3b, Gal-1
7-N	*167.4*, N				
8	124.3, CH	7.21, brs	sHis-5-N,7-N,4,6	sHis-6	
Thr-1	172.7, C				
2	58.9, CH	4.23, m	sHis-1, Thr-1,3,4	Thr-NH,3	Ser^{2}-2-NH
2-NH	*114.1*, NH	7.67, d (7.4)	sHis-1	Thr-2	sHis-2, Ser^{2}-3
3	68.9, CH	3.59, m		Thr-4	Ser^{2}-2-NH
3-OH		5.35, d (5.9)	Thr-2		
4	21.5, CH_3	0.97, d (6.4)	Thr-3	Thr-3	
Ser^{2}-1	169.91, C				
2	56.8, CH	4.41, m	Ser^{2}-3	Ser^{2}-NH	

2-NH	*116.6*, NH	8.47, d (8.0)	Thr-1	Ser^2-2	Thr-2,3
3	62.1, CH_2	3.57, m			
		3.64, m			
3-OH					
Phe-1	171.8, C				
2	55.1, CH	4.47, m	Ser^2-1, Phe-1,4	Phe-NH	Apoa-3-NH
2-NH	*115.3*, NH	7.91, d (10.2)	Ser^2-1	Phe-2,3a,3b	
3	39.2, CH_2	2.70, dd (12.7,7.2)	Phe-1,2,4,5	Phe-2,3b	
		2.61, dd (12.7,7.7)	Phe-1,2,4,5	Phe-2,3a	
4	137.3, C				
5,5’	129.9, CH x 2	7.01, d x 2 (7.5)	Phe-5’,5,7	Phe-6,6’	
6,6’	129.1, CH x 2	7.16, t x 2 (7.5)	Phe-4,6’,6	Phe-5,5’,7	
7	127.5, CH	7.10, t (7.5)		Phe-6,6’	
Gal-1	89.0, CH	5.02, d (9.8)	sHis-4,6, Gal-3	Gal-2	sHis-6
2	69.8, CH	3.62, m	Gal-3	Gal-1,2-OH, 3	
2-OH		5.53, m		Gal-2	
3	73.8, CH	3.41, m		Gal-2	
4	69.6, CH	3.81, m	Gal-2,3	Gal-5	
5	79.0, CH	3.64, m	Gal-1,6	Gal-4,6a	
6a	62.1, CH_2	3.75, m	Gal-5	Gal-5,6b	
b		3.54, m		Gal-6a	

[a]^{1}H (500 MHz), ^{13}C (125 MHz) and ^{15}N (50 MHz). [b]Multiplicity and assignment from HSQC experiment. [c]Multiplicity (*J* in Hz). [d]HMBC correlations, optimized for 8 Hz. [e]Selected NOEs from ROESY experiment.

Figure S48. (+)-HRESIMS data of 5-*cis*-Apoa-theopalauamide (**2**)

Elemental Composition Report

Single Mass Analysis

Tolerance = 20.0 PPM / DBE: min = -1.5, max = 400.0
Element prediction: Off
Number of isotope peaks used for i-FIT = 5

Monoisotopic Mass, Even Electron Ions
82 formula(e) evaluated with 25 results within limits (up to 3 closest results for each mass)
Elements Used:
C: 70-80 H: 90-100 N: 10-20 O: 25-30 79Br: 1-1 Na: 0-1

ohD3-067/4 Carmeli1320 16 (0.718) Cm (14:22)
Ohad Hasin
1: TOF MS ES+

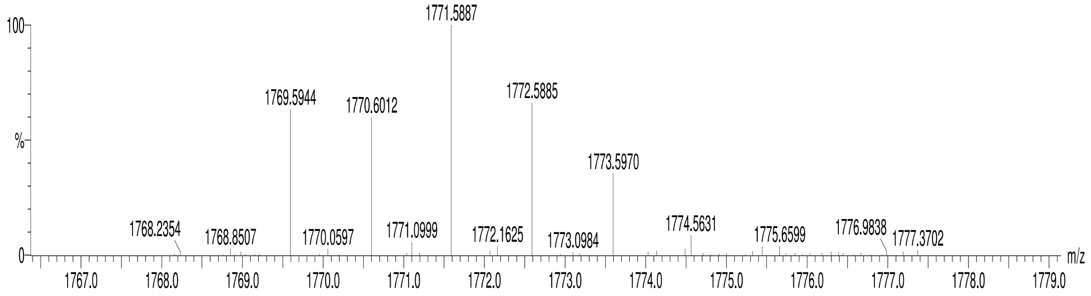

Minimum:				-1.5			
Maximum:		5.0	20.0	400.0			

Mass	Calc. Mass	mDa	PPM	DBE	i-FIT	i-FIT (Norm)	Formula
1769.5944	1769.5947	-0.3	-0.2	34.5	91.1	1.1	C76 H99 N16 O27 79Br Na
	1769.5930	1.4	0.8	33.5	91.1	1.1	C73 H98 N18 O29 79Br
	1769.5971	-2.7	-1.5	37.5	91.0	1.1	C78 H98 N16 O27 79Br

Figure S49. ^{1}H NMR spectrum (500 MHz) of theonellamide K (**3**) in 4:1 DMSO-d_6:H_2O at 50 ^{o}C

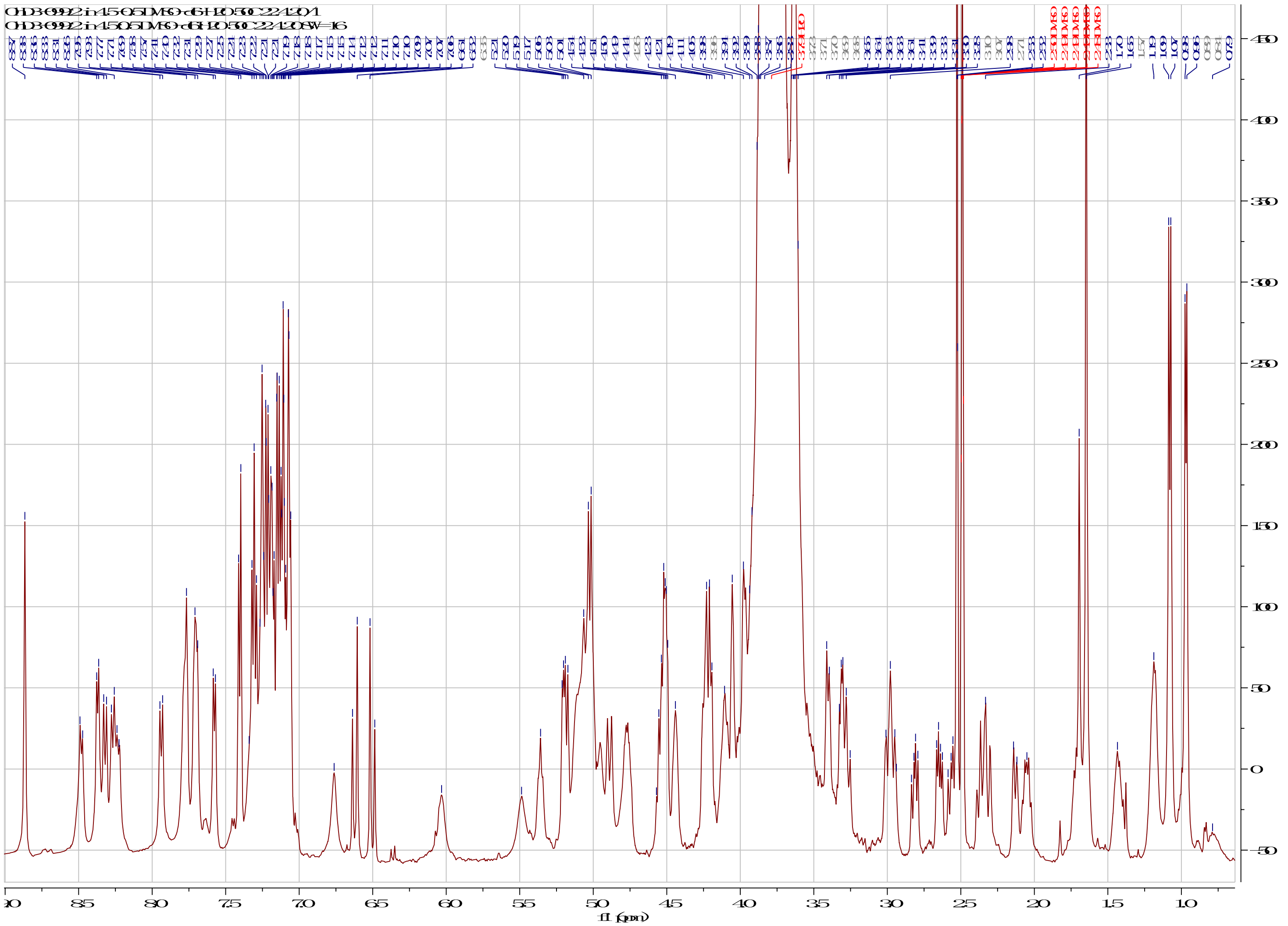

Figure S50. ^{13}C NMR spectrum (125 MHz) of theonellamide K (**3**) in 4:1 DMSO-d_6:H_2O at 50 ^{o}C

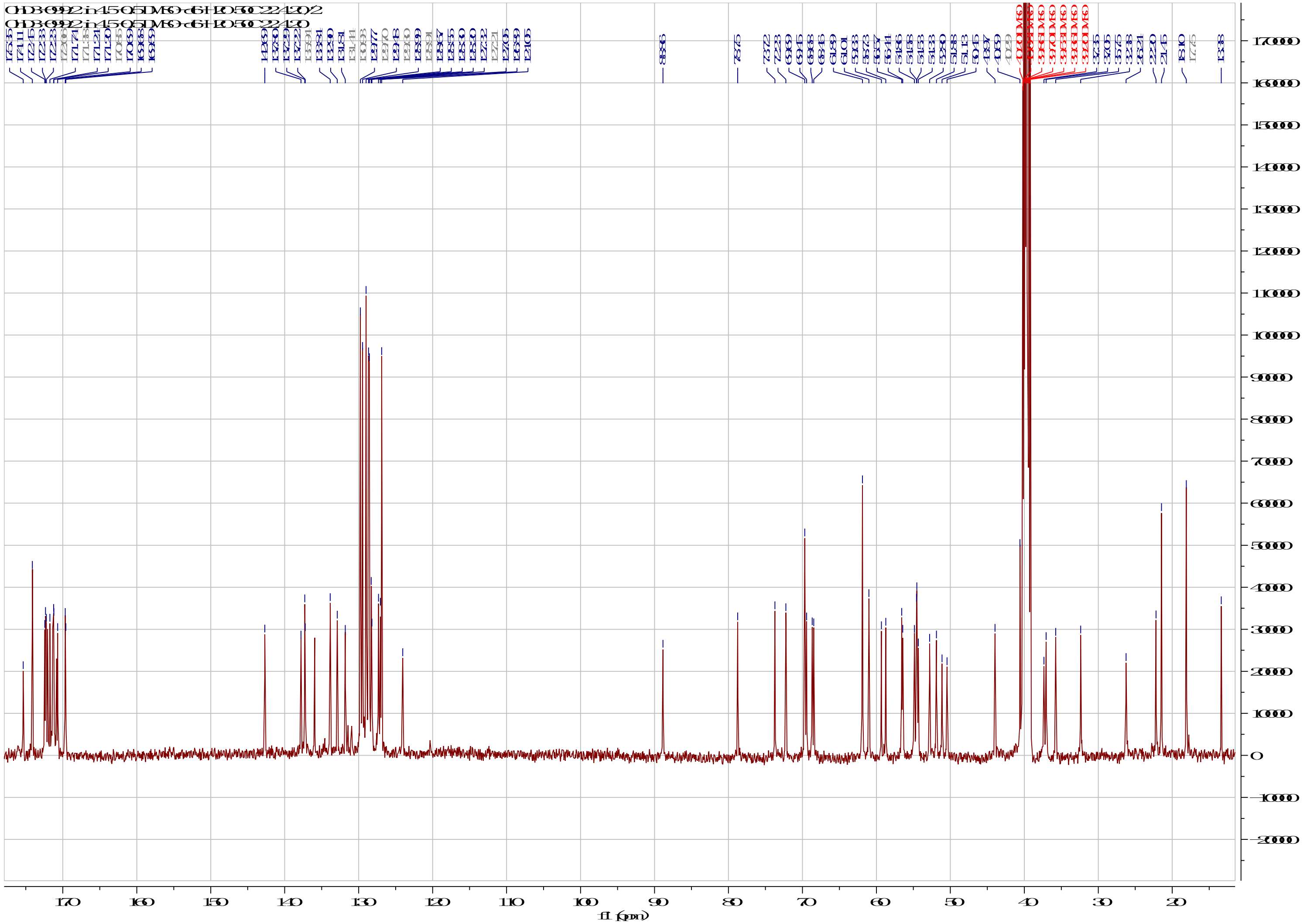

Figure S51. HSQC spectrum of theonellamide K (**3**) in 4:1 DMSO-d_6:H_2O at 50 ºC

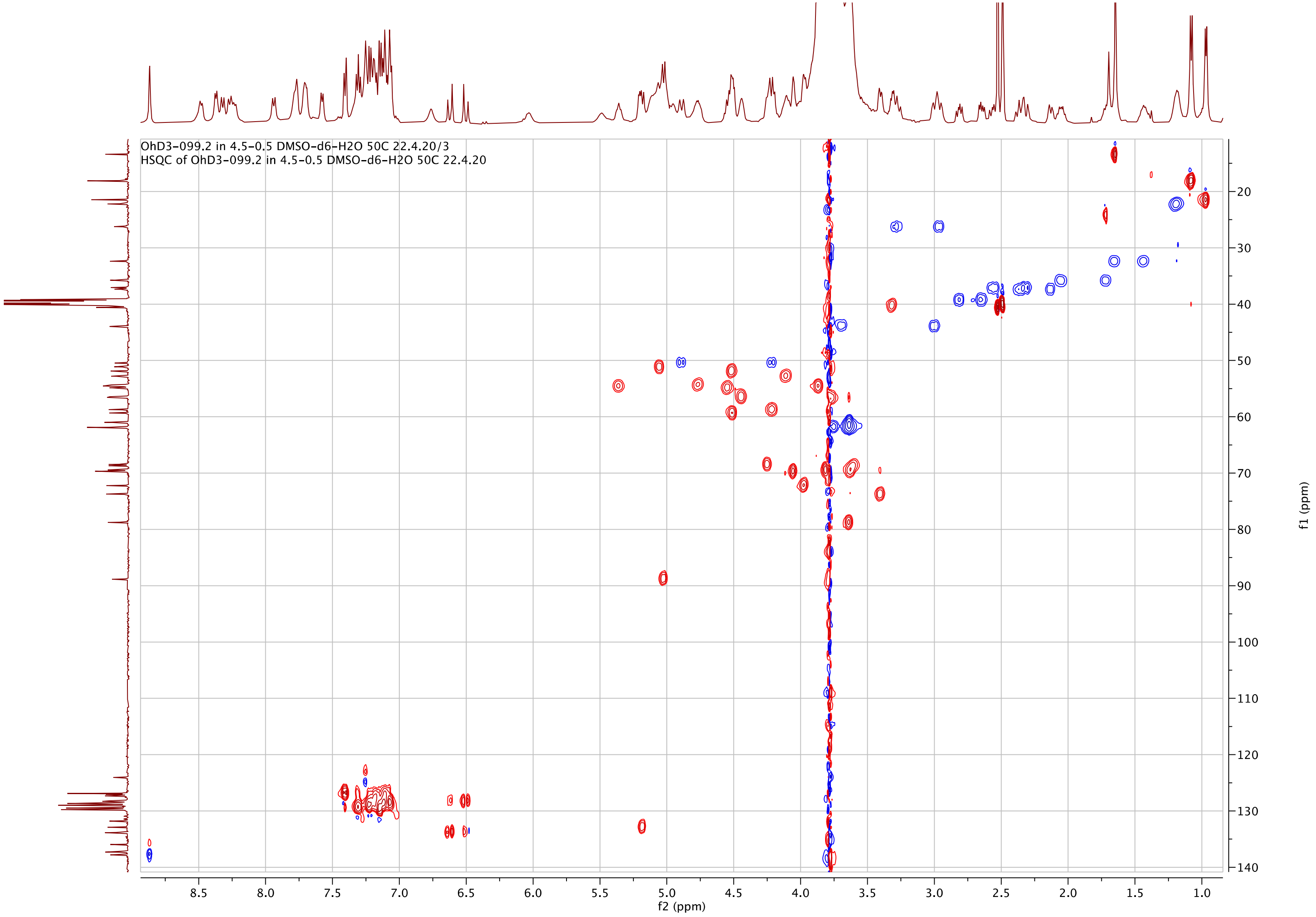

Figure S52. HMBC spectrum of theonellamide K (**3**) in 4:1 DMSO-d_6:H_2O at 50 ºC

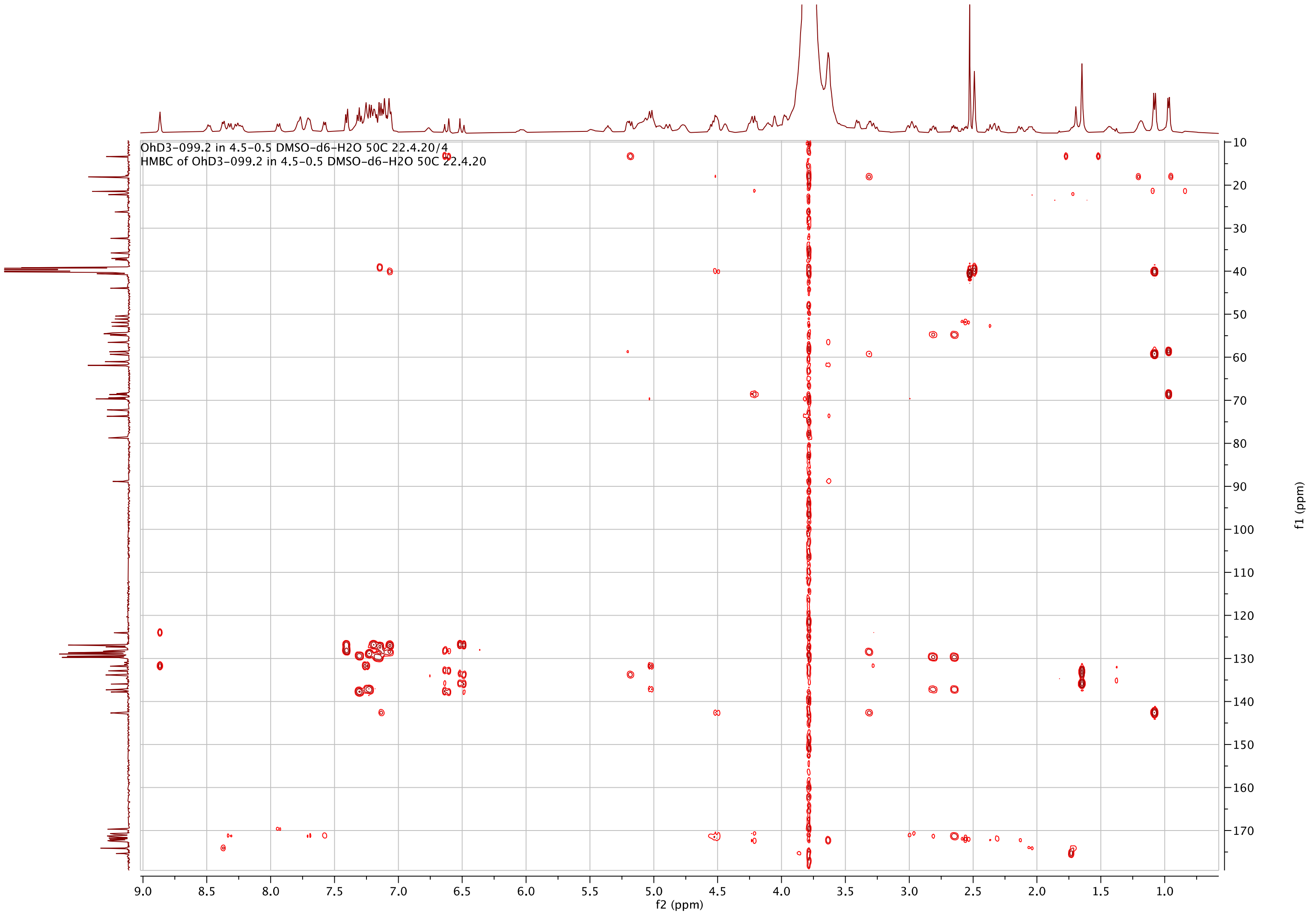

Figure S53. COSY spectrum of theonellamide K (**3**) in 4:1 DMSO-d_6:H_2O at 50 ^oC

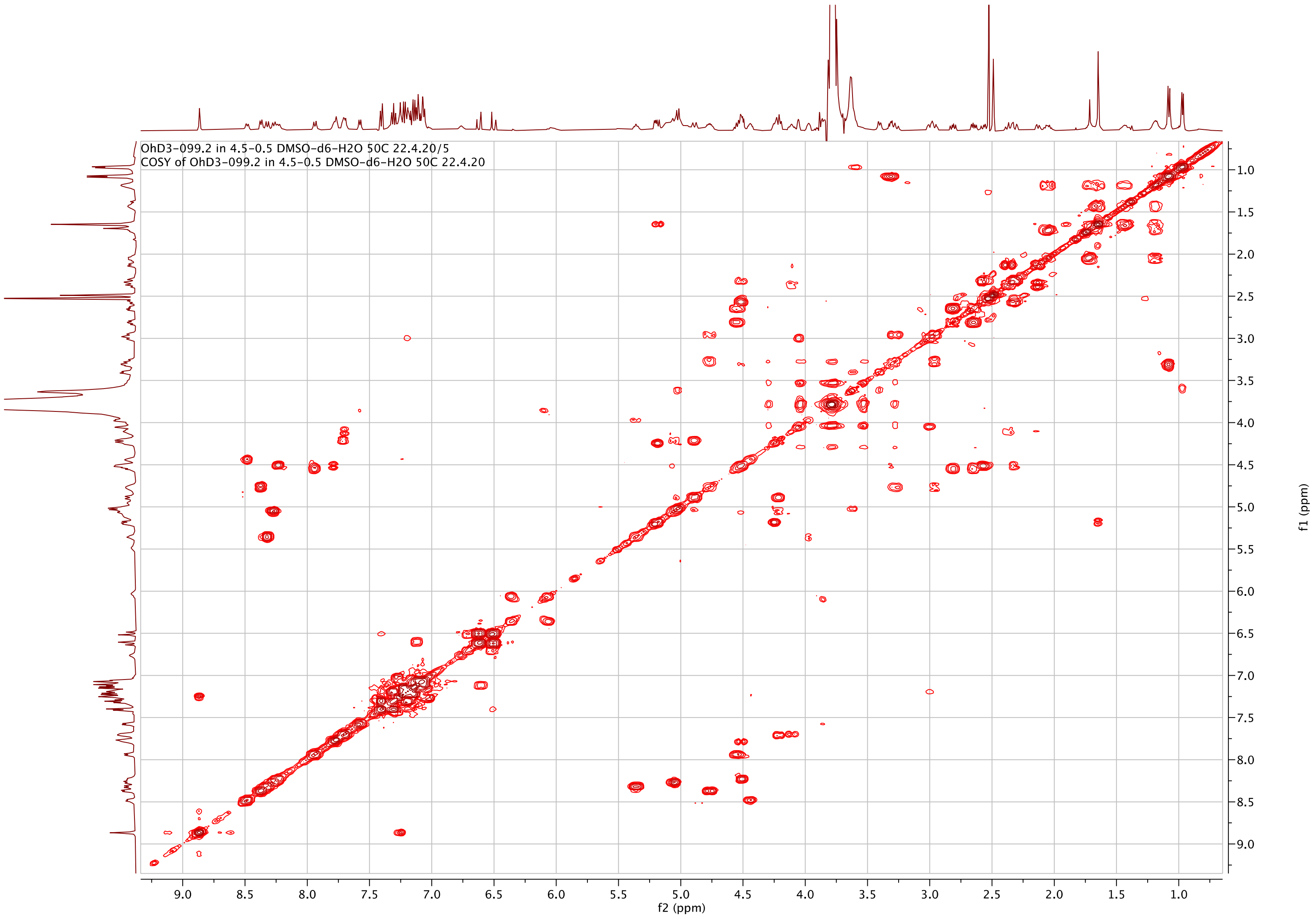

Figure S54. TOCSY spectrum of theonellamide K (**3**) in 4:1 DMSO-d_6:H_2O at 50 ºC

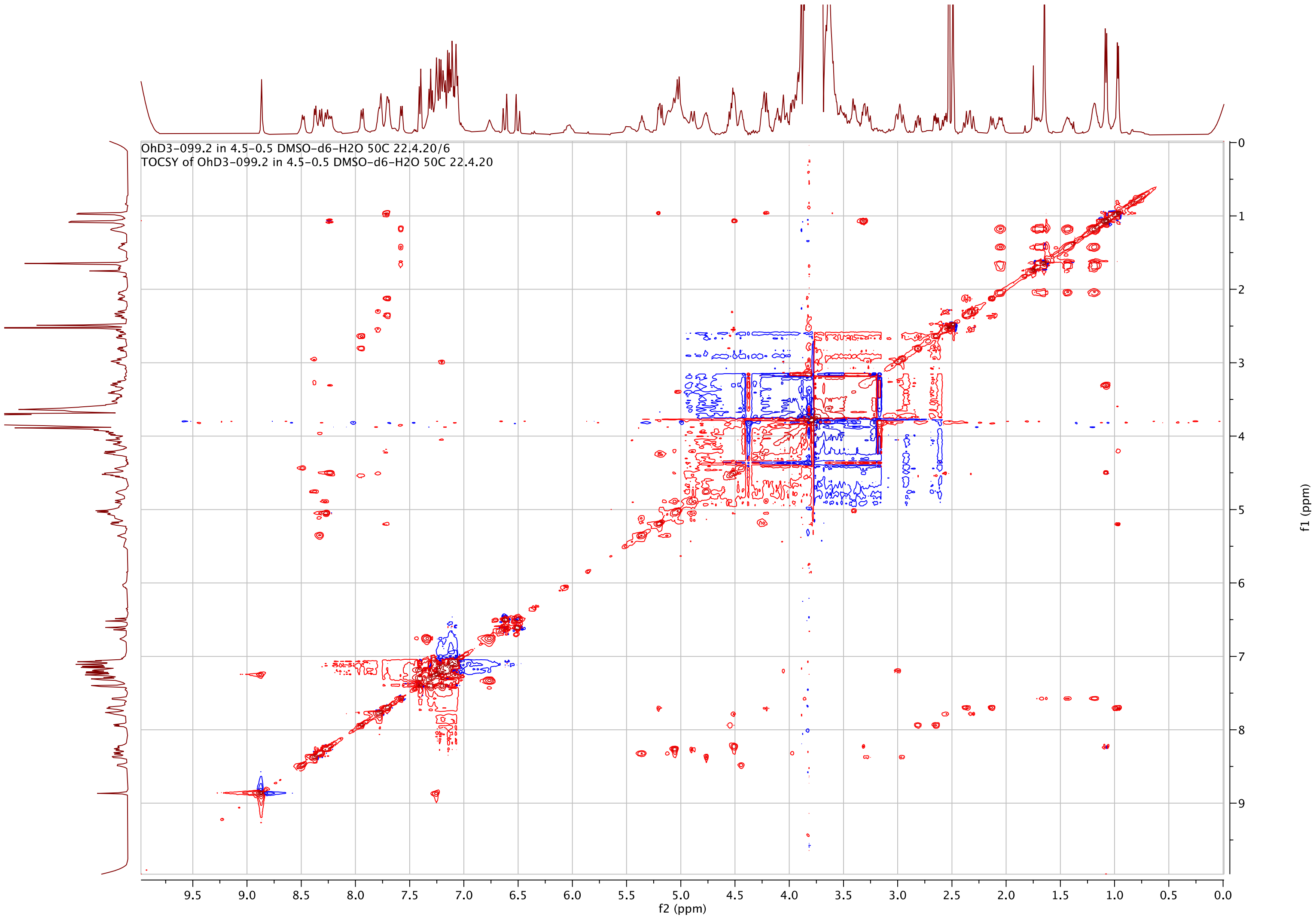

Figure S55. ROESY spectrum of theonellamide K (**3**) in 4:1 DMSO-d_6:H_2O at 50 ºC

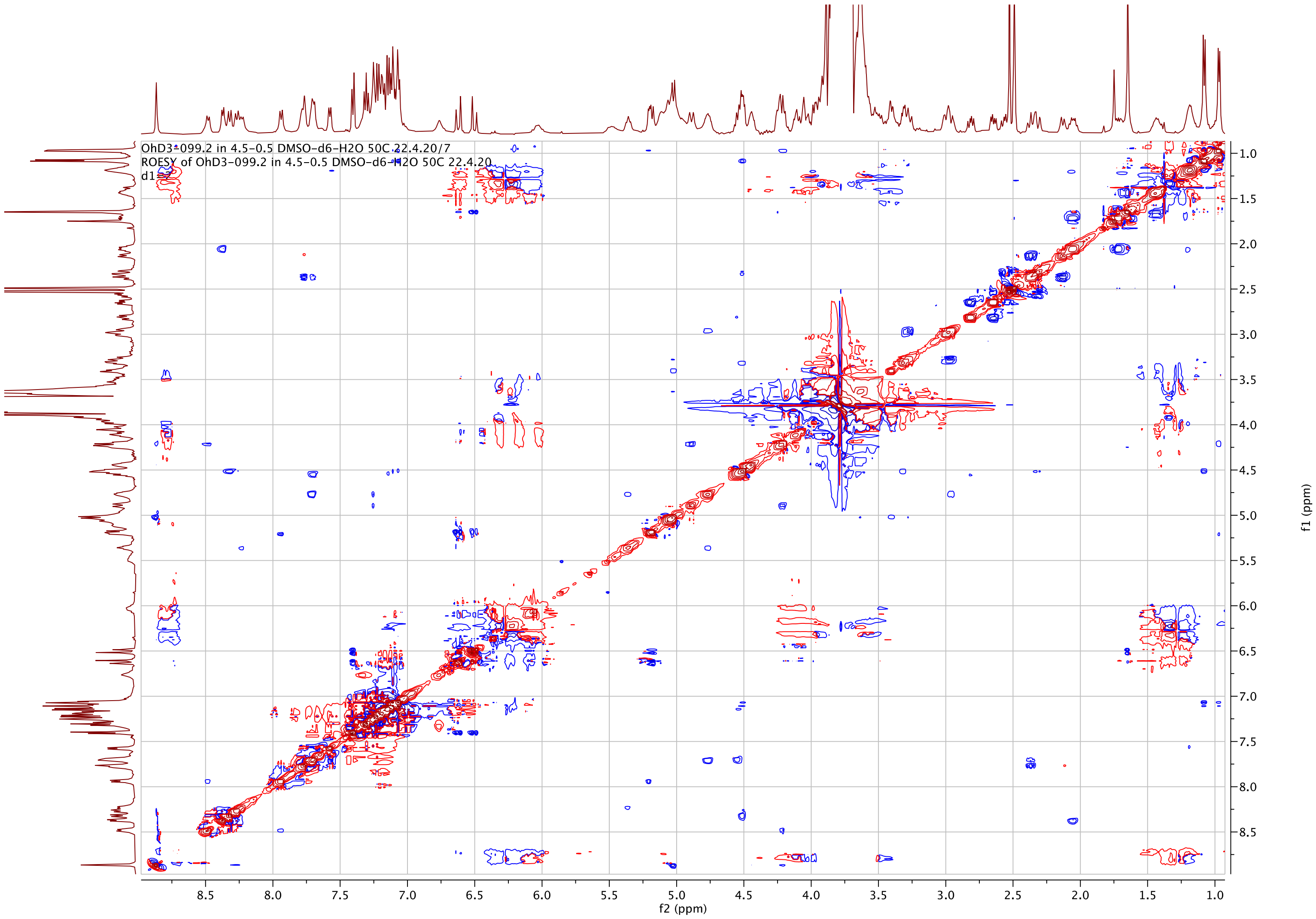

Figure S56. N-H HSQC spectrum theonellamide K (**3**) in 4:1 DMSO-d_6:H_2O at 50 ^oC

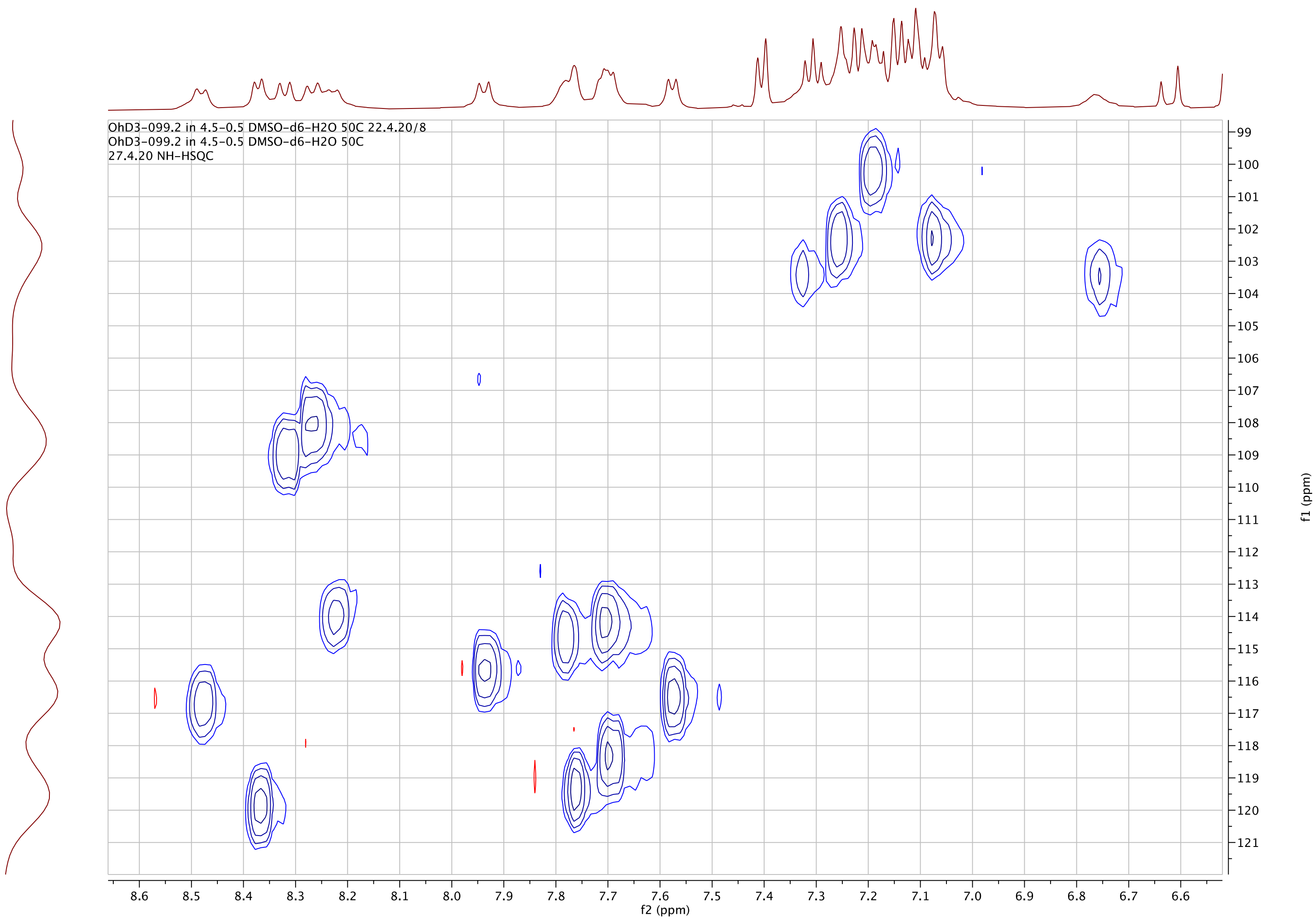

Figure S57. N-H HMBC spectrum of theonellamide K (**3**) in 4:1 DMSO-d_6:H_2O at 50 ºC

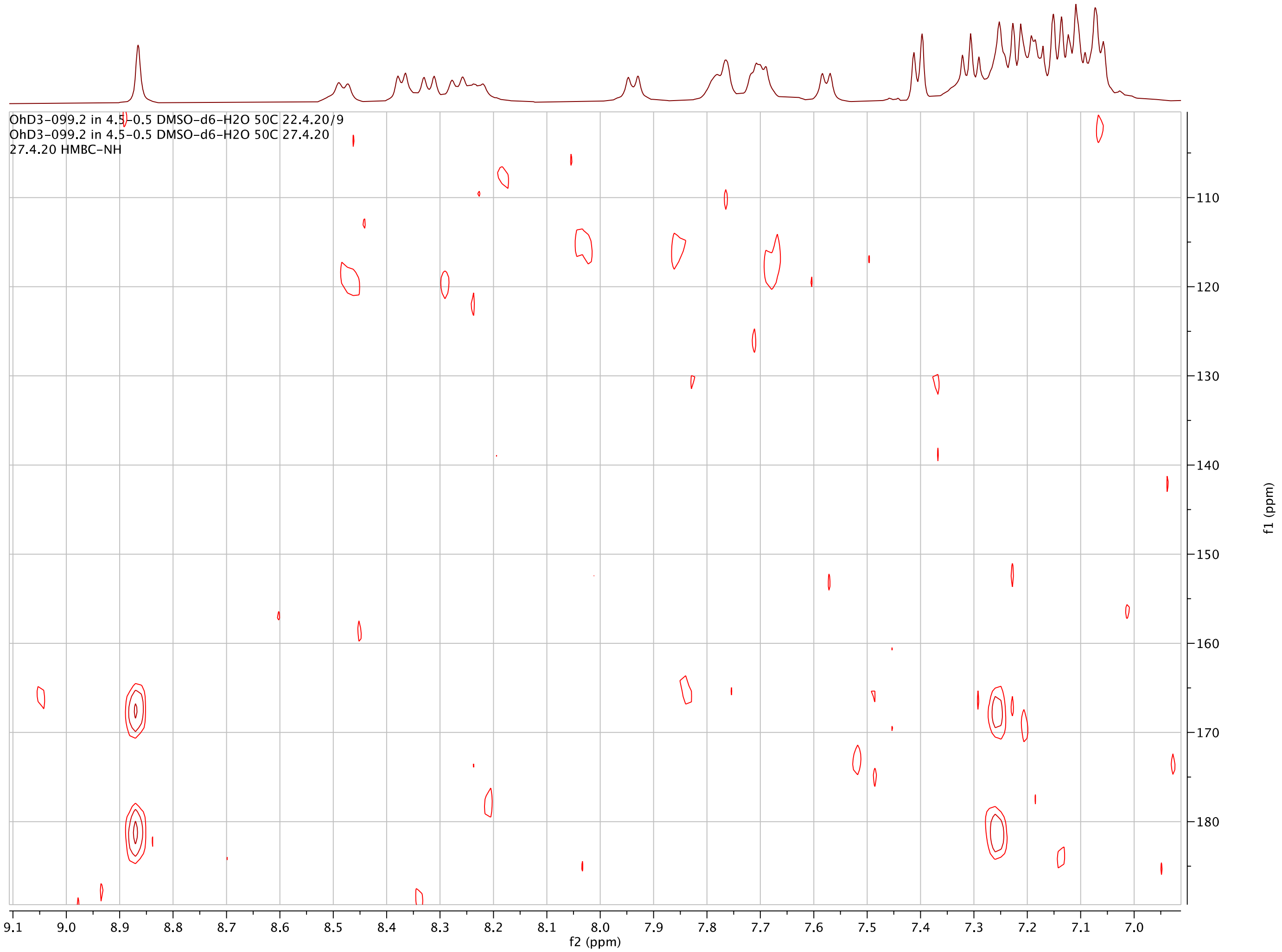

Table S7. NMR data of theonellamide K (**3**) in 4:1 DMSO-d_6:H_2O at 50 ^oC[a]

Position	$\delta_{C/N}$[b]	δ_H[c]	HMBC correlations[d]	COSY correlations	TOCSY correlations	NOE correlations[e]
Apoa-1	172.2, C					
2a	37.4, CH_2	2.37, t (12.5)	Apoa-1,3	Apoa-2b,3	Apoa-3-NH,	Ser[1]-2-NH, Apoa-2b, 2-NH
b		2.13, dd (12.5,2.8)	Apoa-1,3	Apoa-2a,3	Apoa-3-NH	Apoa-2a,3 ,4, Ser[1]-2-NH(neg)
3	52.8, CH	4.11, m	Phe-1	Apoa-2a, 2b,3-NH	Apoa-3-NH	Apoa-2b, 3-NH,5
3-NH	*118.4, NH*	7.70, d (8.6)	Apoa-3	Apoa-3	Apoa-2a,2b,3	Apoa-3,5, Phe-2
4	68.5, CH	4.25, m		Apoa-5	Apoa-5	Apoa-2b,6-Me
5	132.9, CH	5.18, d (9.0)	Apoa-3,4, 6-Me,7	Apoa-4,6-Me	Apoa-4	Apoa-3,3-NH,7
6	135.9, C					
6-Me	13.4 CH_3	1.65, s	Apoa-4,5, 6,	Apoa-5	Apoa-7,8	Apoa-4,7,8
7	133.8, CH	6.62, d (16.2)	Apoa-5,6,6-Me,8,9	Apoa-8	Apoa-6-Me,8	Apoa-5,6-Me,10,10’
8	128.2, CH	6.50, d (16.2)	Apoa-6,7,9,10,10’	Apoa-7	Apoa-6-Me,7	Apoa-5,6-Me,10,10’
9	137.8, C					
10,10’	126.9, CH x 2	7.41, d (7.7)	Apoa-8,10’, 10,12	Apoa-11,11’	Apoa-11,11’	Apoa-7,8
11,11’	129.5, CH x 2	7.31, t (7.7)	Apoa-9,11’, 11,	Apoa-10,10’,12	Apoa-10,10’,12	
12	128.3, CH	7.19, t (7.7)	Apoa-10, 10’	Apoa-11,11’	Apoa-11,11’	
Ser[1]-1	172.3, C					
2	56.6, CH	3.78, m	Ser[1]-1			
2-NH	*119.4, NH*	7.77, s	Apoa-1, Ser[1]-2,3			Apoa-2a, 2b (neg)
3	61.0, CH_2	3.64, m	Ser[1]-1			sAla-2-NH
sAla-1	169.6, C					
2	51.1, CH	5.06, m	sAla-1,3,	sAla-2-NH,3a,3b	sAla-2-NH	sAla-2-NH,3b, Asn-2-NH, sHis-3a
2-NH	*108.1, NH*	8.27, d (9.9)	Ser[1]-1, sAla-2	sAla-2	sAla-2,3a,3b	sAla-2, Ser[1]-3
3a	50.5, CH_2	4.89, d (13.6)	sAla-2,	sAla-2,3b	sAla-2-NH	sHis-8, sAla-3b
b		4.22, dd (13.6,10.0)	sHis-6, sAla-1,2	sAla-2,3a	sAla-2-NH	sAla-2,3a, sHis-6

Asn-1	171.2, C					
2	51.9, CH	4.52, m	Asn-3	Asn-2,3a,3b	Asn-2-NH	Has-2-NH Asn-2-NH,3b
2-NH	*114.7, NH*	7.78, brs	sAla-1	Asn-2	Asn-2,3a,3b	sAla-2 Asn-2,3a
3a	37.1, CH_2	2.56, dd (16.2,9.9)	Asn-1,2,4	Asn-2,3b	Asn-2-NH	Asn-2-NH,3b
b		2.32, dd (16.2,2.4)	Asn-1,2,4	Asn-2,3a	Asn-2-NH	Asn-2,3a, Has-2-NH
4	172.1, C					
4-NH_2	*103.5, NH_2*	7.32, brs		4-NHb	Asn-4-NHb	Asn-4-NHb (ex)
		6.76, brs		4-NHa	Asn-4-NHa	Asn-4-NHa (ex)
Han-1	170.9, C					
2	54.5, CH	5.36, dd (8.9,7.5)	Han-1,3,4	Han-2-NH,3	Han-2-NH	MePhe-2-NH, sHis-2, Han-3
2-NH	*108.9, NH*	8.32, d (9.6)	Asn-1	Han-2	Han-2,3,3-OH	Asn-2,3b, Han-3
3	72.2, CH	3.97, brd (7.5)	Han-1,2,4	Han-2,3-OH		Han-2,2-NH,4-NHa
3-OH		5.12, brs		Han-3		
4	174.1, C					
4-NH_2	*102.3, NH_2*	7.25, s	Han-3,4		Han-4-NHb	Han-3
		7.07, s			Han-4-NHa	
MePhe-1	171.7, C					
2	59.3, CH	4.51, dd (8.2,4.8)	MePhe-1,3,3-Me,4	MePhe-2-NH,3	MePhe-2-NH,	MePhe-2-NH,3,3-Me,5,5’
2-NH	*114.0, NH*	8.23, d (8.5)	Han-1	MePhe-2	MePhe-2-NH,3,3-Me	Han-2, MePhe-2,3
3	40.1, CH	3.31, dq (4.8,7.1)	MePhe-1,2, 3-Me,4, 5,5’	MePhe-2,3-Me	MePhe-2-NH,	MePhe-2,2-NH,3-Me,5,5’
3-Me	18.1, CH_3	1.08, d (7.1)	MePhe-2,3,4	MePhe-3	MePhe-2-NH,	MePhe-2, 3,5,5’
4	142.4, C					
5,5’	128.6, CH x 2	7.07, d (7.2)	MePhe-3,4, 5’,5,7	MePhe-6,6’		MePhe-2,3,3-Me
6,6’	128.7, CH x 2	7.12, m	MePhe-4,6’,6	MePhe-5,5’		
7	127.1, CH	7.11, t (6.8)	MePhe-5,5’			
iSer-1	171.2, C					
2	69.7, CH	4.05, brs	iSer-1	iSer-3b	iSer-3-NH	iSer-3b, Ada-2-NH

3a	44.0, CH_2	3.69, m	iSer-2	iSer-3b,	iSer-3-NH	iSer-3b
b		3.00, dd (11.8,3.5)	iSer-2	iSer-2,3a		iSer-2,3a
3-NH	*100.2, NH*	7.19, m	MePhe-1	iSer-3a,3b	iSer-2,3a	
Ada-1	175.4, C					
2	54.6, CH	3.87, m	Ada-1,4	Ada-2-NH,3b		
2-NH	*116.5, NH*	7.58, d (7.1)	iSer-1, Ada-1	Ada-2	Ada-2,3a, 3b,4	Ada-4, iSer-2
3a	32.4, CH_2	1.66, m	Ada-1,2,4,5	Ada-3b,4 Ada-	Ada-2-NH	
b		1.43, m	Ada-2,4,5	2,3a, 4		
4	22.2, CH_2	1.19, m 2H	Ada-3,5,6	Ada-3a, 3b,5a,5b	Ada-2-NH	Ada-2-NH
5a	35.8, CH_2	2.05, dt (13.5,8.5)	Ada-3,4,6	Ada-3b,4		sHis-2-NH, Ada-4,5b
b		1.72, m	Ada-3,4,6	Ada-3a,4		Ada-5a
6	174.1, C					
sHis-1	170.7, C					
2	54.3, CH	4.77, m	Ada-6, sHis-1,3,	sHis-2-NH,3a,3b	sHis-2-NH	sHis-2-NH,3b,8, sAla-2 Han-2, Thr-2-NH
2-NH	*119.9, NH*	8.37, d (6.6)	Ada-6, sHis-2,3,	sHis-2	sHis-2,3a, 3b	Ada-5a sHis-2,3a, 3b
3a	26.2, CH_2	3.28, t (13.3)	sHis-1,2,4, 8	sHis-2,3b	sHis-2-NH	sAla-2, sHis-3b,2-NH, Gal-1
b		2.96, dd (13.3,4.0)	sHis-1,2,4,	sHis-2,3a	sHis-2-NH	sHis-2,2-NH,3a,8
4	131.8, C					
5	*181.4, N*					
6	137.2, CH	8.87, s	sAla-3, sHis-4,5,7, 8, Gal-1	sHis-8	sHis-8	Gal-1, sAla-3b
7	*167.8, N*					
8	124.1, CH	7.25, s	sHis-4,5,6	sHis-6	sHis-6	sHis-2,3b sAla-3a
Thr-1	172.5, C					
2	58.7, CH	4.22, m	Thr-1,3,3-OH,4 sHis-1	Thr-2-NH,3	Thr-2-NH	Ser^2-2-NH Thr-2-NH,4
2-NH	*114.1, NH*	7.71, d (8.4)	sHis-1	Thr-2	Thr-2,3-OH	sHis-2 Thr-2,3
3	68.7, CH	3.61, m	Thr-2	Thr-2,4		Thr-2-NH
3-OH		5.20, d (4.8)	Thr-2			Phe-2-NH, Thr-4
4	21.5, CH_3	0.97, d (6.1)	Thr-2	Thr-3		Thr-2,3-OH
Ser^2-1	169.7, C					

2	56.4, CH	4.44, dt (8.9,3.3)	Thr-1, Ser^2-1,3	Ser^2-2-NH,3	Ser^2-2-NH	Phe-2-NH Ser^2-2-NH
2-NH	*116.7, NH*	8.48, d (8.1)	Thr-1	Ser^2-2	Ser^2-2,3	Phe-2-NH Thr-2-NH Ser^2-2,3
3	61.9, CH_2	3.64, m	Ser^2-1,2	Ser^2-2	Ser^2-2-NH	Ser^2-2-NH
Phe-1	171.4, C					
2	54.9, CH	4.55, ddd (8.9,8.3,6.6)	Ser^2-1, Phe-1,3,4	Phe-2-NH, 3a,3b	Phe-2-NH	Phe-2-NH,3a,3b, Apoa-3-NH
2-NH	*115.7*	7.94, d (8.9)	Ser^2-1	Phe-2	Phe-2,3a, 3b	Ser^2-2,2-NH, Thr-3-OH, Phe-2,3a, 3b
3a	39.2, CH_2	2.81, dd (13.5,8.3)	Phe-1,2,4,5, 5’	Phe-2,3b	Phe-2-NH	Phe-2,2-NH,3b
b		2.65, dd (13.5,6.6)	Phe-1,2,4,5, 5’	Phe-2,3a	Phe-2-NH	Phe-2,2-NH,3a, 5,5’
4	137.3, C					
5,5’	129.8, CH x 2	7.14, m	Phe-3,7	Phe-6,6’		Phe-3b
6,6’	129.0, CH x 2	7.22, d (7.4)	Phe-4,6’,6	Phe-5,5’,7		
7	127.3, CH	7.18, m	Phe-5,5’	Phe-6,6’		
Gal-1	88.9, CH	5.02, d (9.3)	sHis-4,6 Gal-2,3,5	Gal-2		sHis-3a,6 Gal-2,3
2	69.7, CH	3.63, m	Gal-1,	Gal-1,3		Gal-1
3	73.7, CH	3.40, brd (8.0)	Gal-2,	Gal-2		Gal-1
4	69.5, CH	3.81, m	Gal-3			
5	78.8, CH	3.64, m	Gal-3			
6	61.9, CH_2	3.75, m 3.64, m	Gal-4,5, Gal-4			

[a] ^{1}H (500 MHz), ^{13}C (125 MHz) and ^{15}N (50 MHz). [b] Multiplicity and assignment from HSQC experiment. [c] Multiplicity (*J* in Hz). [d] HMBC correlations, optimized for 8 Hz. [e] Selected NOEs from ROESY experiment.

Figure S58. (+)-HRESIMS data of theonellamide K (**3**)

Elemental Composition Report

Single Mass Analysis

Tolerance = 20.0 PPM / DBE: min = -1.5, max = 400.0
Element prediction: Off
Number of isotope peaks used for i-FIT = 5

Monoisotopic Mass, Even Electron Ions
211 formula(e) evaluated with 22 results within limits (up to 3 closest results for each mass)
Elements Used:
C: 70-80 H: 90-120 N: 0-20 O: 20-30

OhD3-099/2 Carmeli 1335 81 (3.577) Cm (76:81)
Ohad Hasin
1: TOF MS ES+

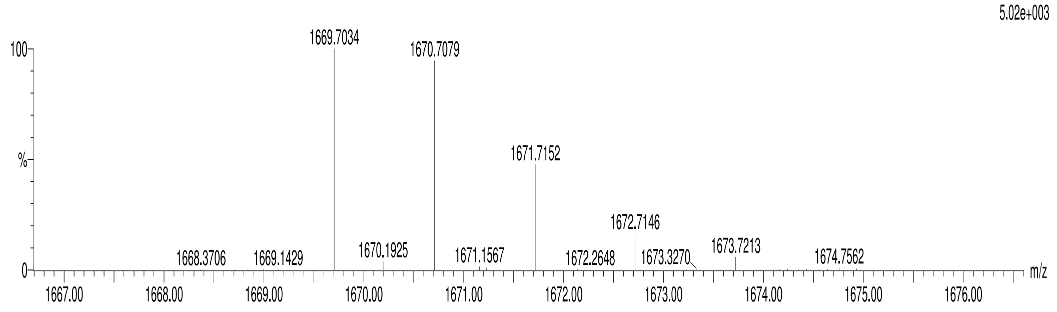

		mDa	PPM	DBE			
Minimum:				-1.5			
Maximum:		5.0	20.0	400.0			

Mass	Calc. Mass	mDa	PPM	DBE	i-FIT	i-FIT (Norm)	Formula
1669.7034	1669.7022	1.2	0.7	34.5	77.0	0.4	C76 H101 N16 O27
	1669.7035	-0.1	-0.1	39.5	77.7	1.2	C77 H97 N20 O23
	1669.7049	-1.5	-0.9	33.5	79.7	3.1	C80 H105 N10 O29

S66 Table S8. Calculated survival percentage compare to DMSO and IC_{50} of HCT-116 Colon Carcinoma cell line by compounds **1**-**4**

Concentration(μM)	Theonellamide J (**1**)	5-*cis*-Apoa-theopalauamide (**2**)	Theonellamide K (**3**)	Theopalauamide (**4**)
100.00	92	0	0	0
56.00	105	1	0	0
30.00	104	8	0	1
20.00	94	57	1	2
10.00	101	81	1	2
5.00	97	88	17	4
3.00	93	88	72	35
2.00	107	82	95	76
1.00	107	89	95	84
0.56	89	90	107	95
0.32	84	84	100	90
0.18	101	90	94	86
0.10	95	93	113	83
0.06	91	92	110	96
IC_{50} (SEM)	>100	21.8 (+/-0.7)	3.5 (+/-0.2)	2.8 (+/-0.9)

S67 Figure S59. Inhibition curve of HCT-116 Colon Carcinoma cell line by 5-*cis*-Apoa-theopalauamide (**2**)

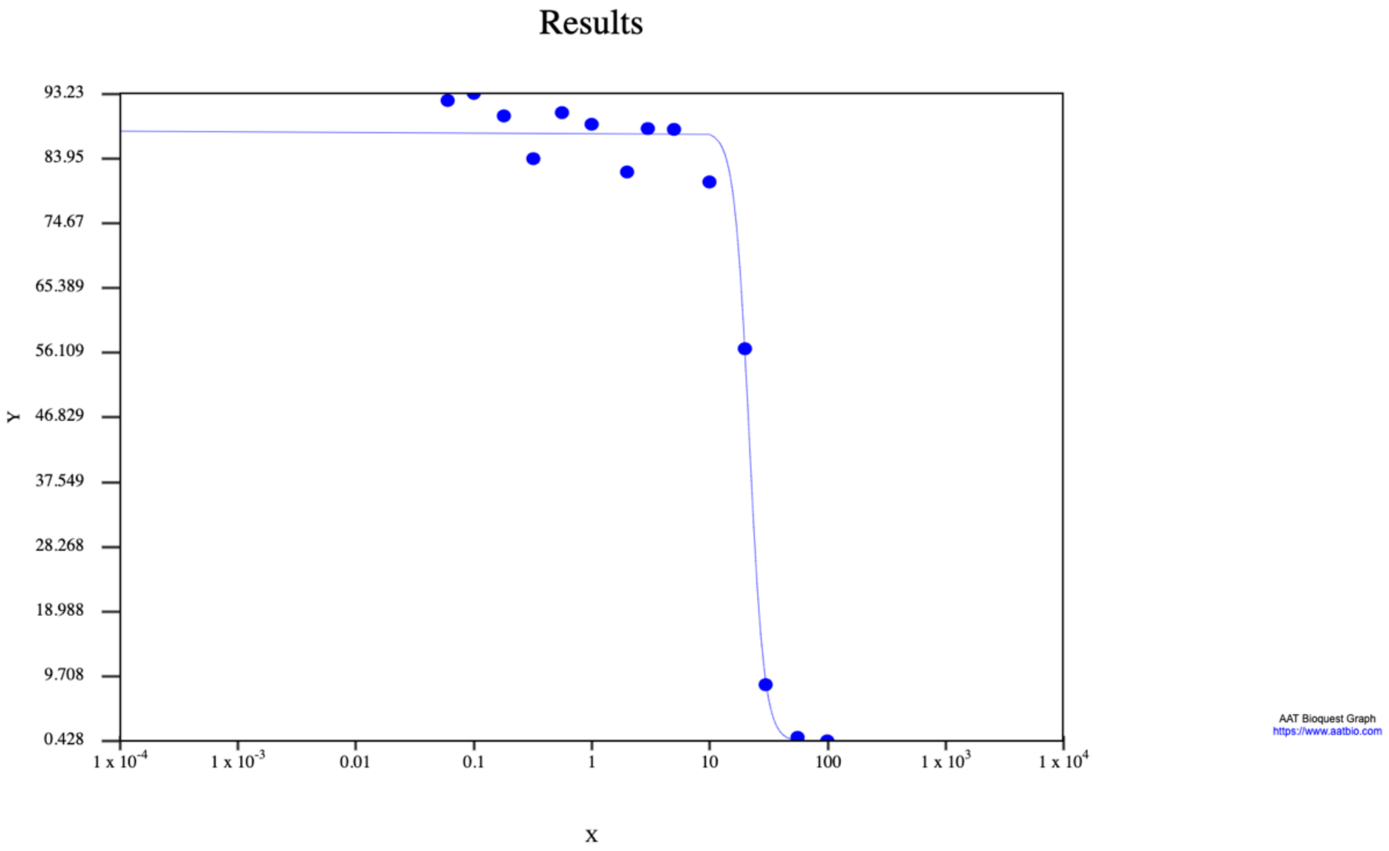

S68 Figure S60. Inhibition curve of HCT-116 Colon Carcinoma cell line by theonellamide K (**3**)

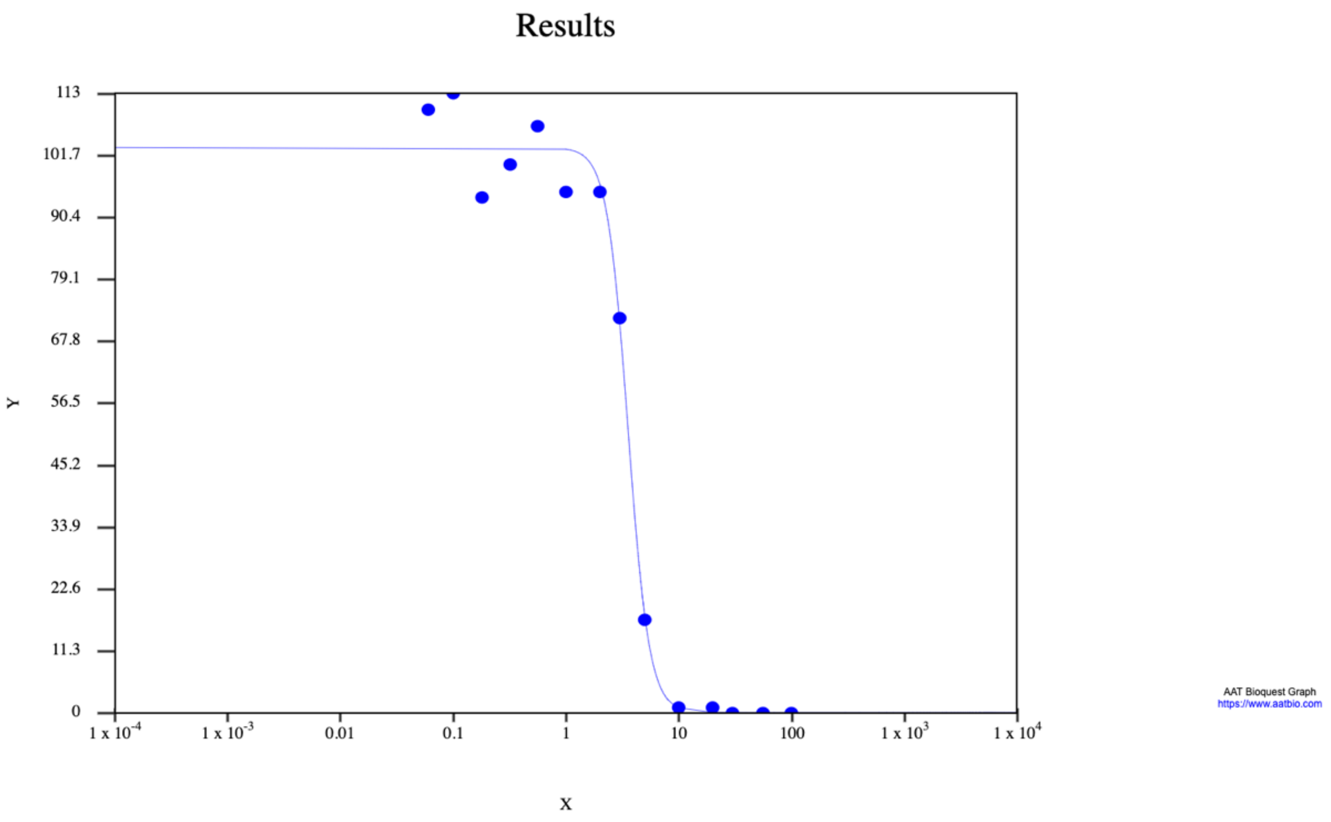

S69 Figure S61. Inhibition curve of HCT-116 Colon Carcinoma cell line by theopalauamide (**4**)

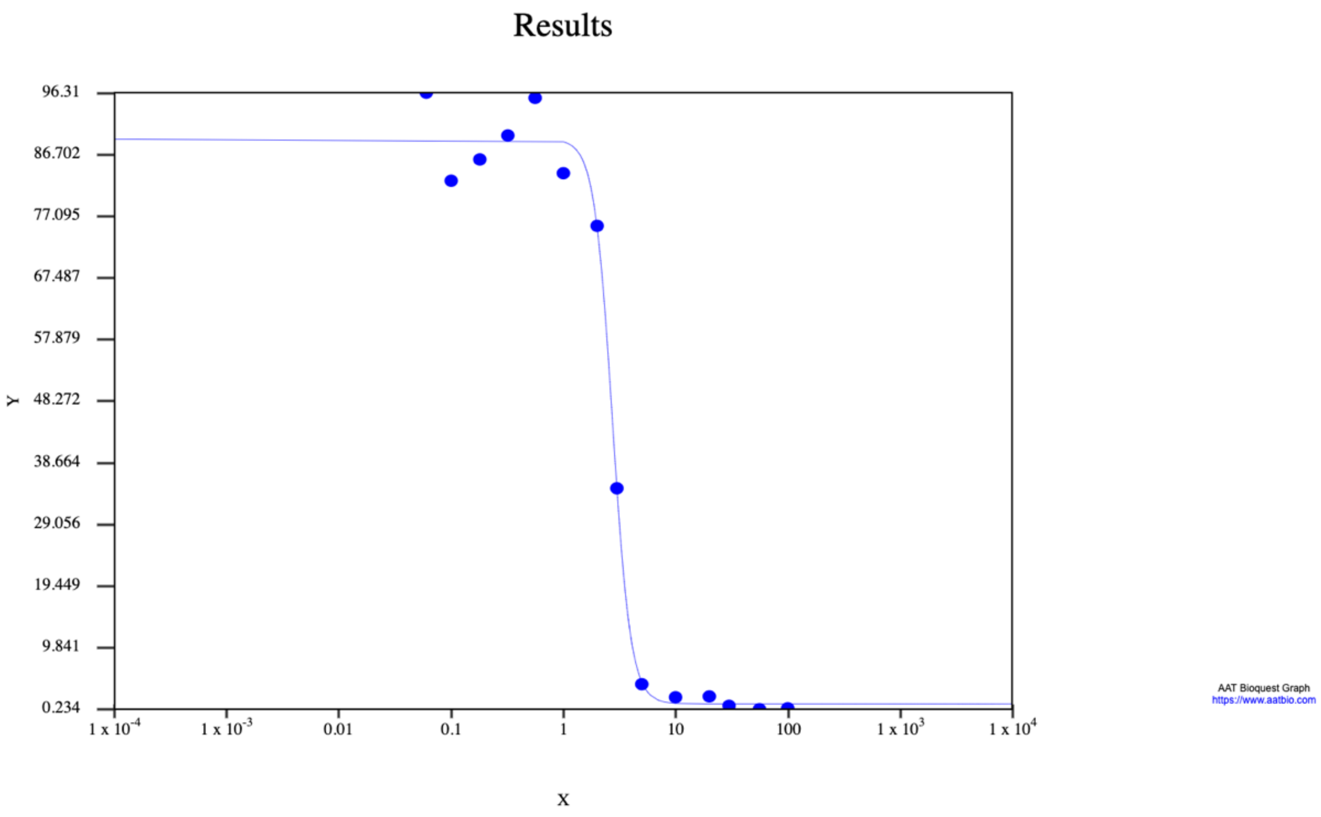

S70 Figure S62. Under water photograph of *Theonella swinhoei*

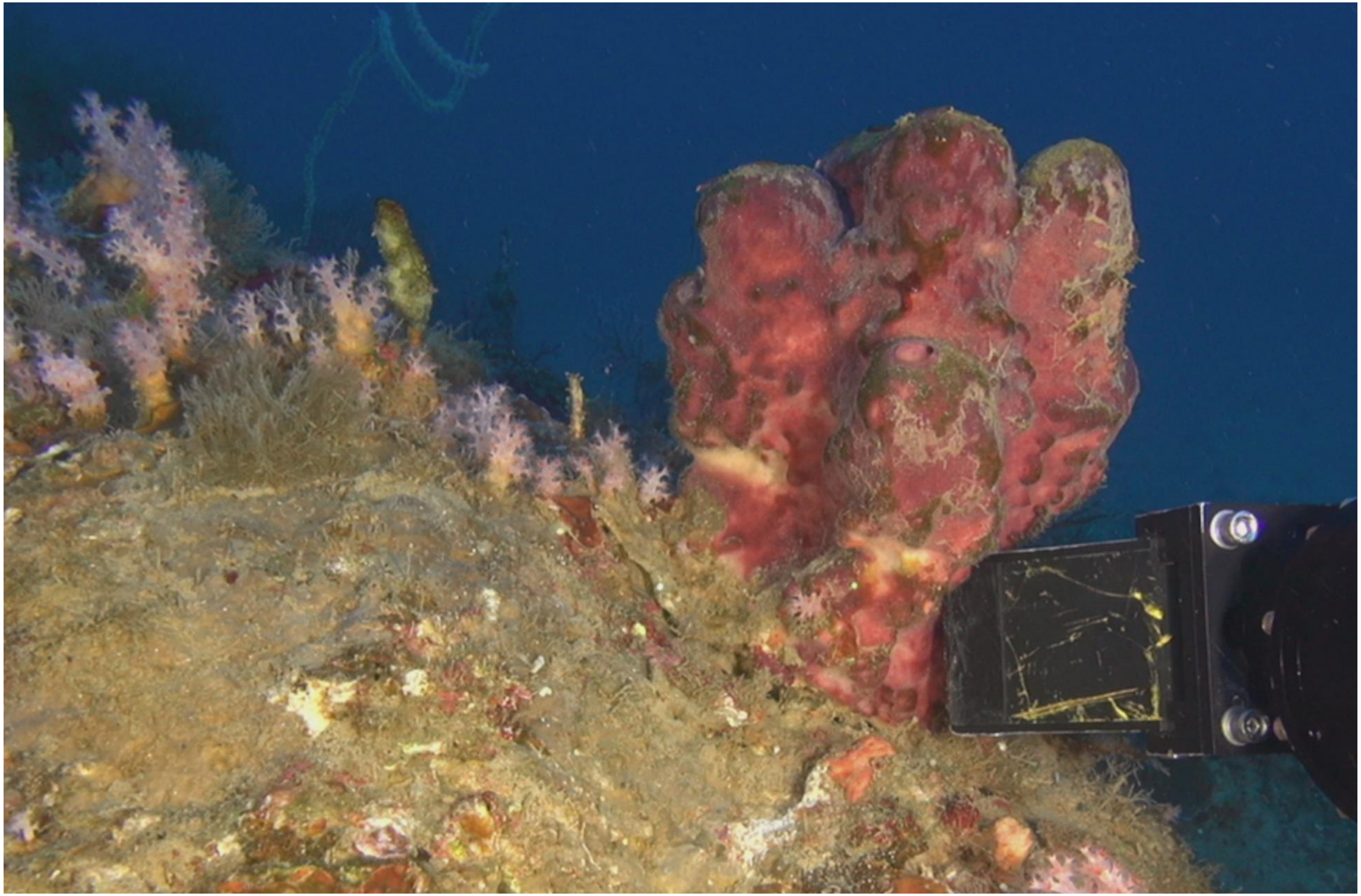

Supplement: Supplementary file 1 [file marinedrugs-20-00031-s001.zip › marinedrugs-1529407-supplementary.pdf]
